# Supplementary figures and images for: Progesterone induces meiosis through two obligate co-receptors with PLA2 activity
Source: eLife. 2025 Jan 28;13:RP92635. doi: 10.7554/eLife.92635 (PMC11774516; doi:10.7554/eLife.92635)

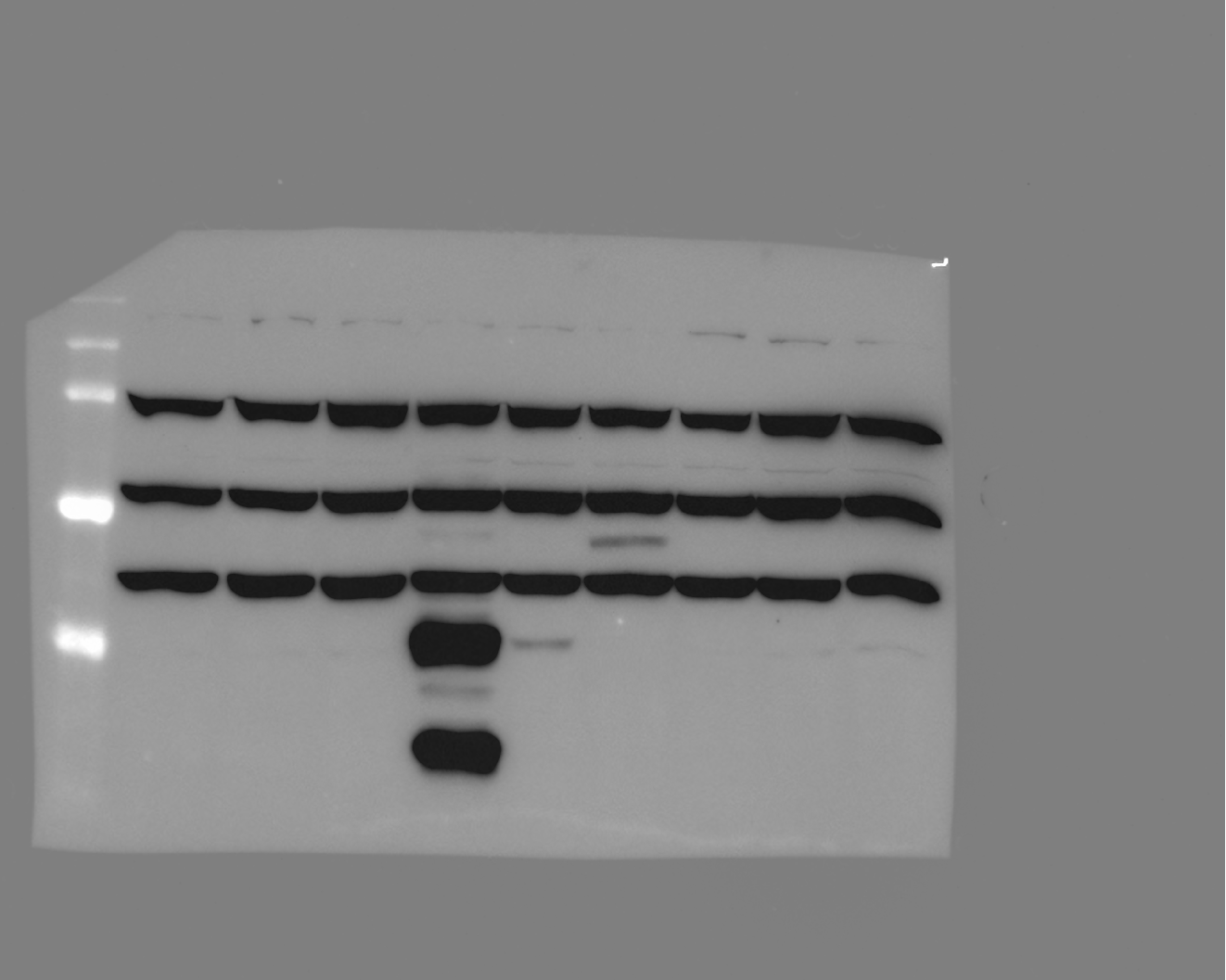

Supplement: Figure 1—source data 1. [file elife-92635-fig1-data1.zip › Figure 1 - source data 1/Figure 1 - source data 1D - RAW/ABHD2.tif]

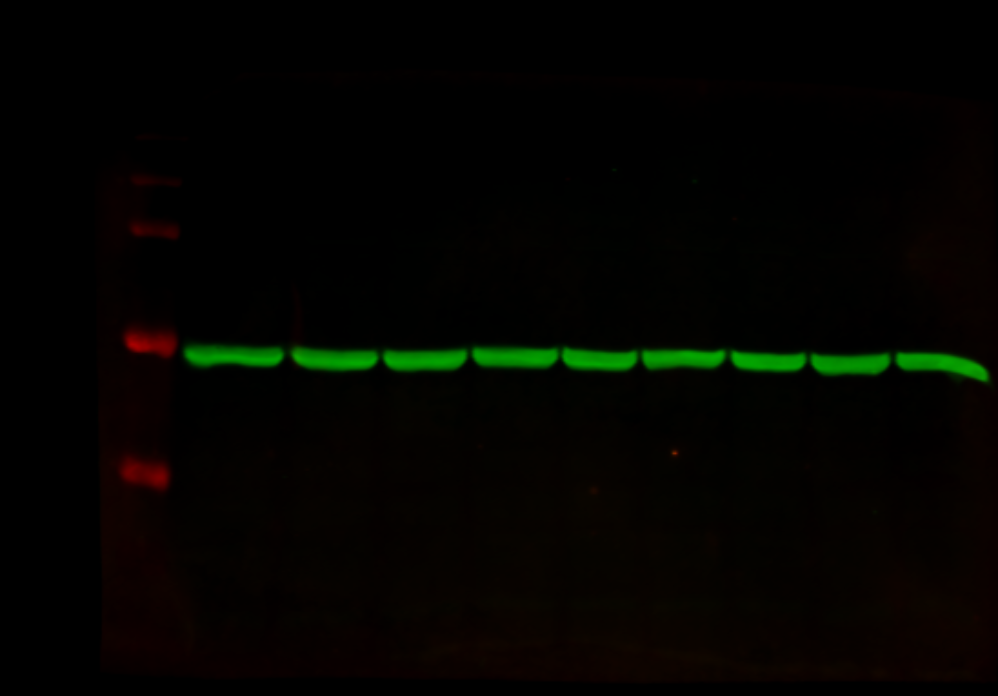

Supplement: Figure 1—source data 1. [file elife-92635-fig1-data1.zip › Figure 1 - source data 1/Figure 1 - source data 1D - RAW/Tubulin M800.tif]

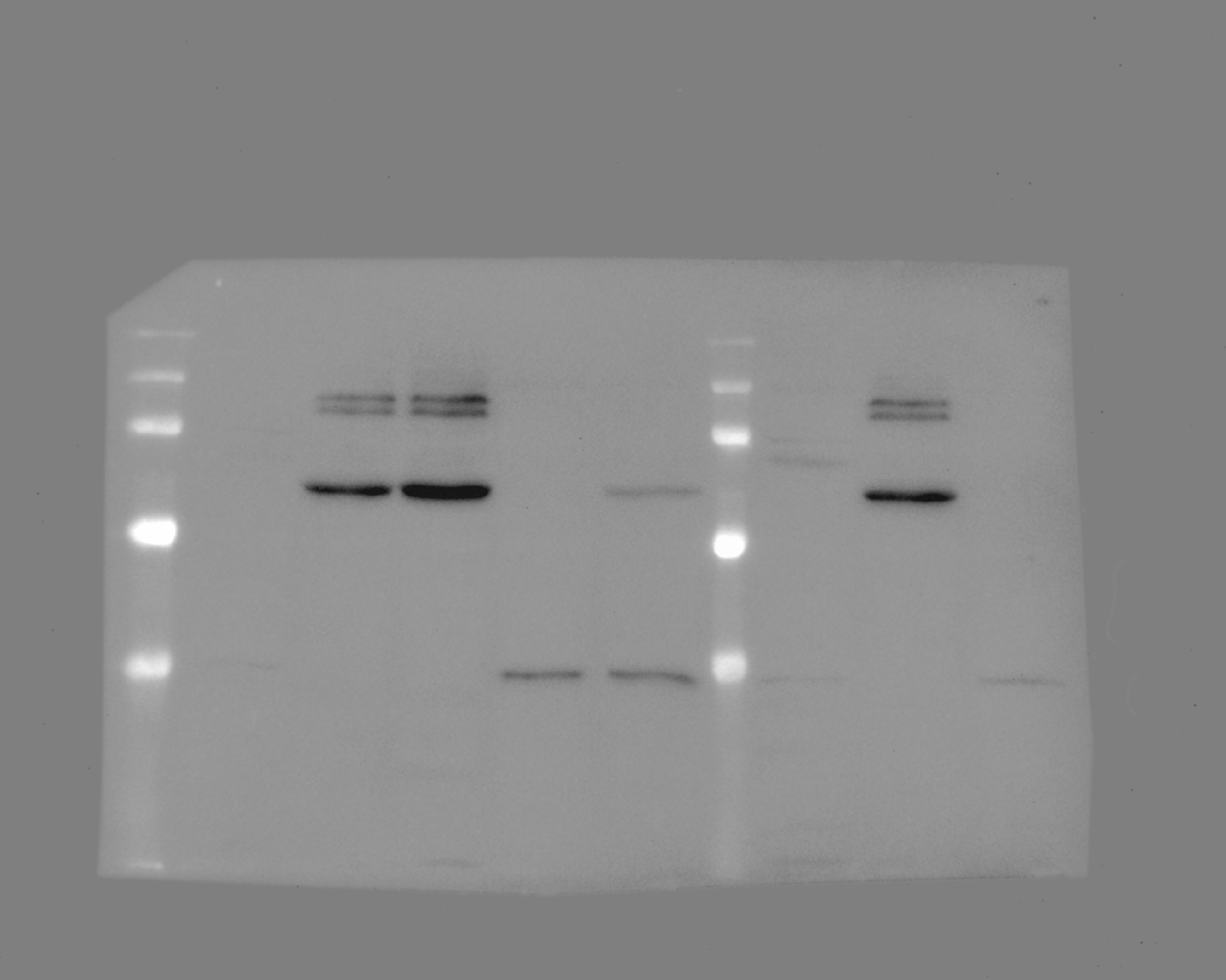

Supplement: Figure 1—source data 1. [file elife-92635-fig1-data1.zip › Figure 1 - source data 1/Figure 1 - source data 1F - RAW/p-cdc2 and p-plk1.tif]

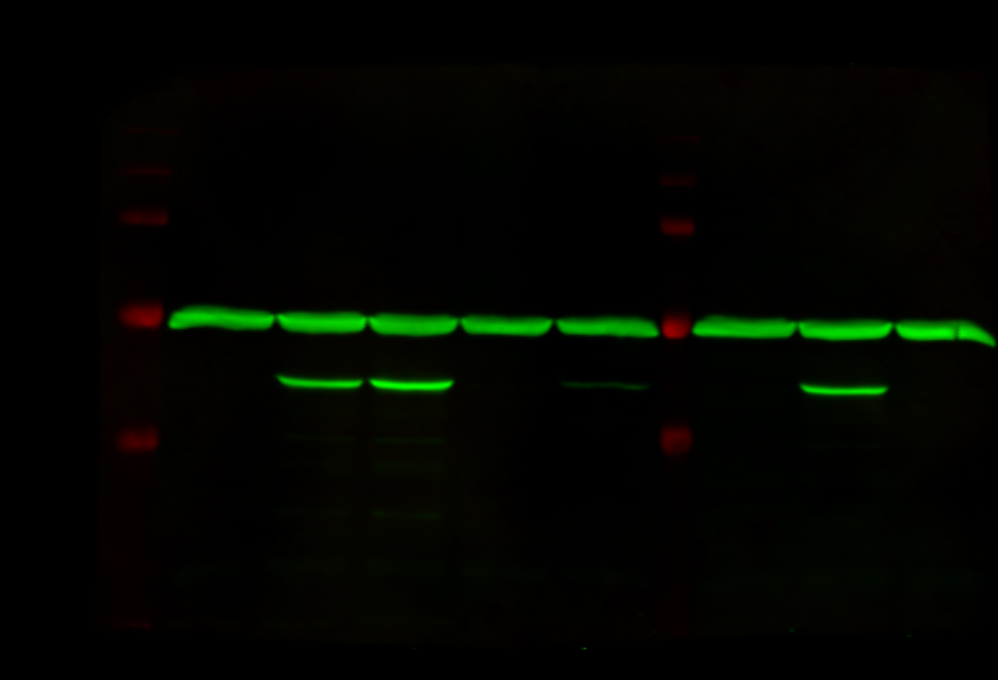

Supplement: Figure 1—source data 1. [file elife-92635-fig1-data1.zip › Figure 1 - source data 1/Figure 1 - source data 1F - RAW/p-MAPK and Tubulin M800.tif]

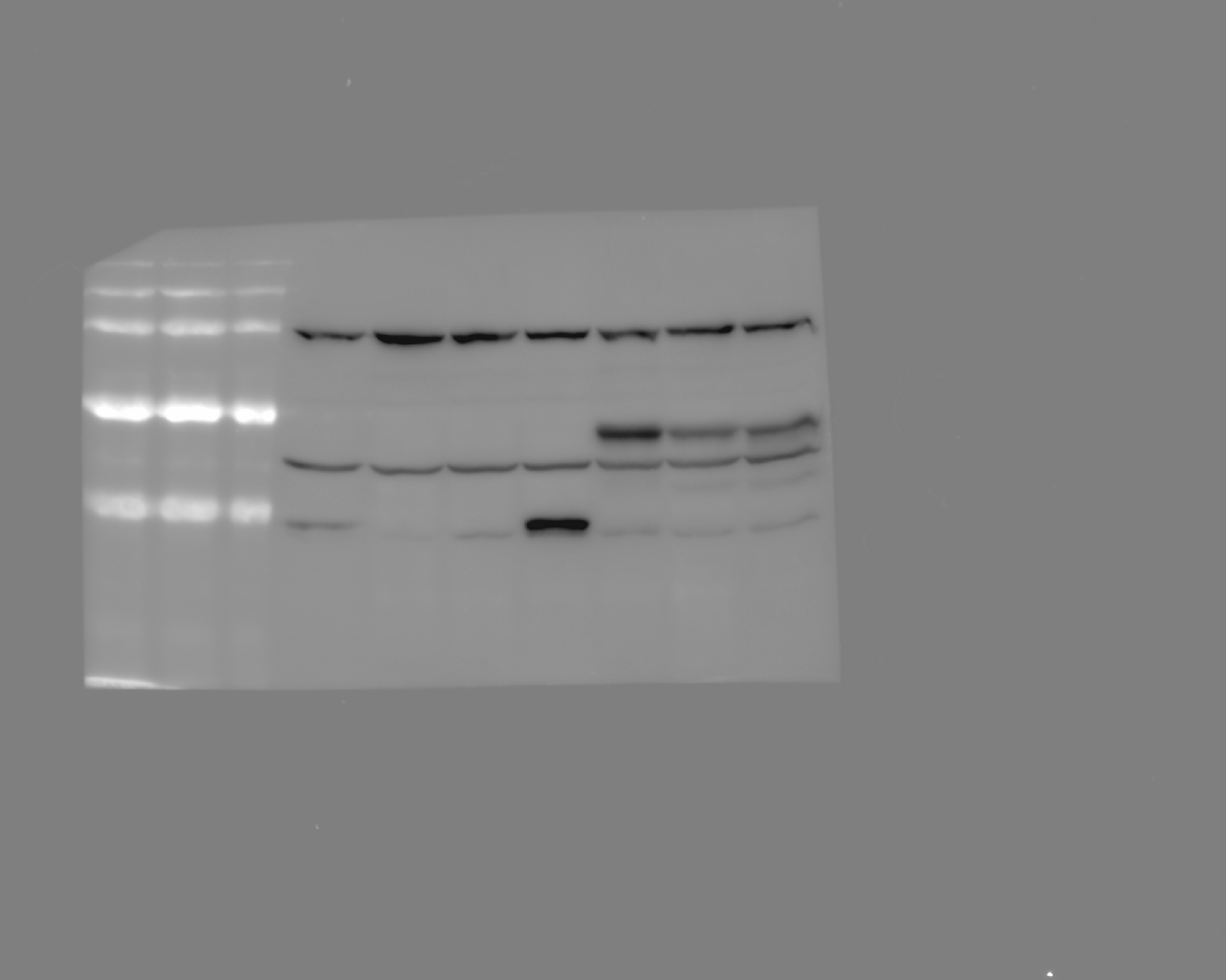

Supplement: Figure 1—source data 1. [file elife-92635-fig1-data1.zip › Figure 1 - source data 1/Figure 1 - source data 1J - RAW/ABHD2.tif]

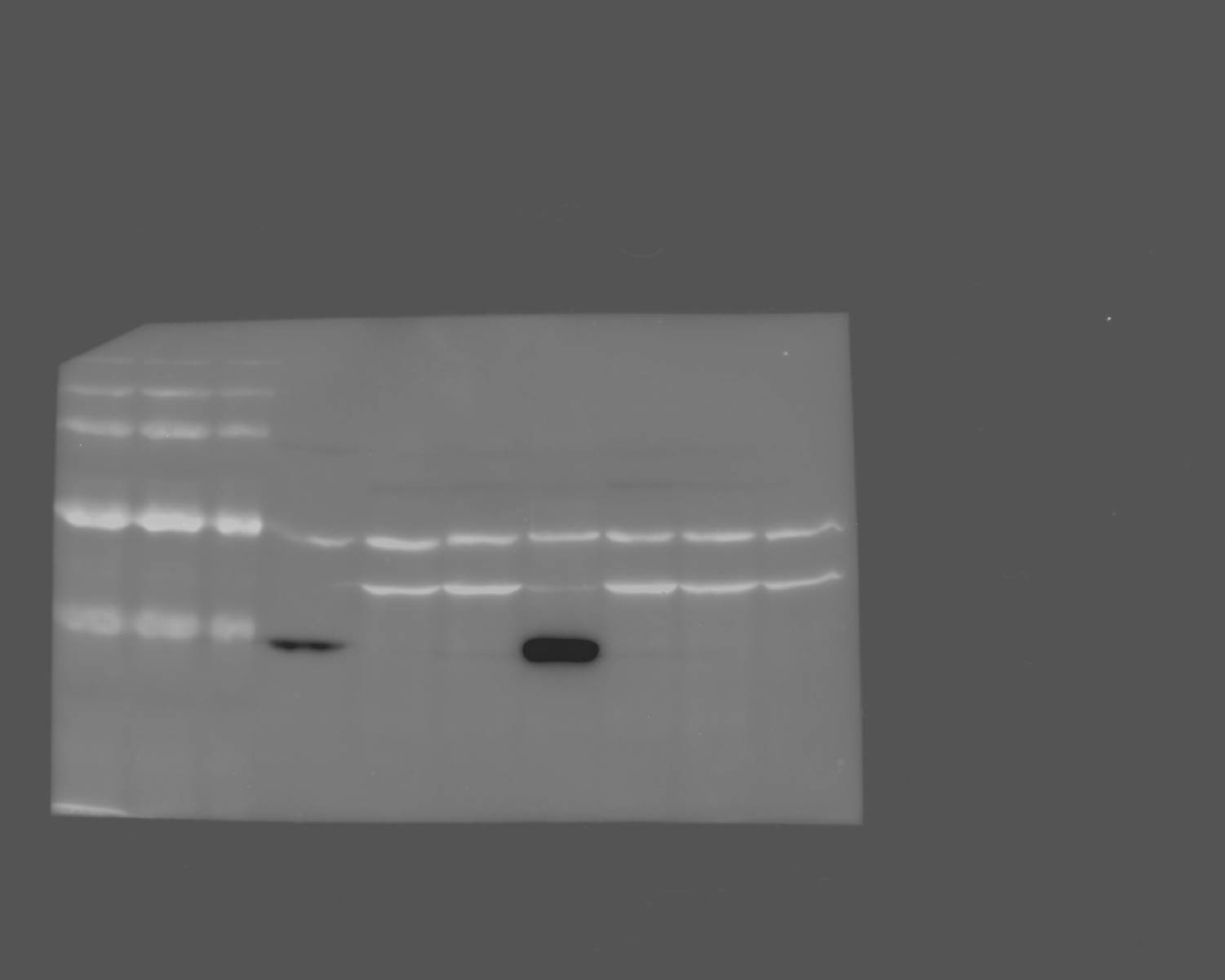

Supplement: Figure 1—source data 1. [file elife-92635-fig1-data1.zip › Figure 1 - source data 1/Figure 1 - source data 1J - RAW/p-cdc2 - 20 sec.tif]

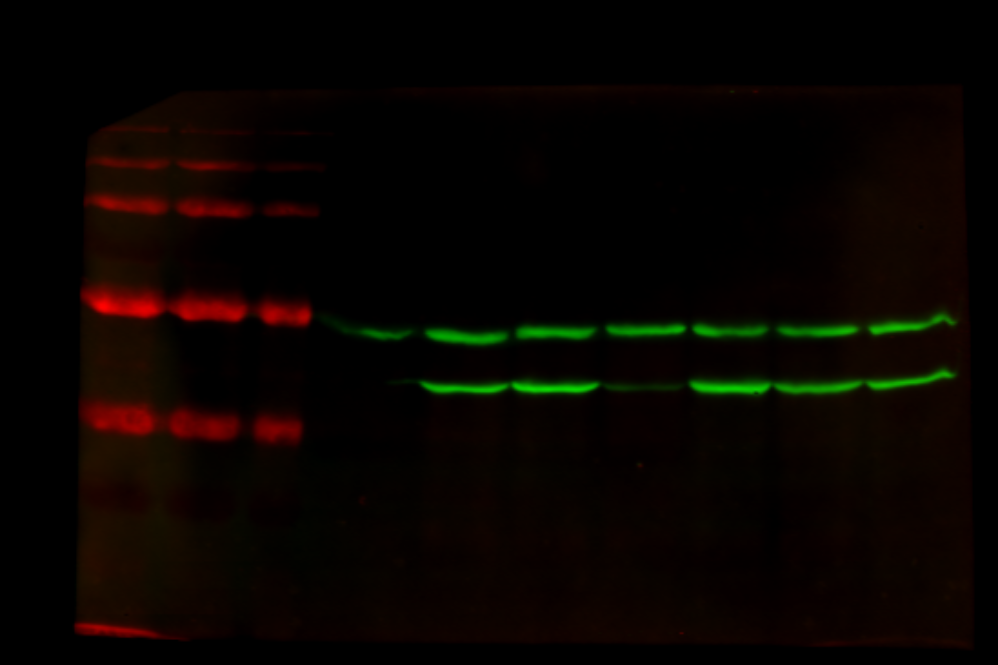

Supplement: Figure 1—source data 1. [file elife-92635-fig1-data1.zip › Figure 1 - source data 1/Figure 1 - source data 1J - RAW/p-MAPK and Tubulin M800.tif]

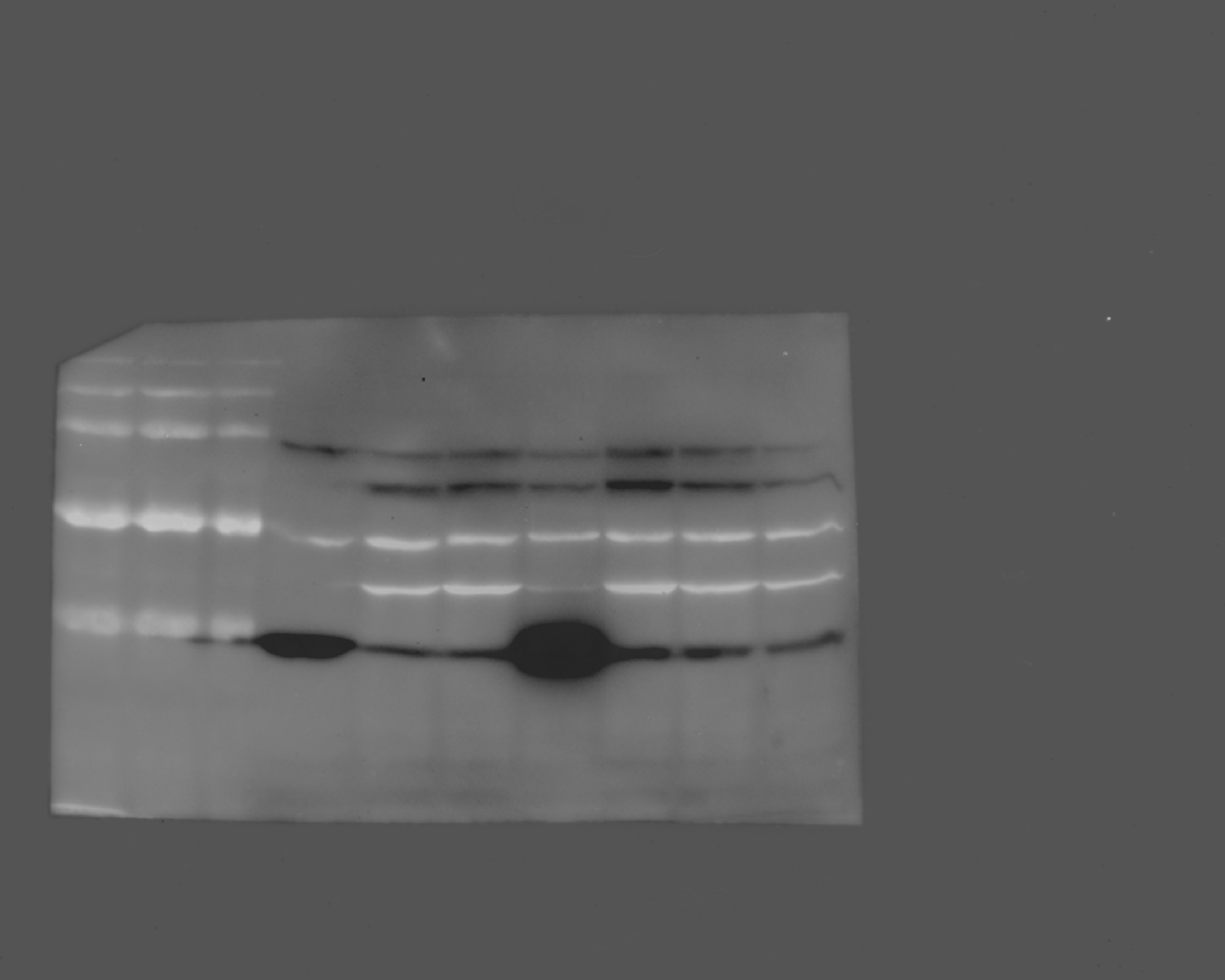

Supplement: Figure 1—source data 1. [file elife-92635-fig1-data1.zip › Figure 1 - source data 1/Figure 1 - source data 1J - RAW/p-plk1 - 1 min.tif]

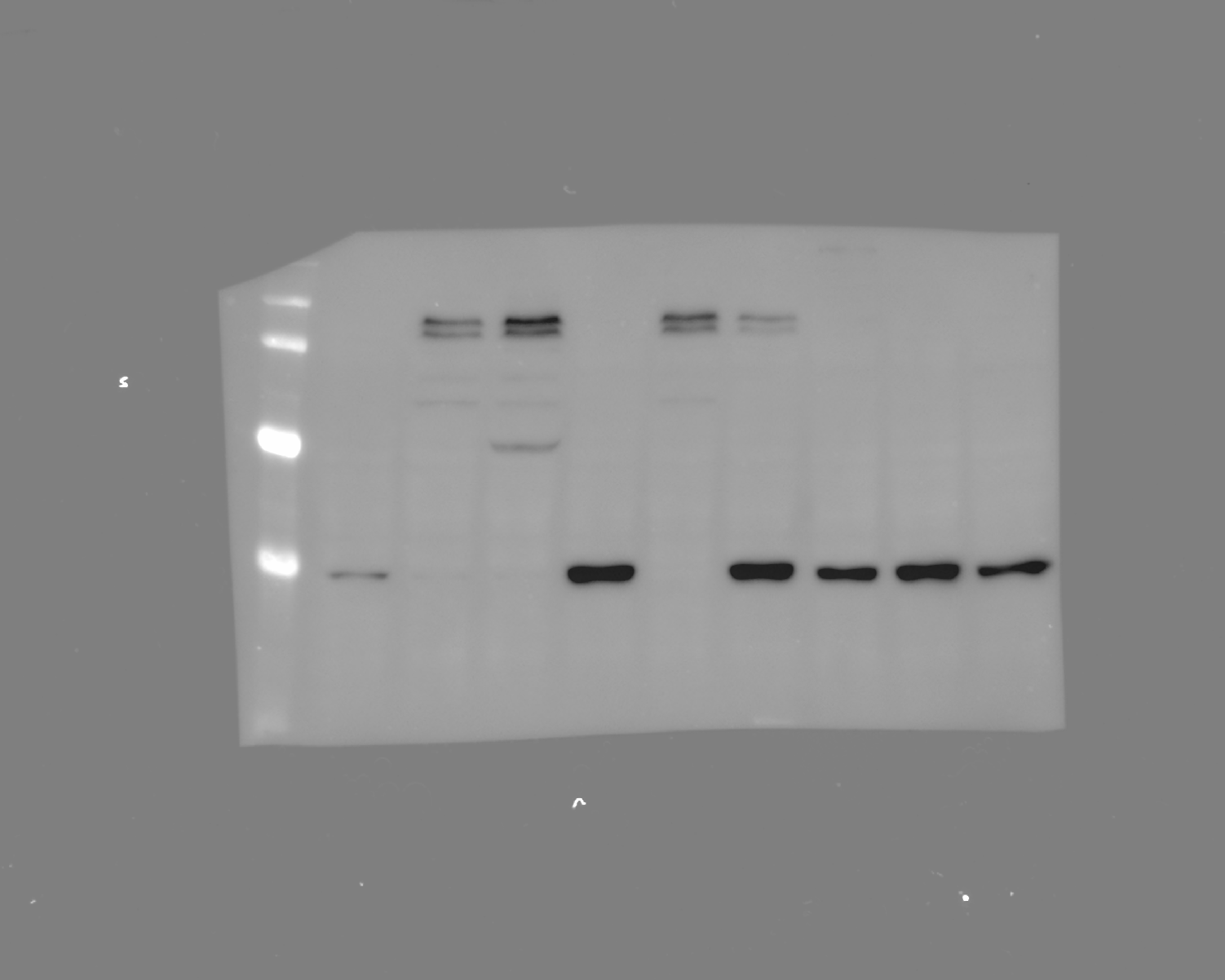

Supplement: Figure 1—source data 1. [file elife-92635-fig1-data1.zip › Figure 1 - source data 1/Figure 1 - source data 1L - RAW/p-cdc2 - 20 sec.tif]

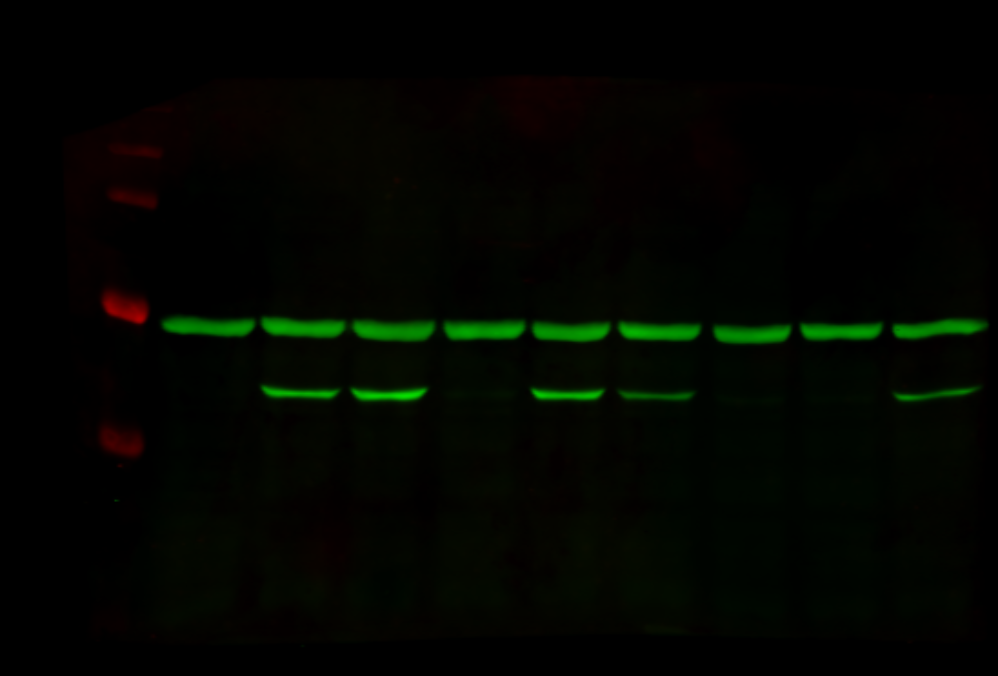

Supplement: Figure 1—source data 1. [file elife-92635-fig1-data1.zip › Figure 1 - source data 1/Figure 1 - source data 1L - RAW/p-MAPK and Tubulin M800.tif]

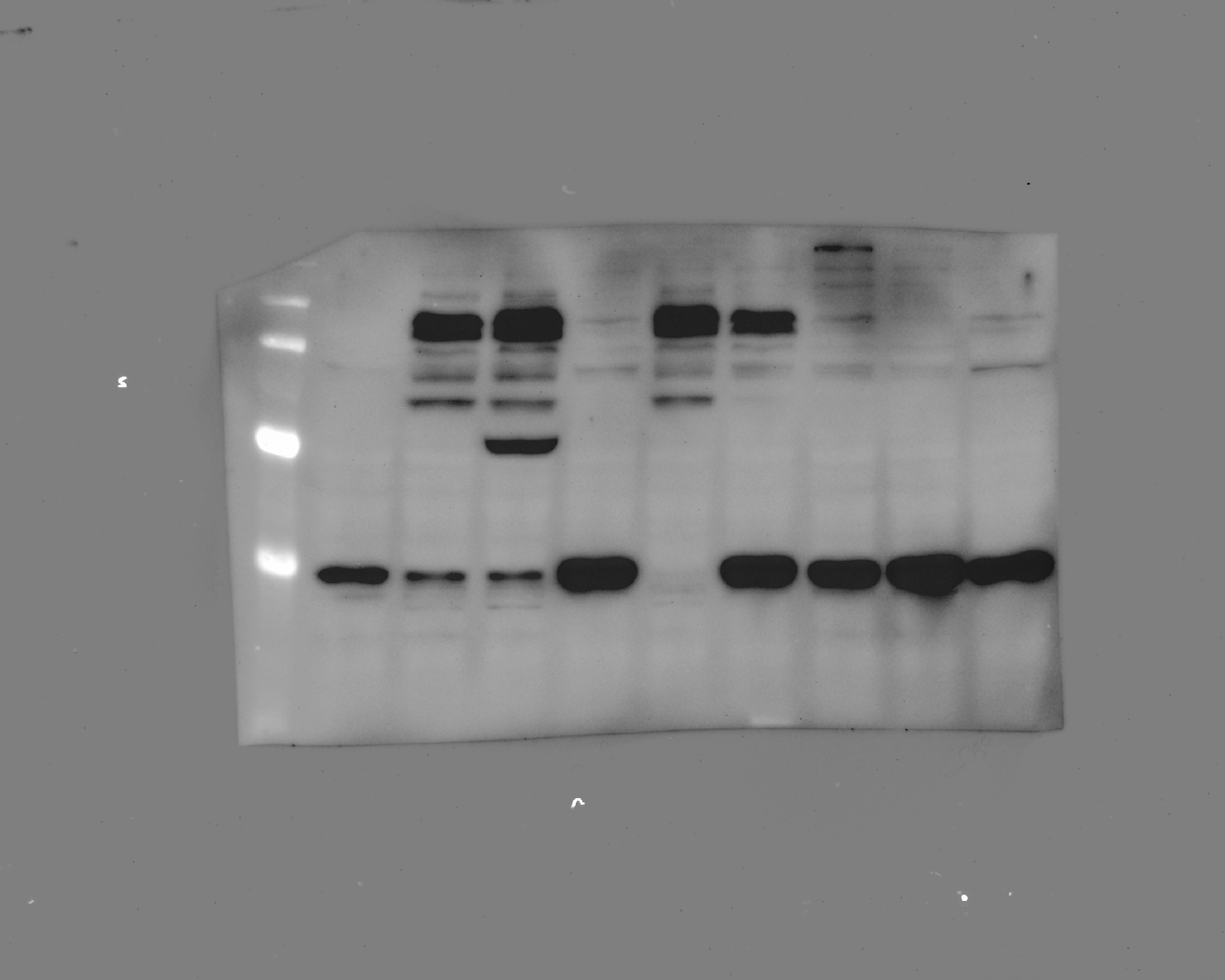

Supplement: Figure 1—source data 1. [file elife-92635-fig1-data1.zip › Figure 1 - source data 1/Figure 1 - source data 1L - RAW/p-plk1 - 1 min.tif]

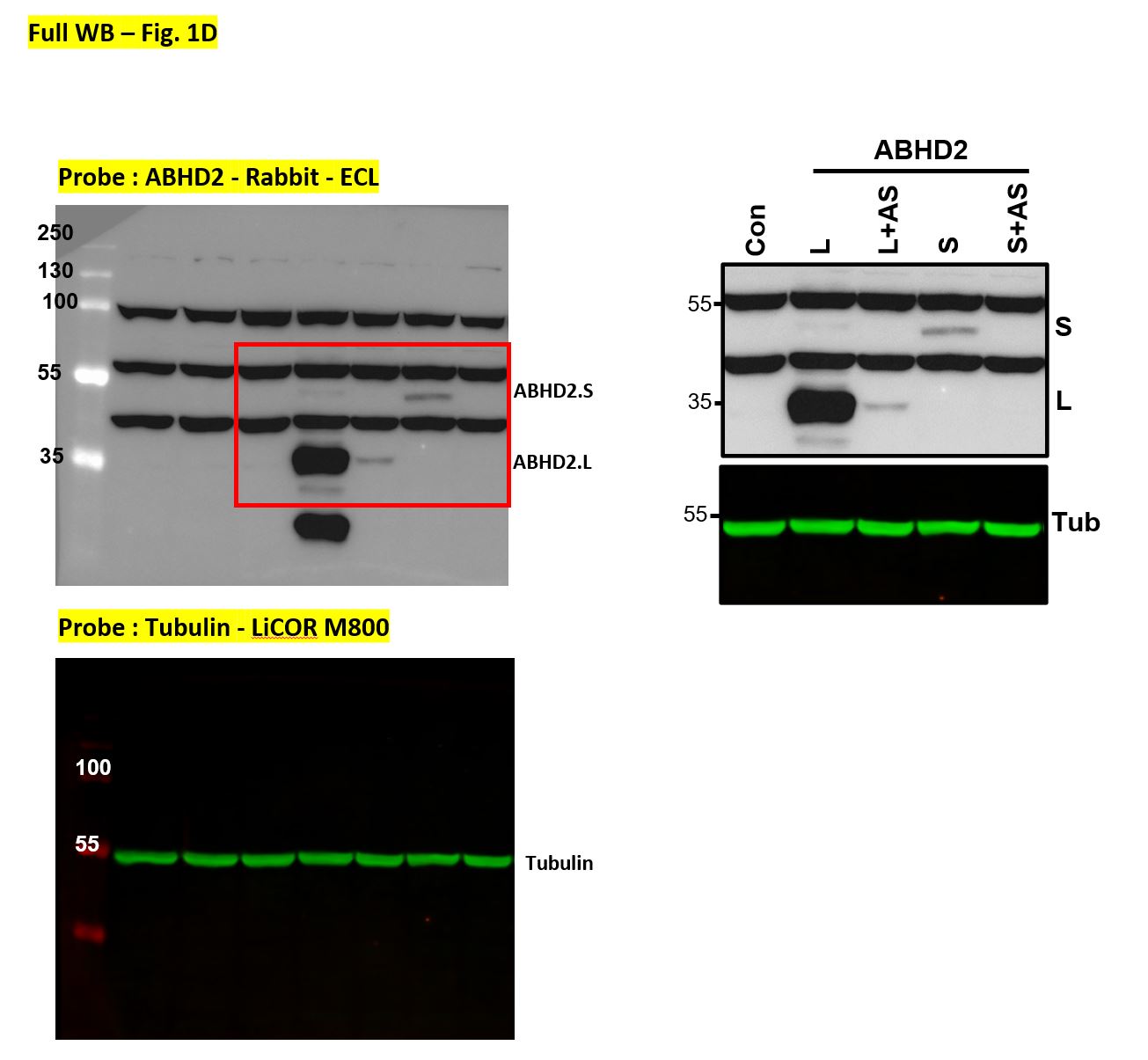

Supplement: Figure 1—source data 2. [file elife-92635-fig1-data2.zip › Figure 1 - source data 2/Figure 1 - source data 1D - Labeled/1D- Labeled.JPG]

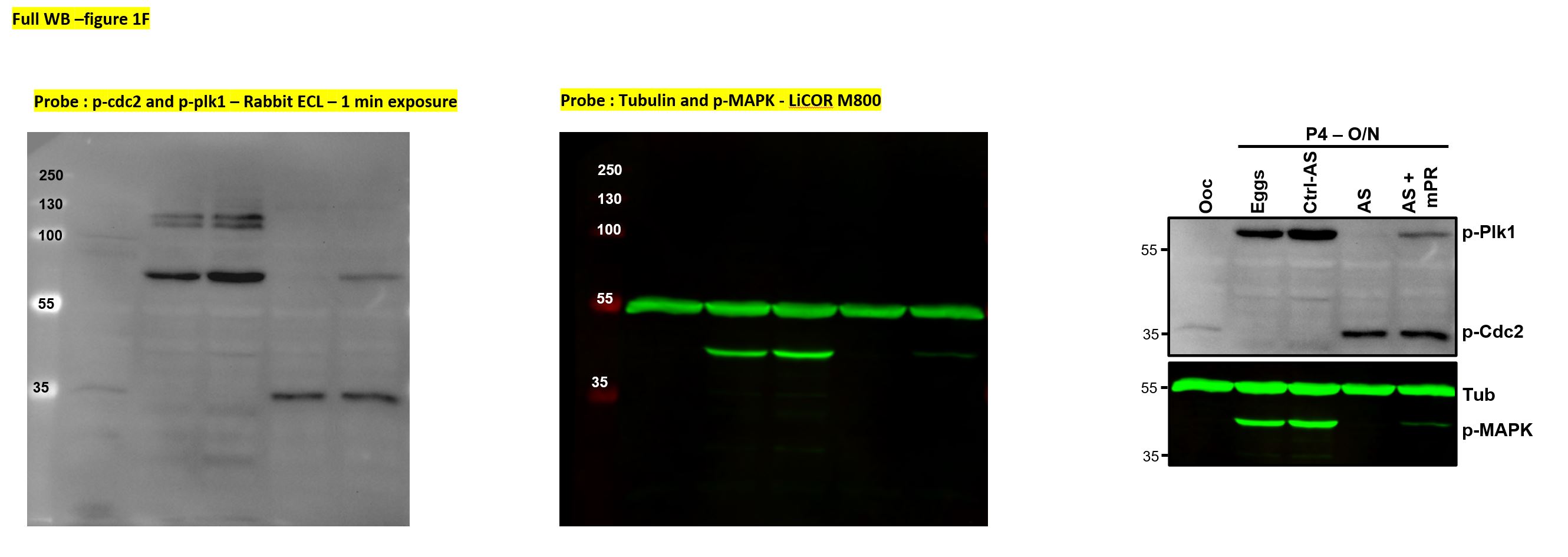

Supplement: Figure 1—source data 2. [file elife-92635-fig1-data2.zip › Figure 1 - source data 2/Figure 1 - source data 1F - Labeled/1F - Labeled.JPG]

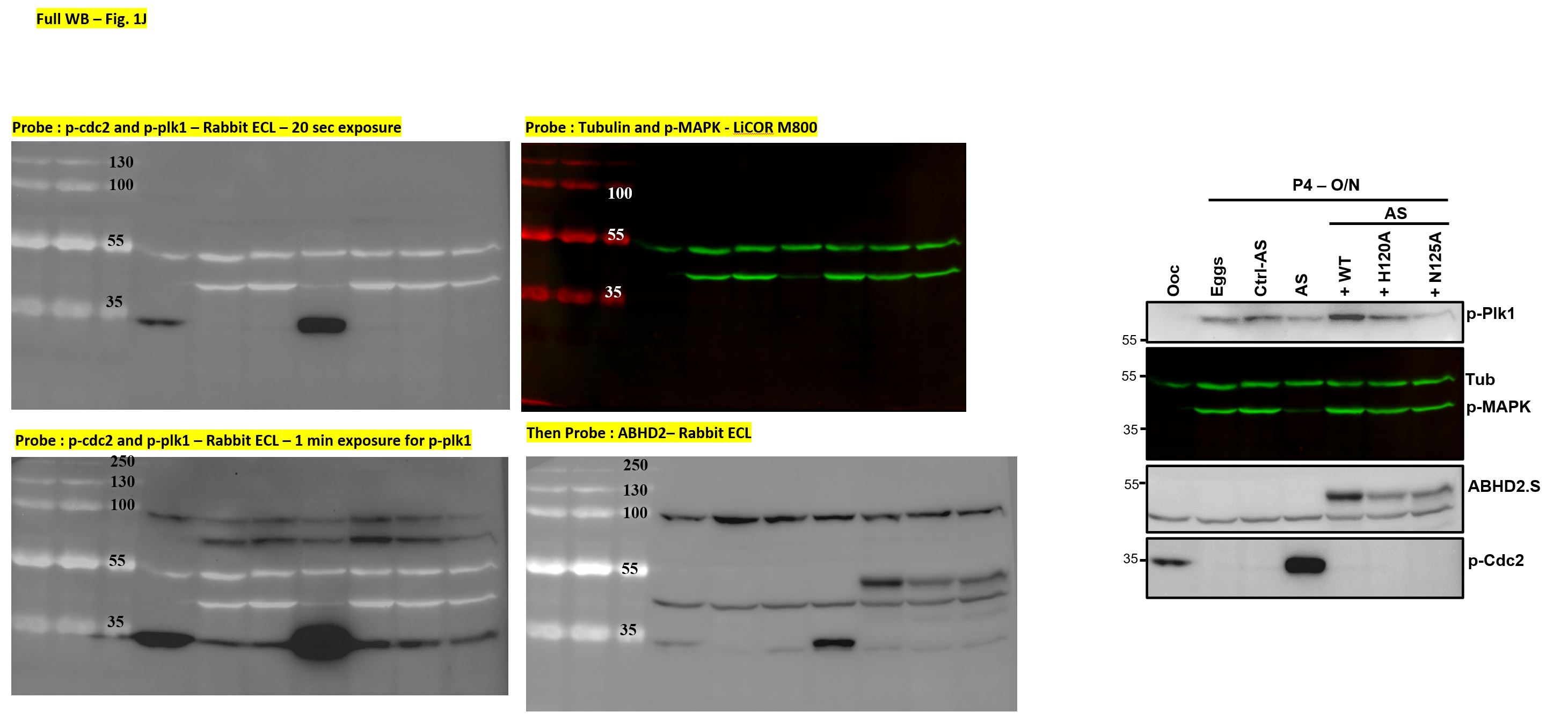

Supplement: Figure 1—source data 2. [file elife-92635-fig1-data2.zip › Figure 1 - source data 2/Figure 1 - source data 1J - Labeled/1J labeled.JPG]

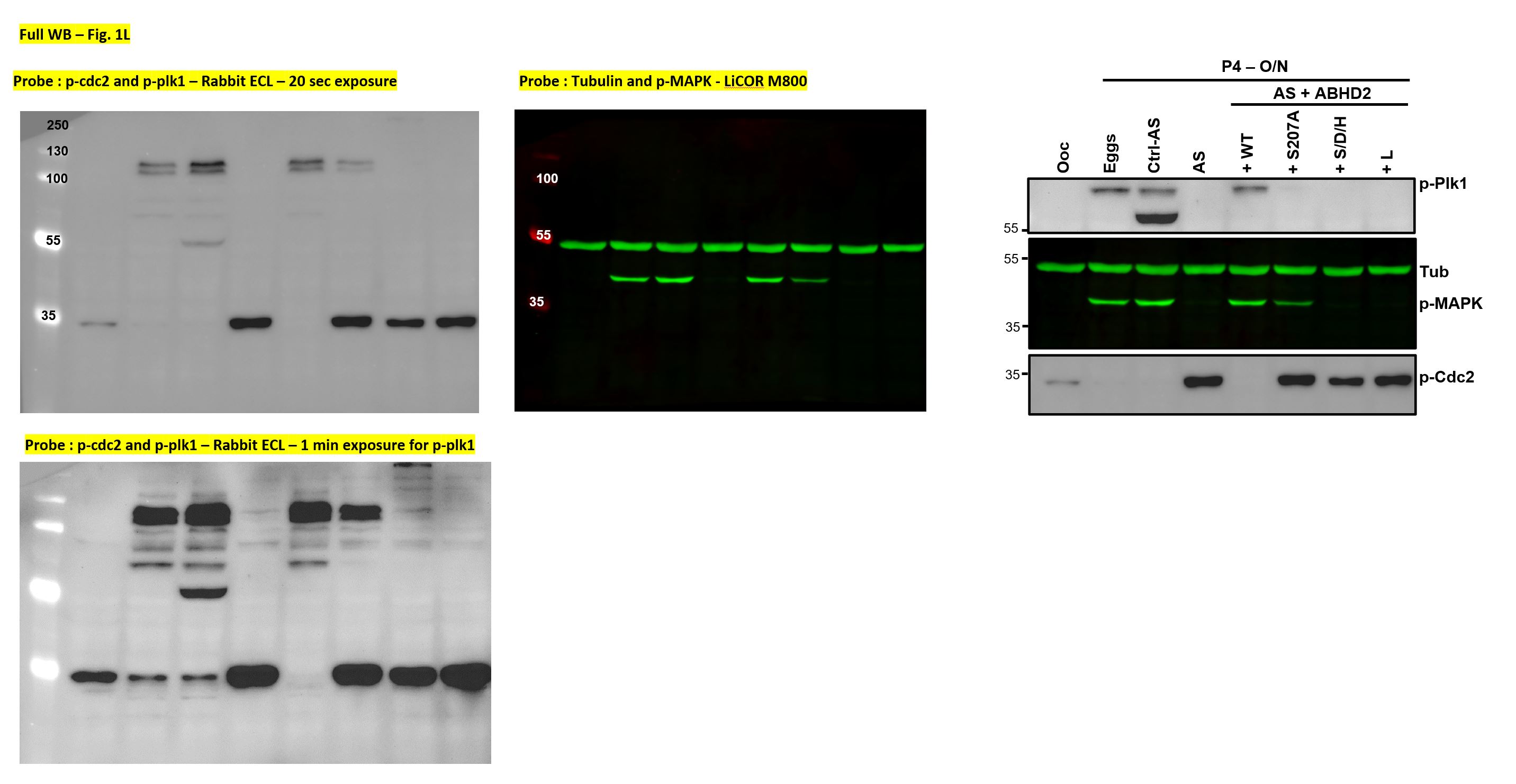

Supplement: Figure 1—source data 2. [file elife-92635-fig1-data2.zip › Figure 1 - source data 2/Figure 1 - source data 1L - Labeled/1L Labeled.JPG]

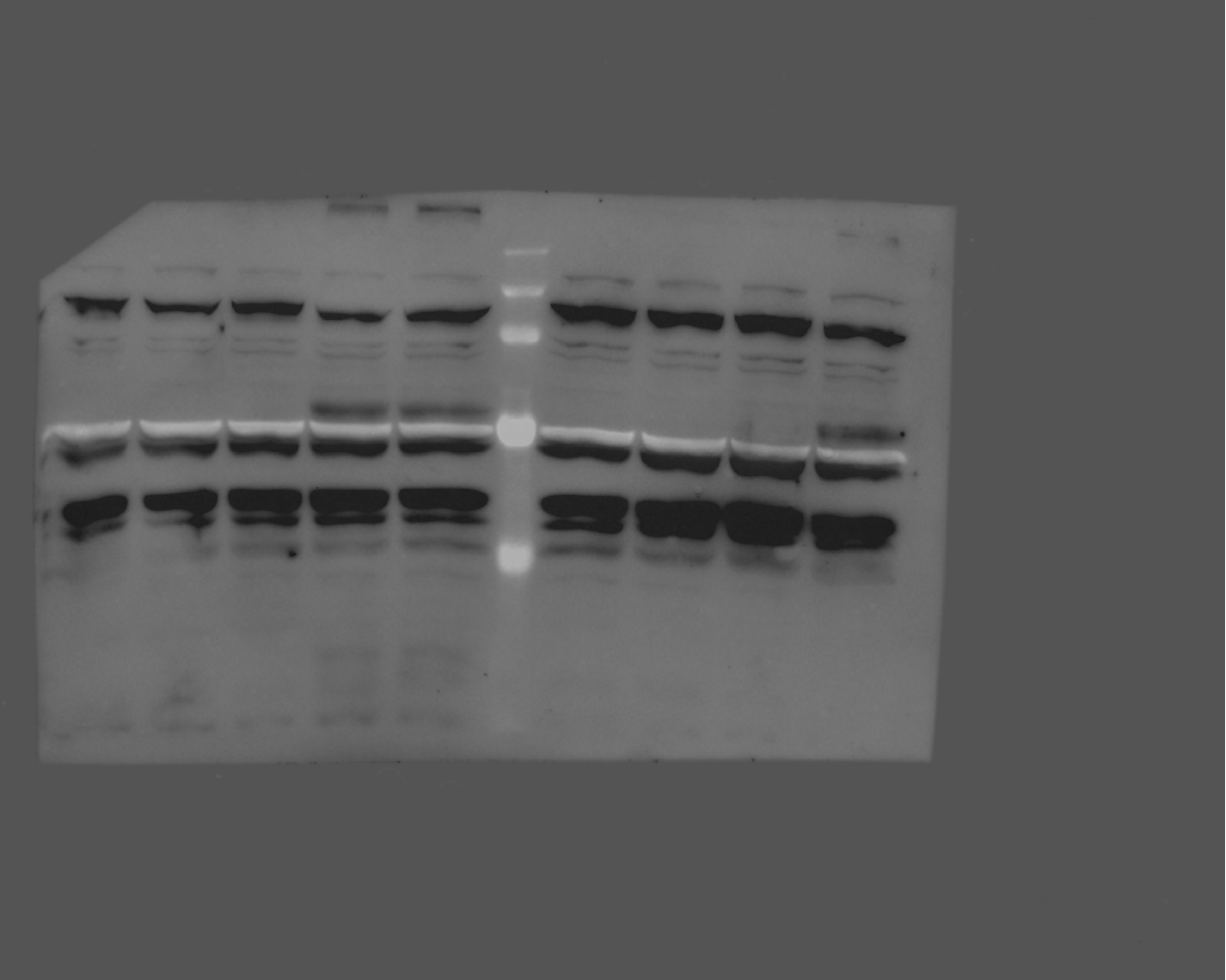

Supplement: Figure 1—figure supplement 1—source data 1. [file elife-92635-fig1-figsupp1-data1.zip › Figure 1 - Figure supplement 1 - source data 1/Figure 1 - Figure supplement 1C Raw/Tubulin and GFP.tif]

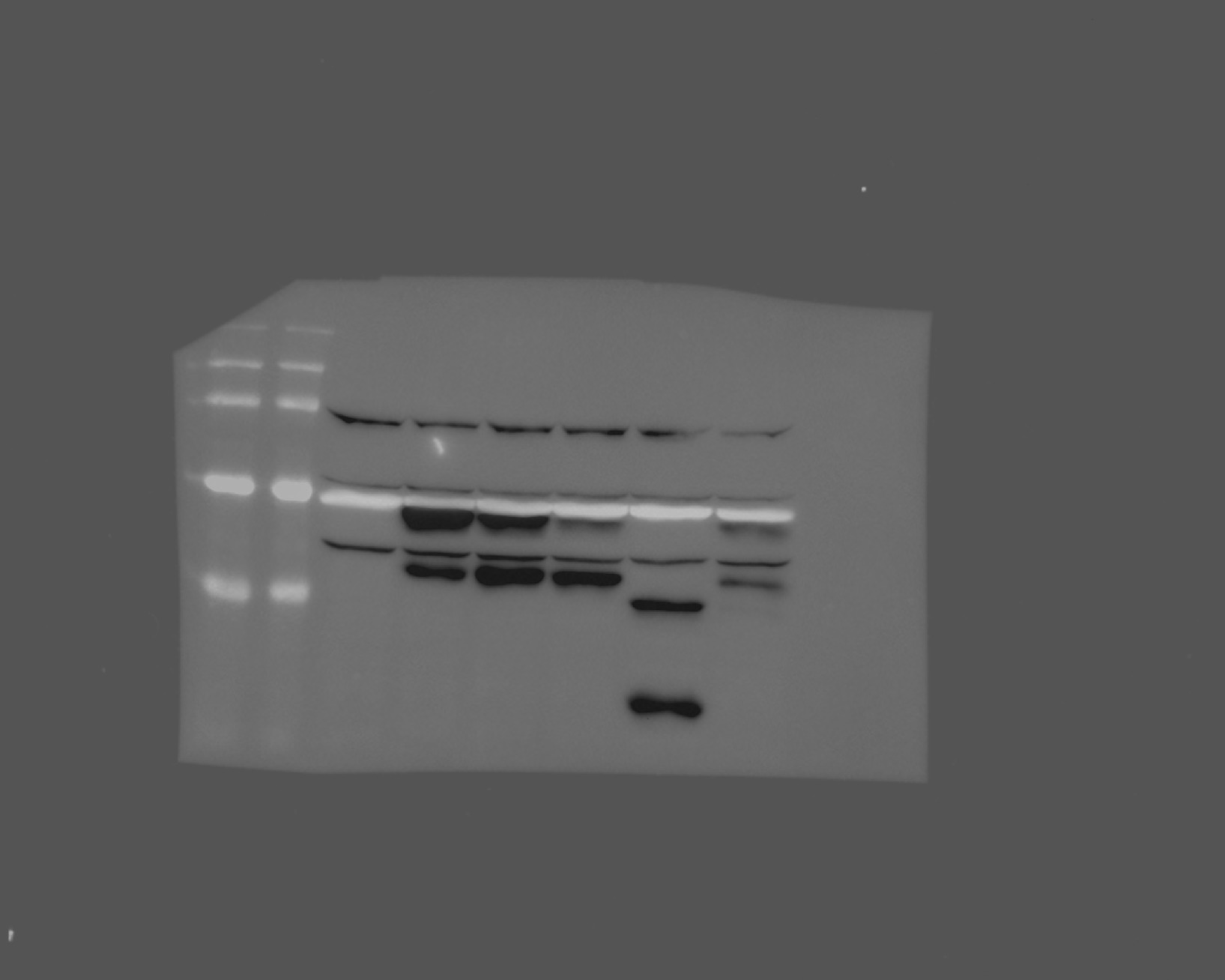

Supplement: Figure 1—figure supplement 1—source data 1. [file elife-92635-fig1-figsupp1-data1.zip › Figure 1 - Figure supplement 1 - source data 1/Figure 1 - Figure supplement 1D Raw/ABHD2 and Tubulin.tif]

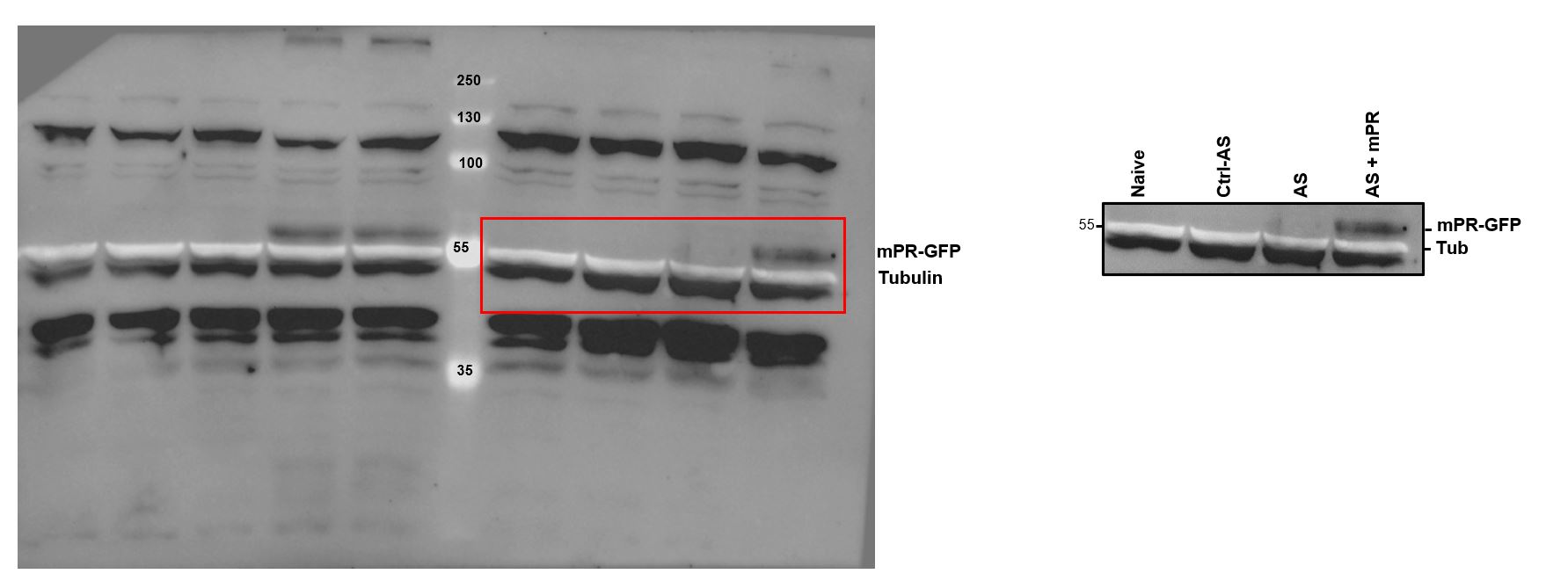

Supplement: Figure 1—figure supplement 1—source data 2. [file elife-92635-fig1-figsupp1-data2.zip › Figure 1 - Figure supplement 1 - source data 2/Figure 1 - Figure supplement 1C Labeled/Figure 1 - source data Figure supplement 1C Labeled.JPG]

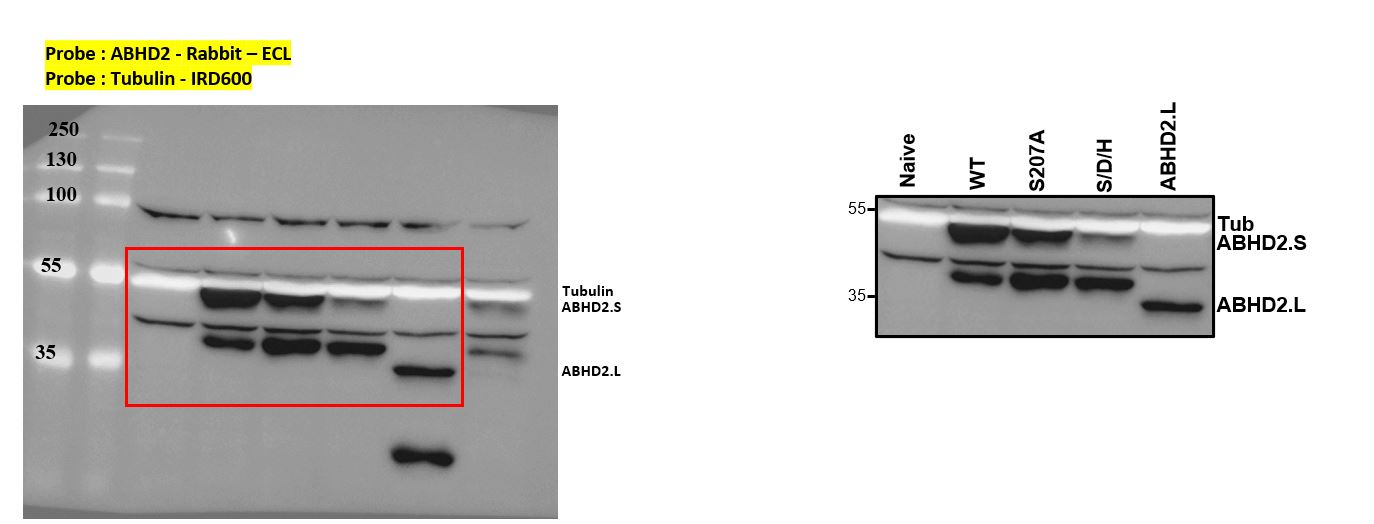

Supplement: Figure 1—figure supplement 1—source data 2. [file elife-92635-fig1-figsupp1-data2.zip › Figure 1 - Figure supplement 1 - source data 2/Figure 1 - Figure supplement 1D Labeled/Figure 1 - source data Figure supplement 1D Labeled.JPG]

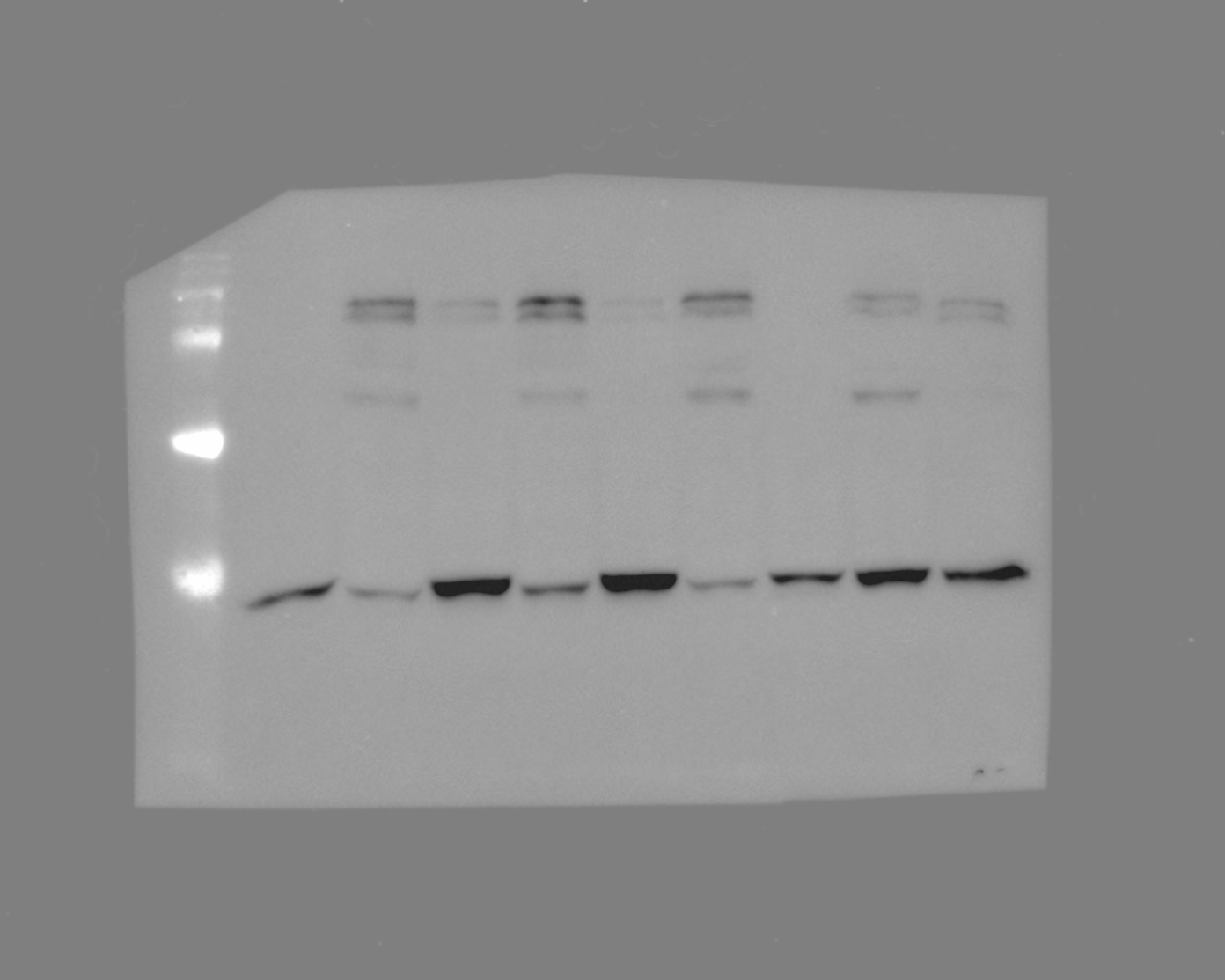

Supplement: Figure 3—source data 1. [file elife-92635-fig3-data1.zip › Figure 3 - source data 1/Figure 3 - source data 3B - RAW/p-cdc2 - 20 sec.tif]

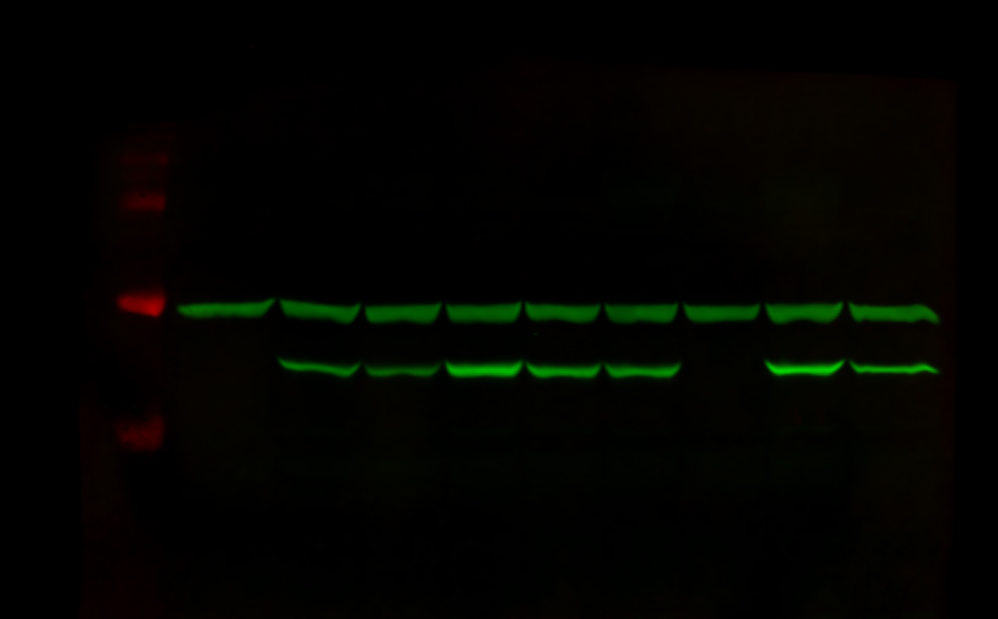

Supplement: Figure 3—source data 1. [file elife-92635-fig3-data1.zip › Figure 3 - source data 1/Figure 3 - source data 3B - RAW/p-MAPK and Tubulin M800.tif]

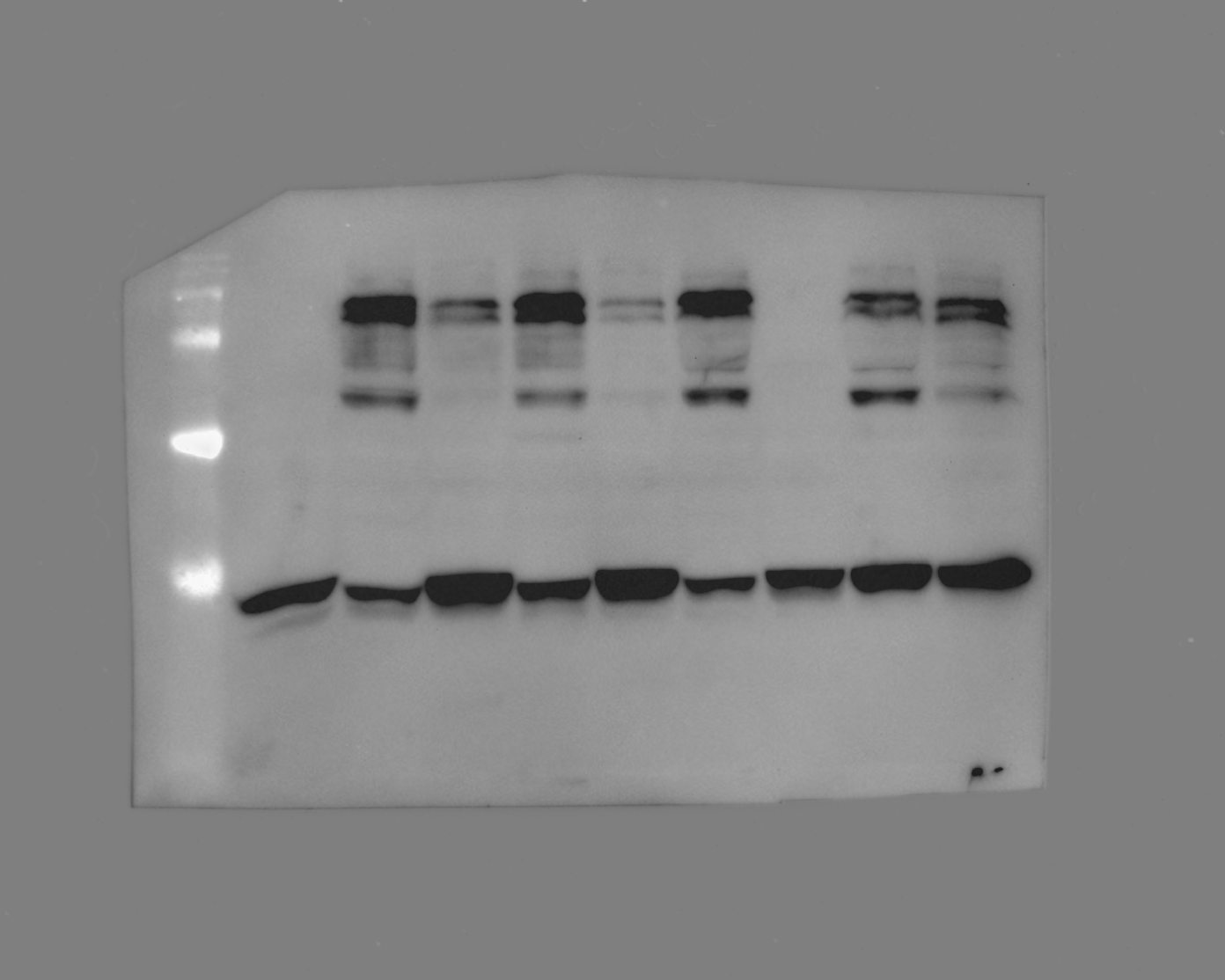

Supplement: Figure 3—source data 1. [file elife-92635-fig3-data1.zip › Figure 3 - source data 1/Figure 3 - source data 3B - RAW/p-plk1 - 1 min.tif]

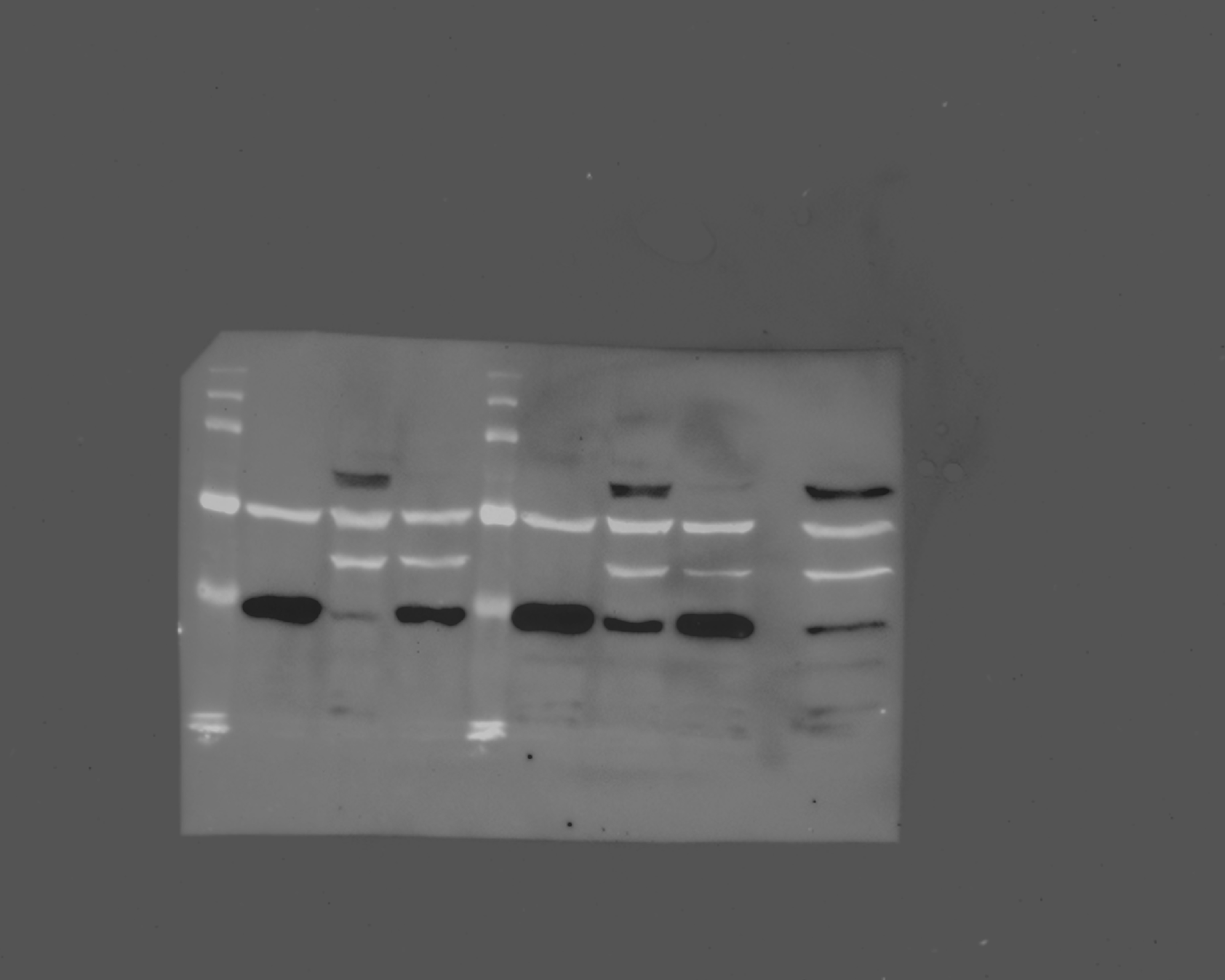

Supplement: Figure 3—source data 1. [file elife-92635-fig3-data1.zip › Figure 3 - source data 1/Figure 3 - source data 3C - RAW/p-cdc2 and p-plk1.tif]

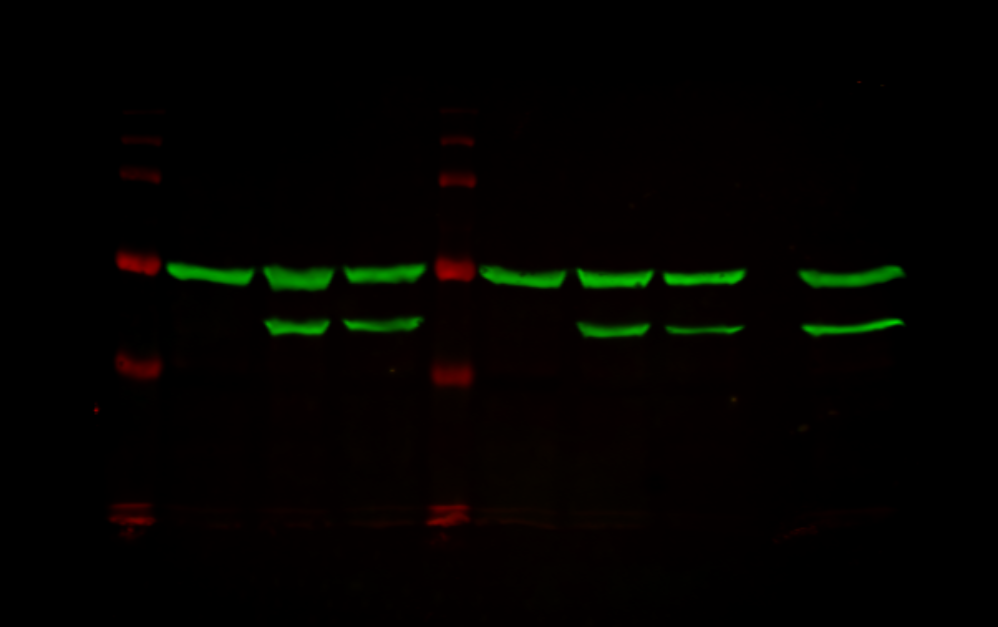

Supplement: Figure 3—source data 1. [file elife-92635-fig3-data1.zip › Figure 3 - source data 1/Figure 3 - source data 3C - RAW/p-MAPK and Tubulin M800.tif]

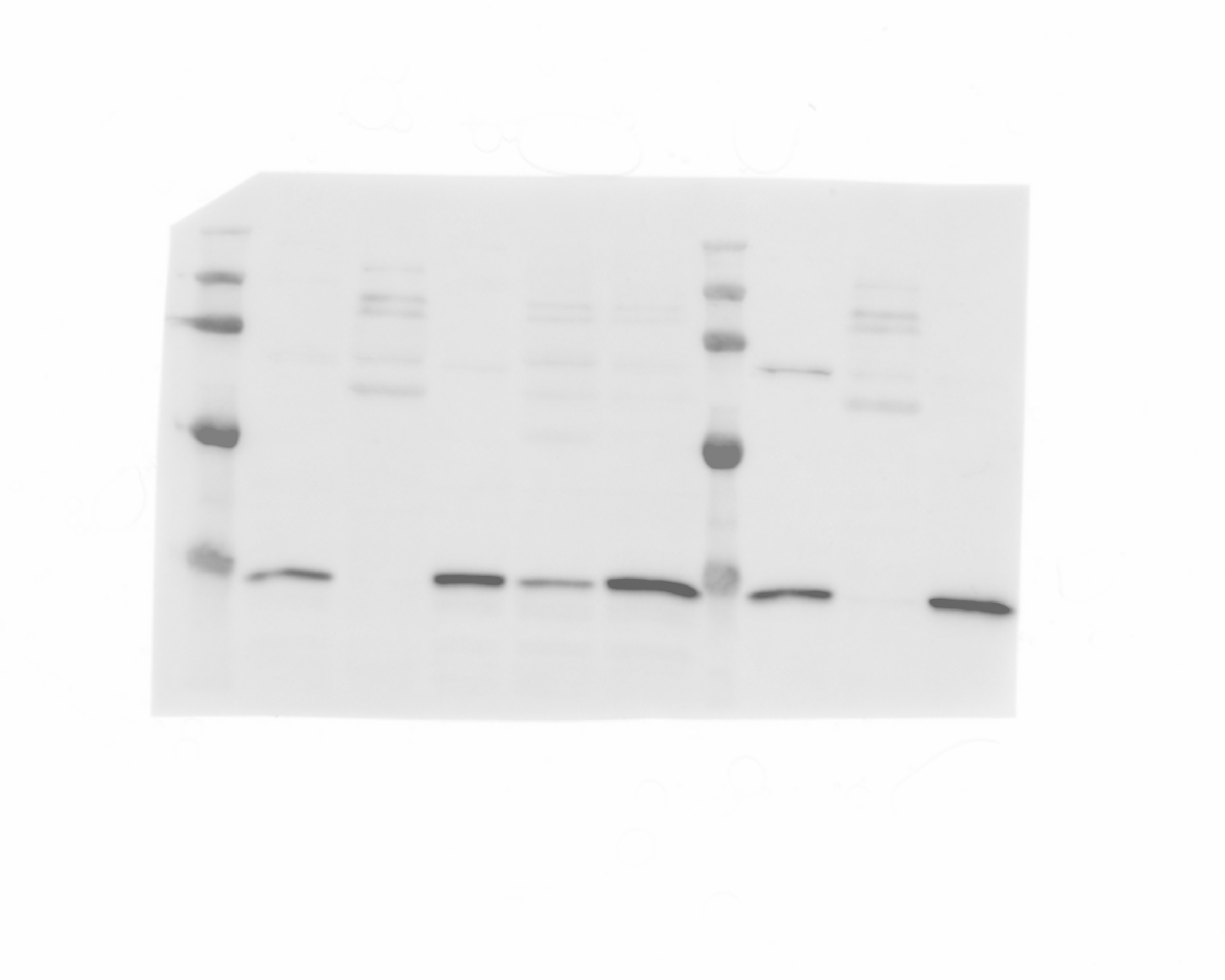

Supplement: Figure 3—source data 1. [file elife-92635-fig3-data1.zip › Figure 3 - source data 1/Figure 3 - source data 3G - RAW/p-cdc2 - 20 sec.tif]

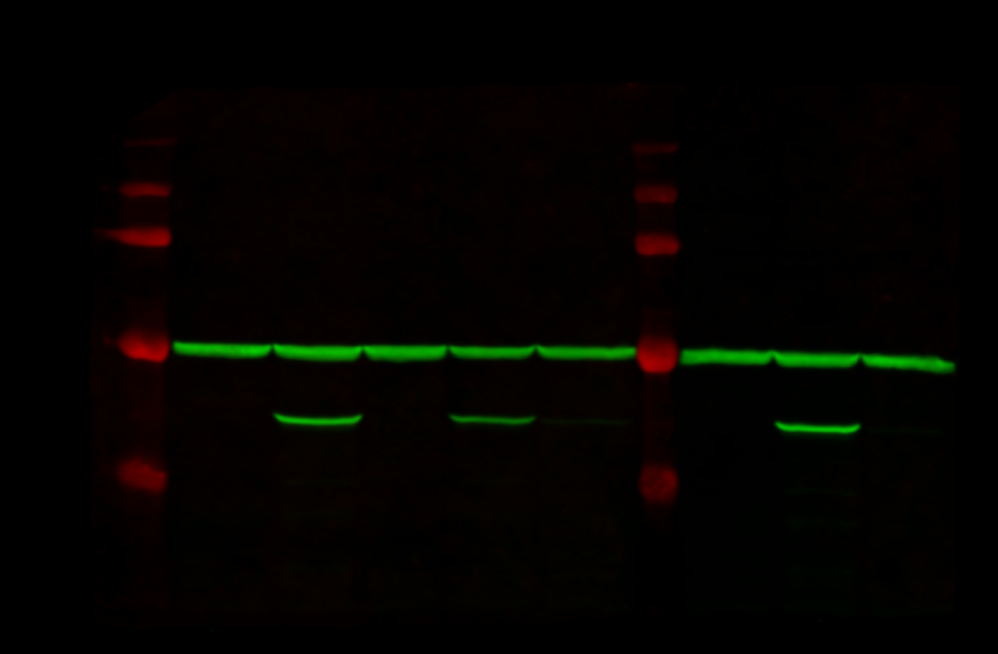

Supplement: Figure 3—source data 1. [file elife-92635-fig3-data1.zip › Figure 3 - source data 1/Figure 3 - source data 3G - RAW/pMAPK and Tubulin M800.tif]

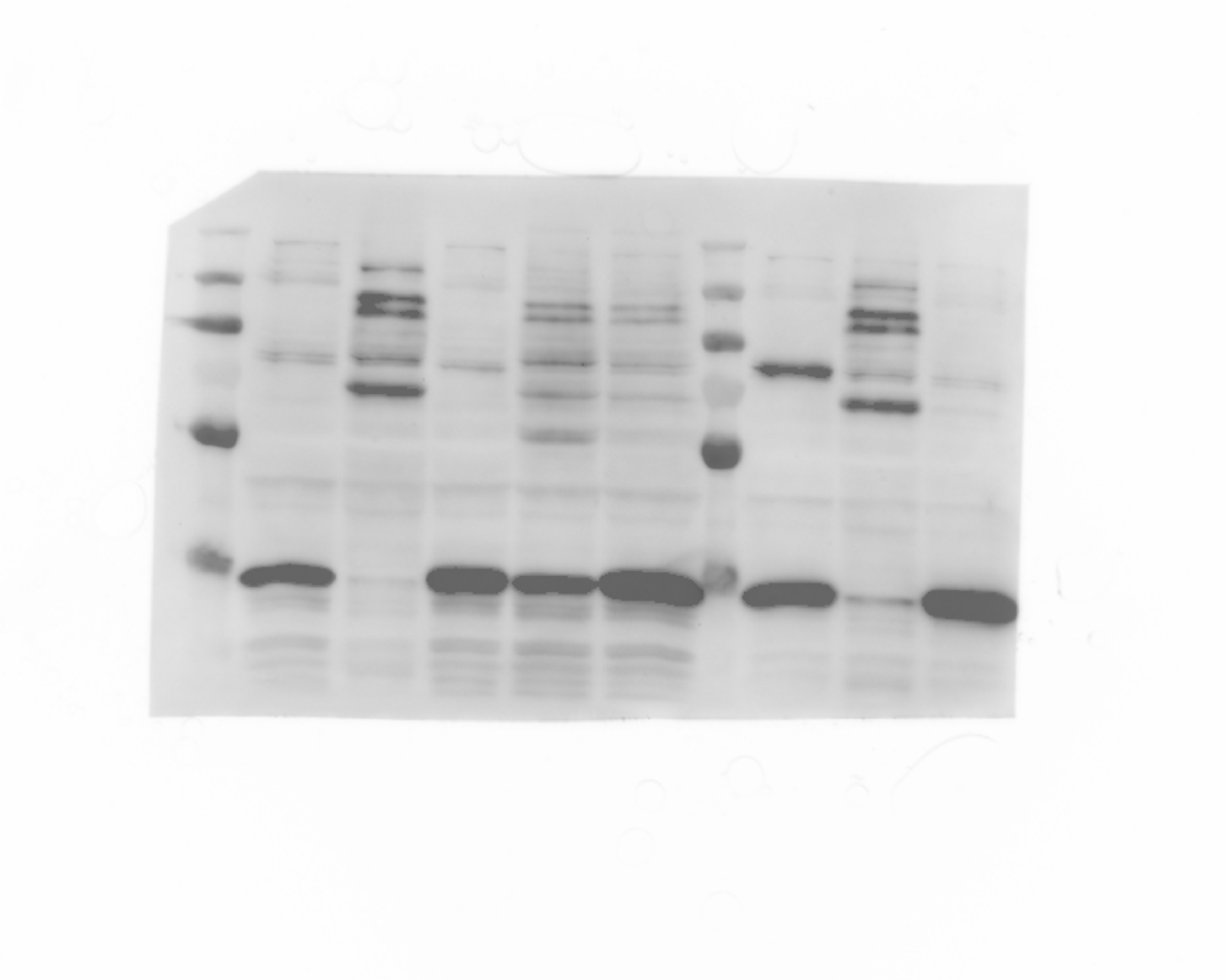

Supplement: Figure 3—source data 1. [file elife-92635-fig3-data1.zip › Figure 3 - source data 1/Figure 3 - source data 3G - RAW/p-plk1 - 1 min.tif]

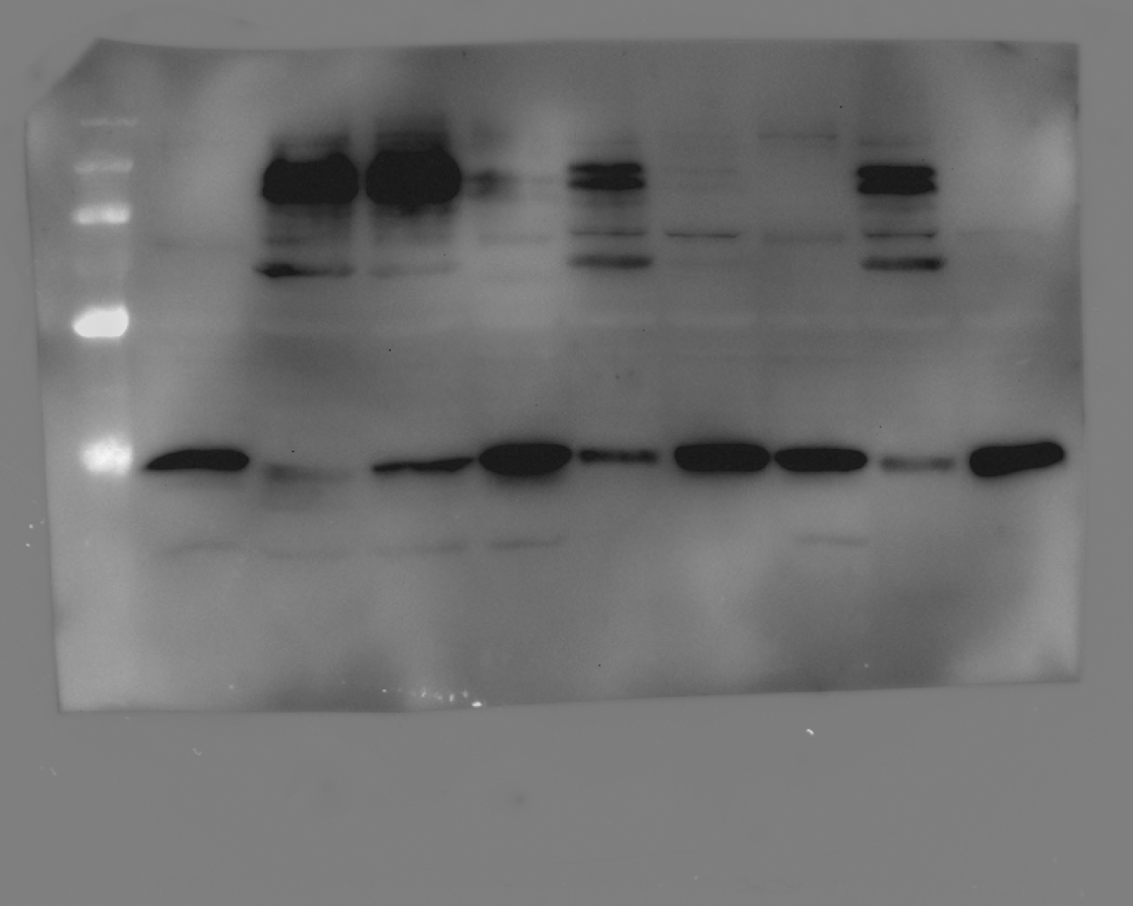

Supplement: Figure 3—source data 1. [file elife-92635-fig3-data1.zip › Figure 3 - source data 1/Figure 3 - source data 3L - RAW/p-cdc2 and p-plk1.tif]

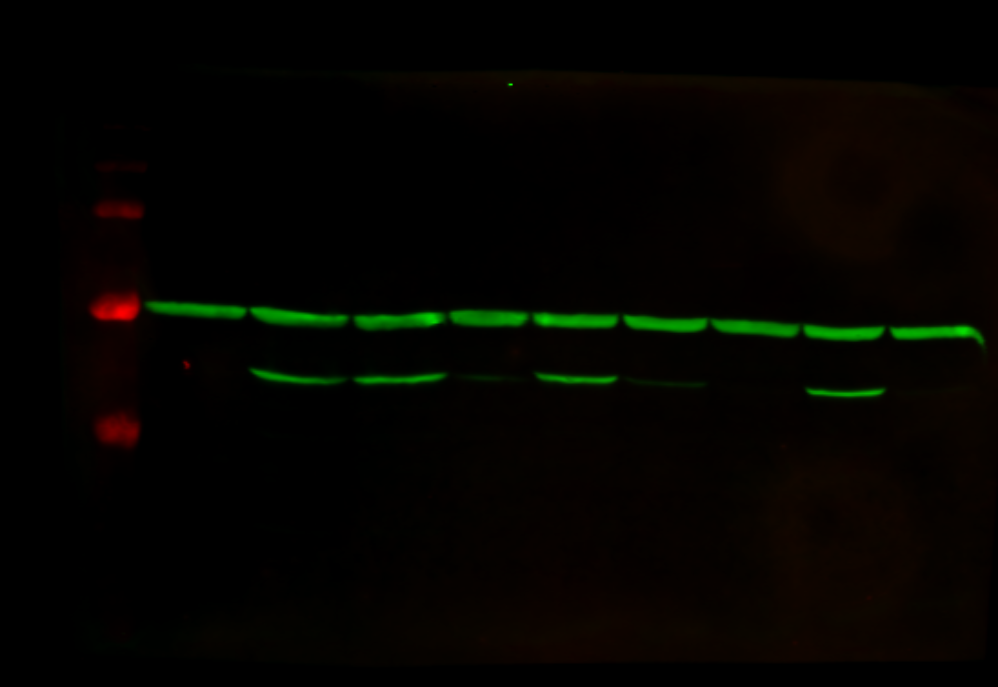

Supplement: Figure 3—source data 1. [file elife-92635-fig3-data1.zip › Figure 3 - source data 1/Figure 3 - source data 3L - RAW/p-MAPK and Tubulin M800.tif]

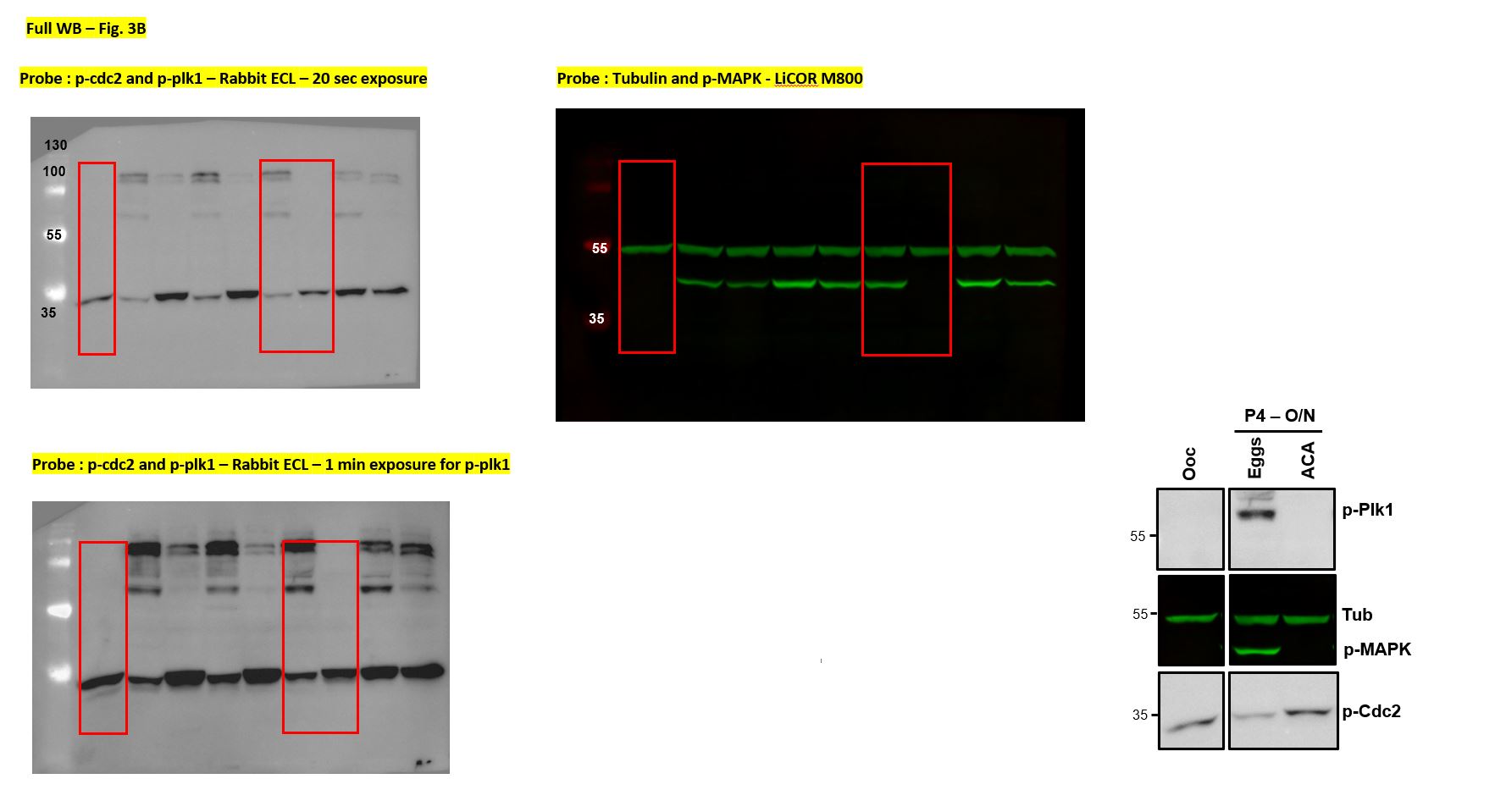

Supplement: Figure 3—source data 2. [file elife-92635-fig3-data2.zip › Figure 3 - source data 2/Figure 3 - source data 3B - Labeled/3B Labeled.JPG]

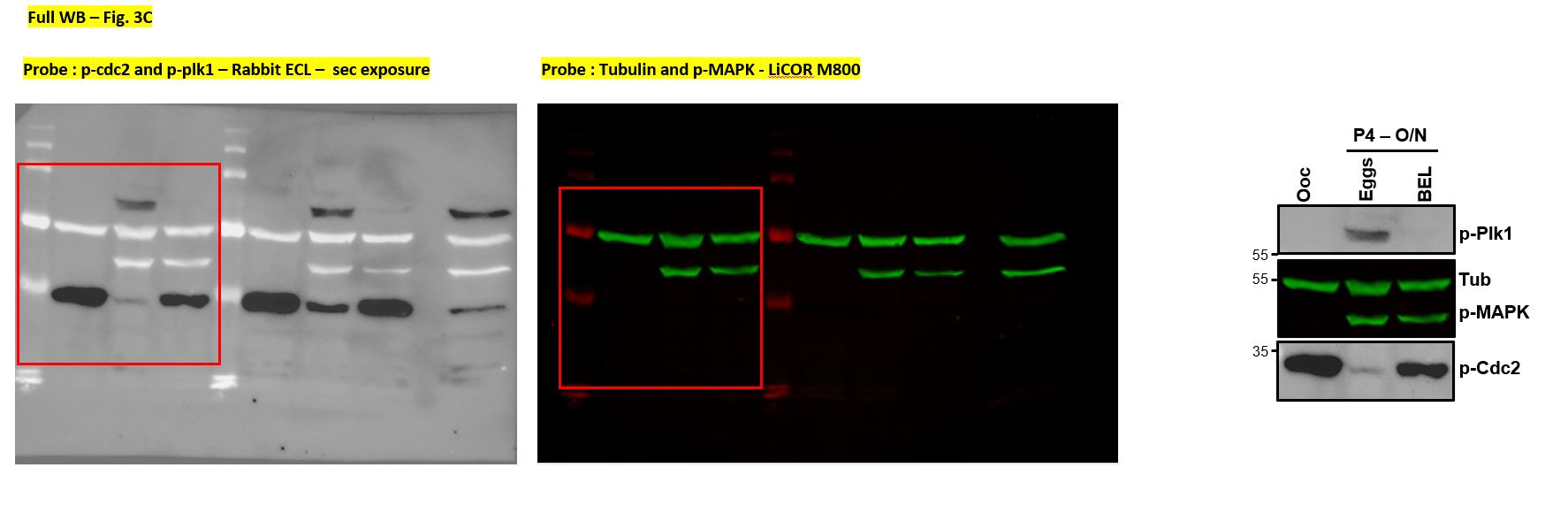

Supplement: Figure 3—source data 2. [file elife-92635-fig3-data2.zip › Figure 3 - source data 2/Figure 3 - source data 3C - Labeled/3C Labeled.JPG]

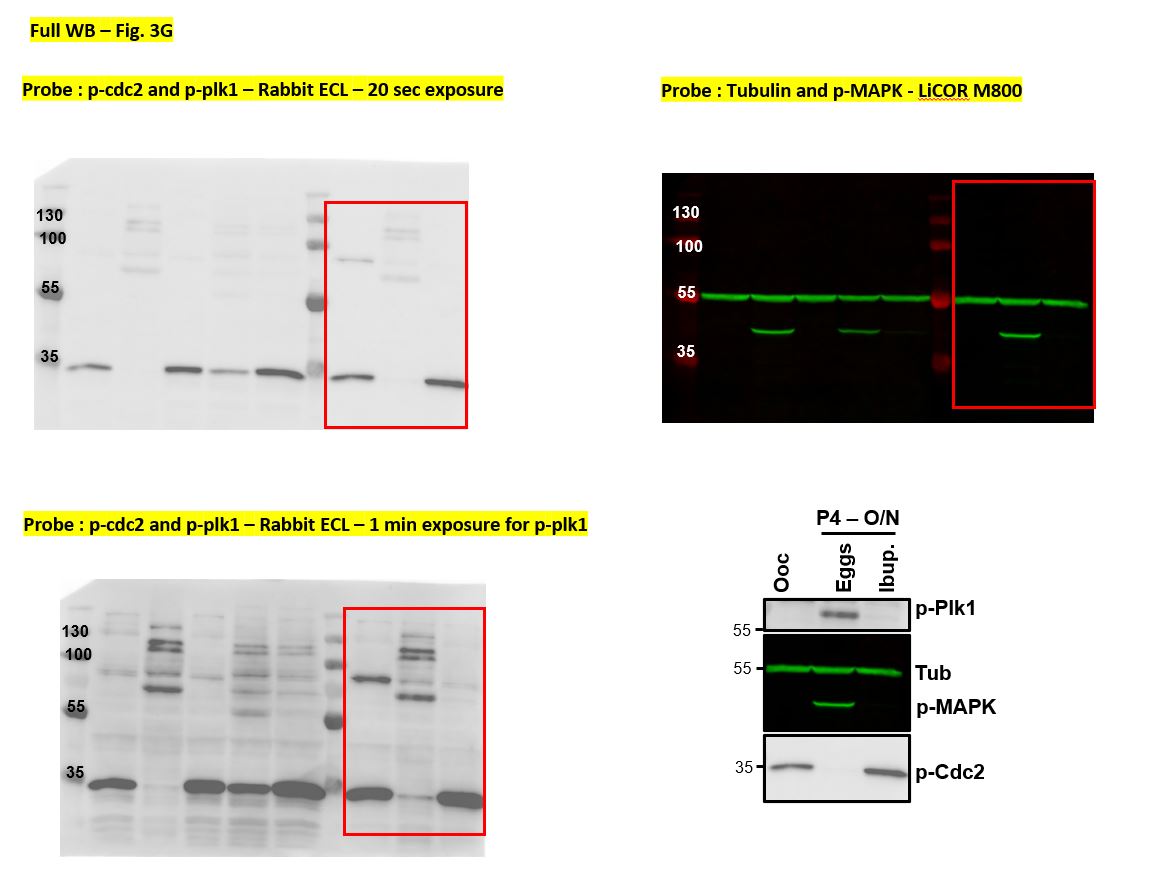

Supplement: Figure 3—source data 2. [file elife-92635-fig3-data2.zip › Figure 3 - source data 2/Figure 3 - source data 3G - Labeled/3G Labeled.JPG]

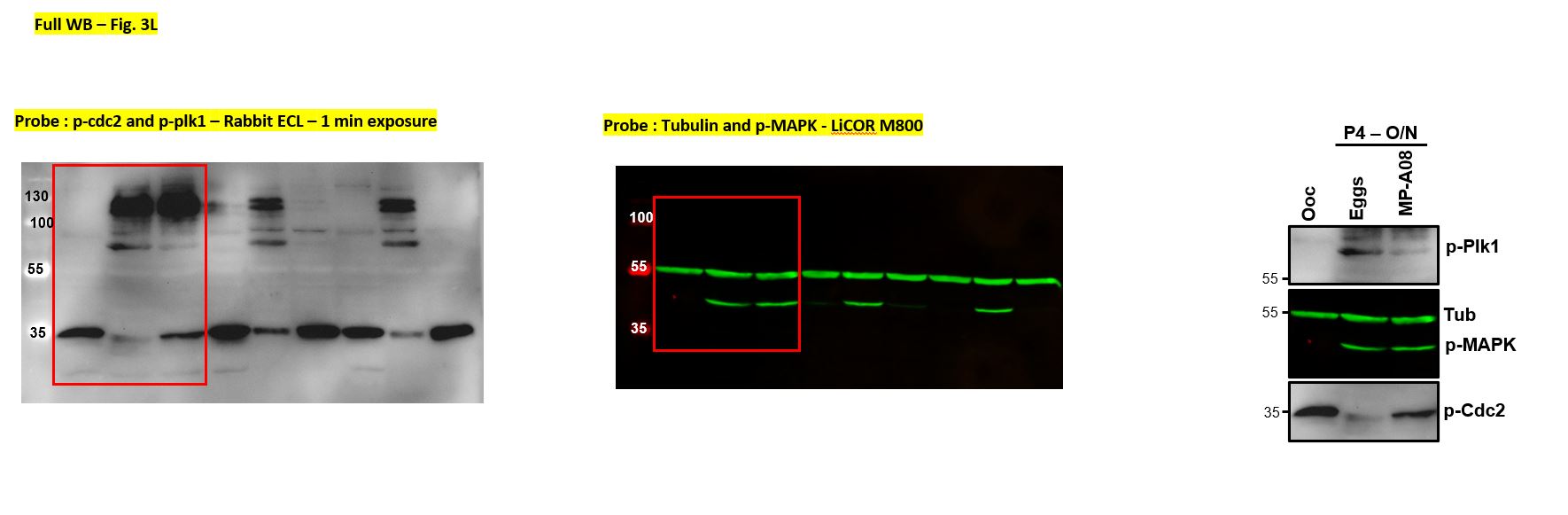

Supplement: Figure 3—source data 2. [file elife-92635-fig3-data2.zip › Figure 3 - source data 2/Figure 3 - source data 3L - Labeled/3L Labeled.JPG]

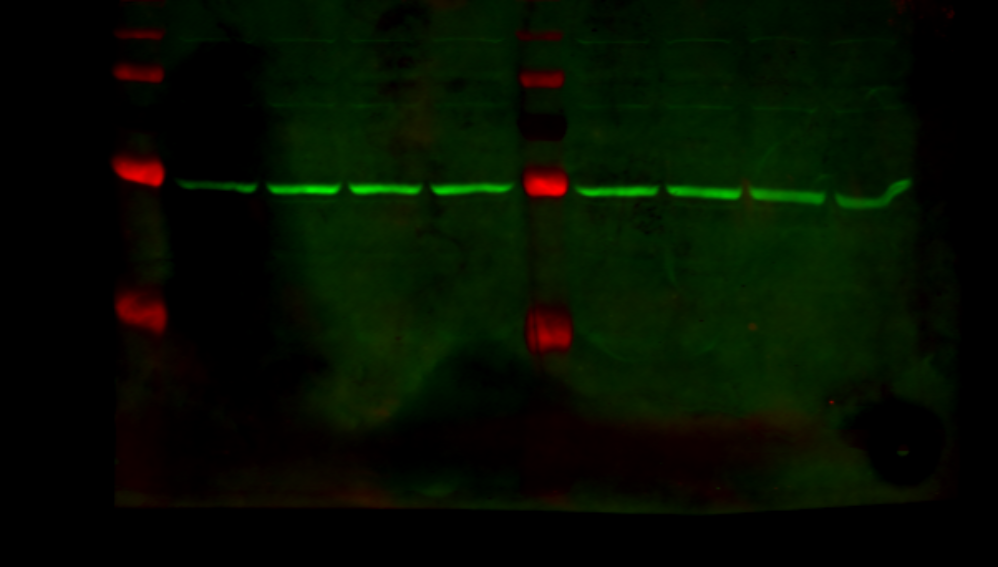

Supplement: Figure 4—source data 1. [file elife-92635-fig4-data1.zip › Figure 4 - source data 1/Figure 4 - source data 4C - RAW/S1PR3 R800.tif]

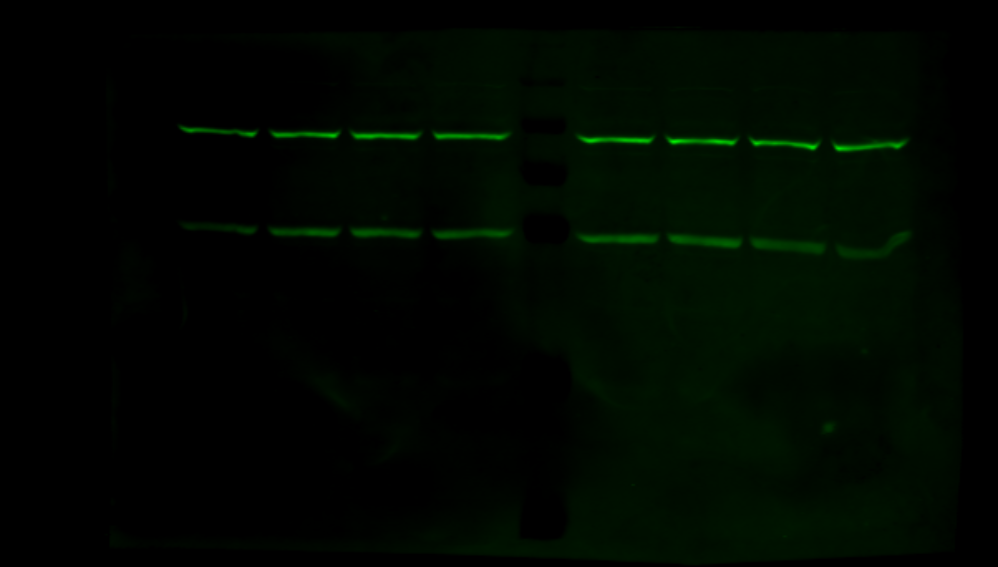

Supplement: Figure 4—source data 1. [file elife-92635-fig4-data1.zip › Figure 4 - source data 1/Figure 4 - source data 4C - RAW/then APPL1 R800.tif]

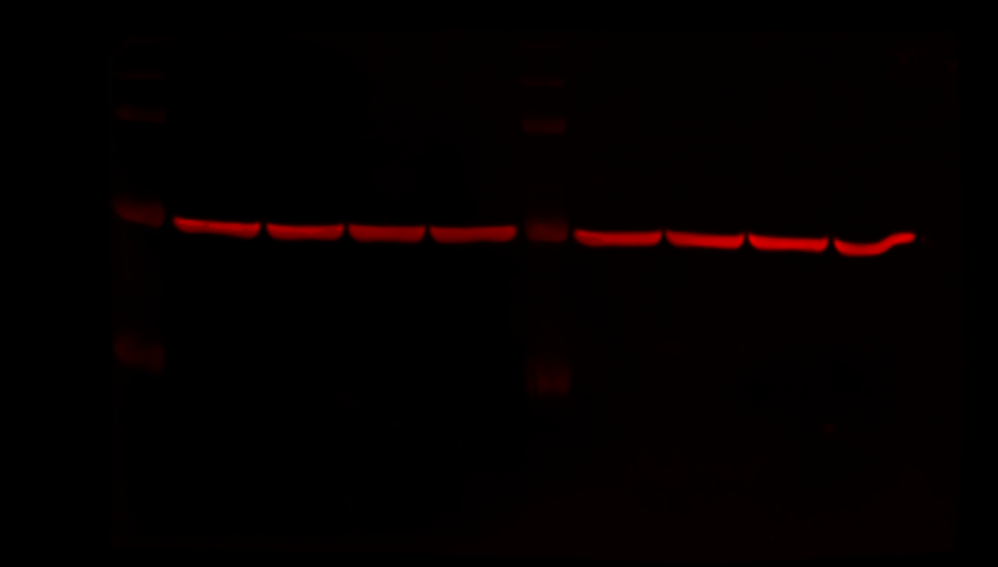

Supplement: Figure 4—source data 1. [file elife-92635-fig4-data1.zip › Figure 4 - source data 1/Figure 4 - source data 4C - RAW/Tubulin M680.tif]

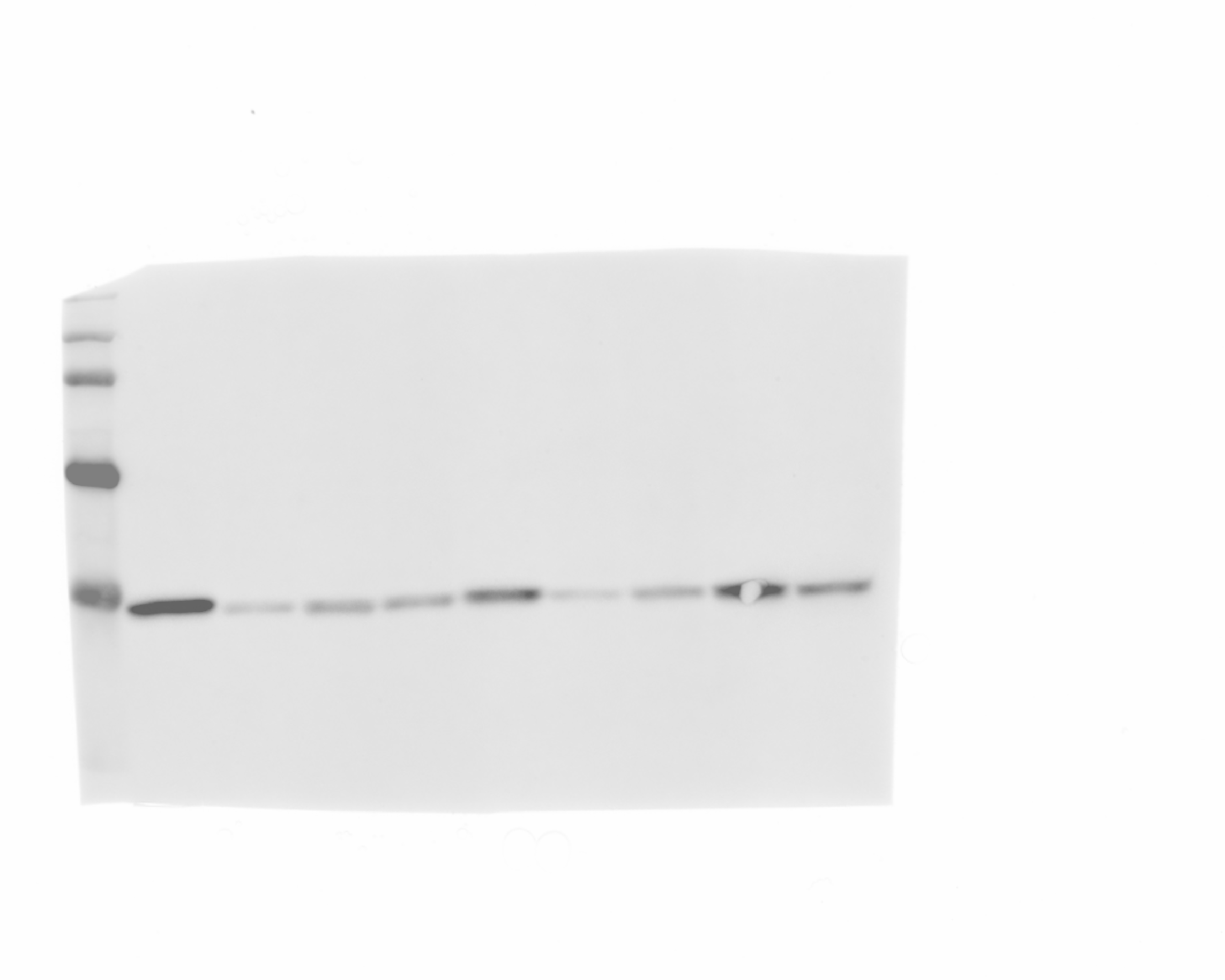

Supplement: Figure 4—source data 1. [file elife-92635-fig4-data1.zip › Figure 4 - source data 1/Figure 4 - source data 4E - RAW/p-cdc2.tif]

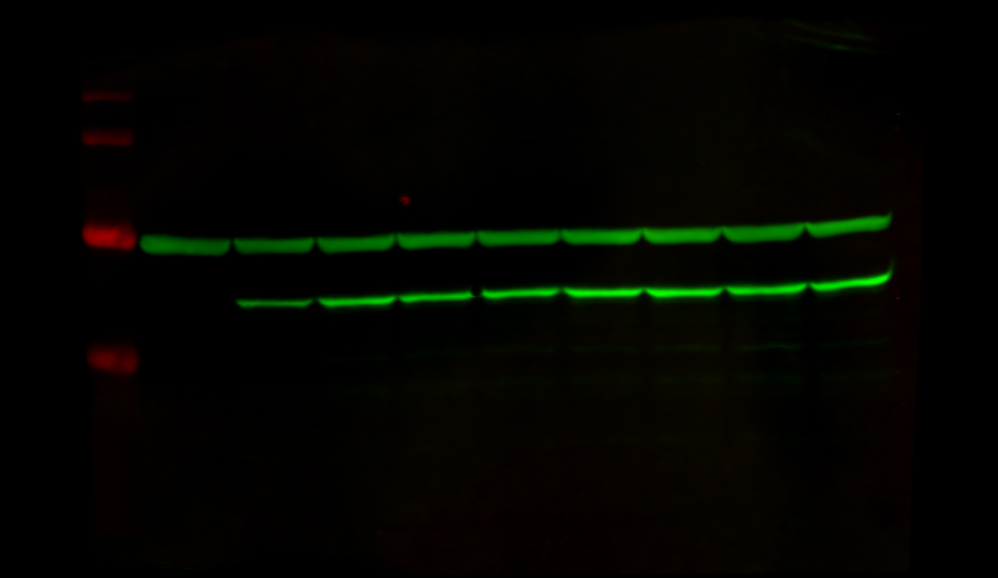

Supplement: Figure 4—source data 1. [file elife-92635-fig4-data1.zip › Figure 4 - source data 1/Figure 4 - source data 4E - RAW/p-MAPK and Tubulin M800.tif]

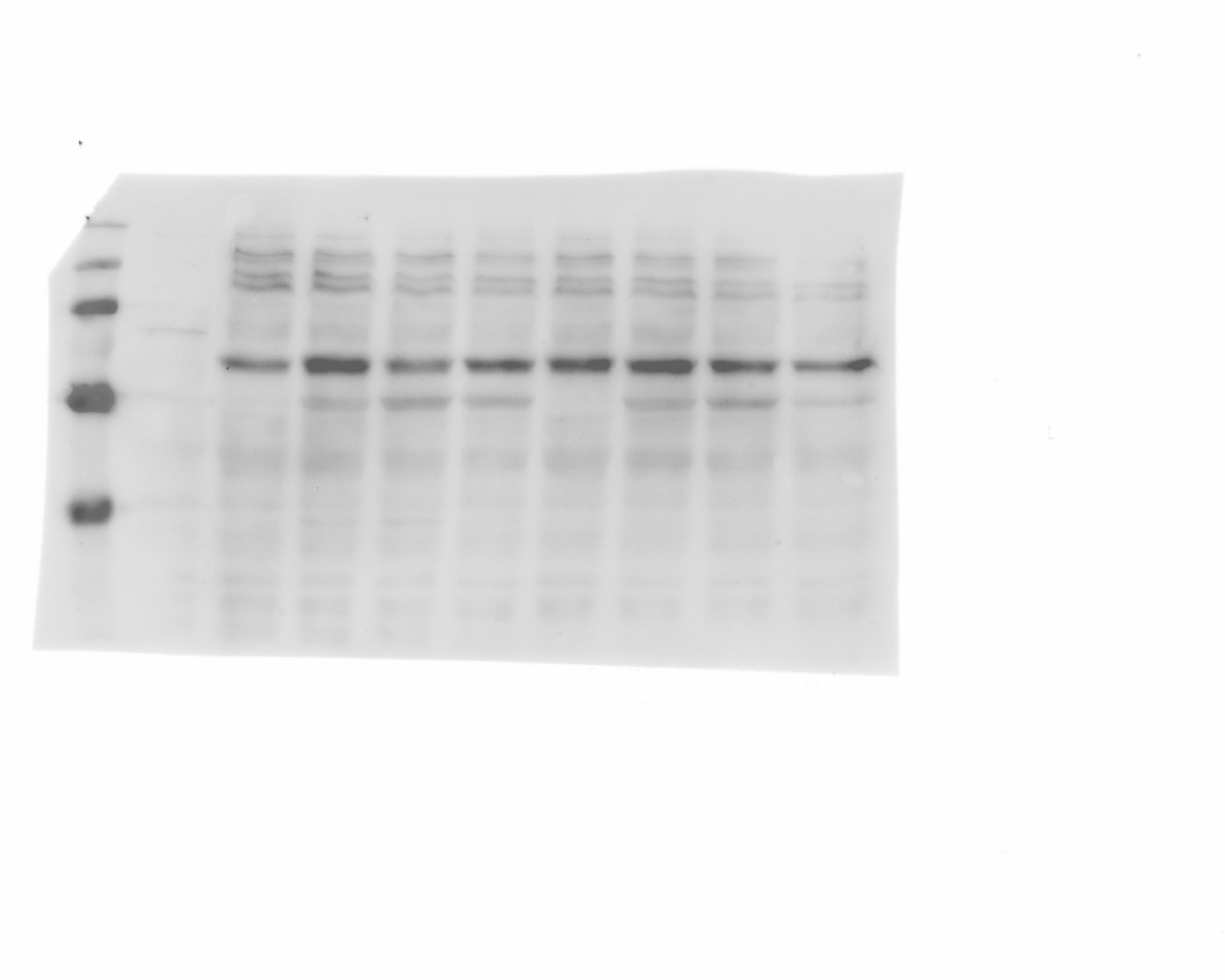

Supplement: Figure 4—source data 1. [file elife-92635-fig4-data1.zip › Figure 4 - source data 1/Figure 4 - source data 4E - RAW/p-plk1.tif]

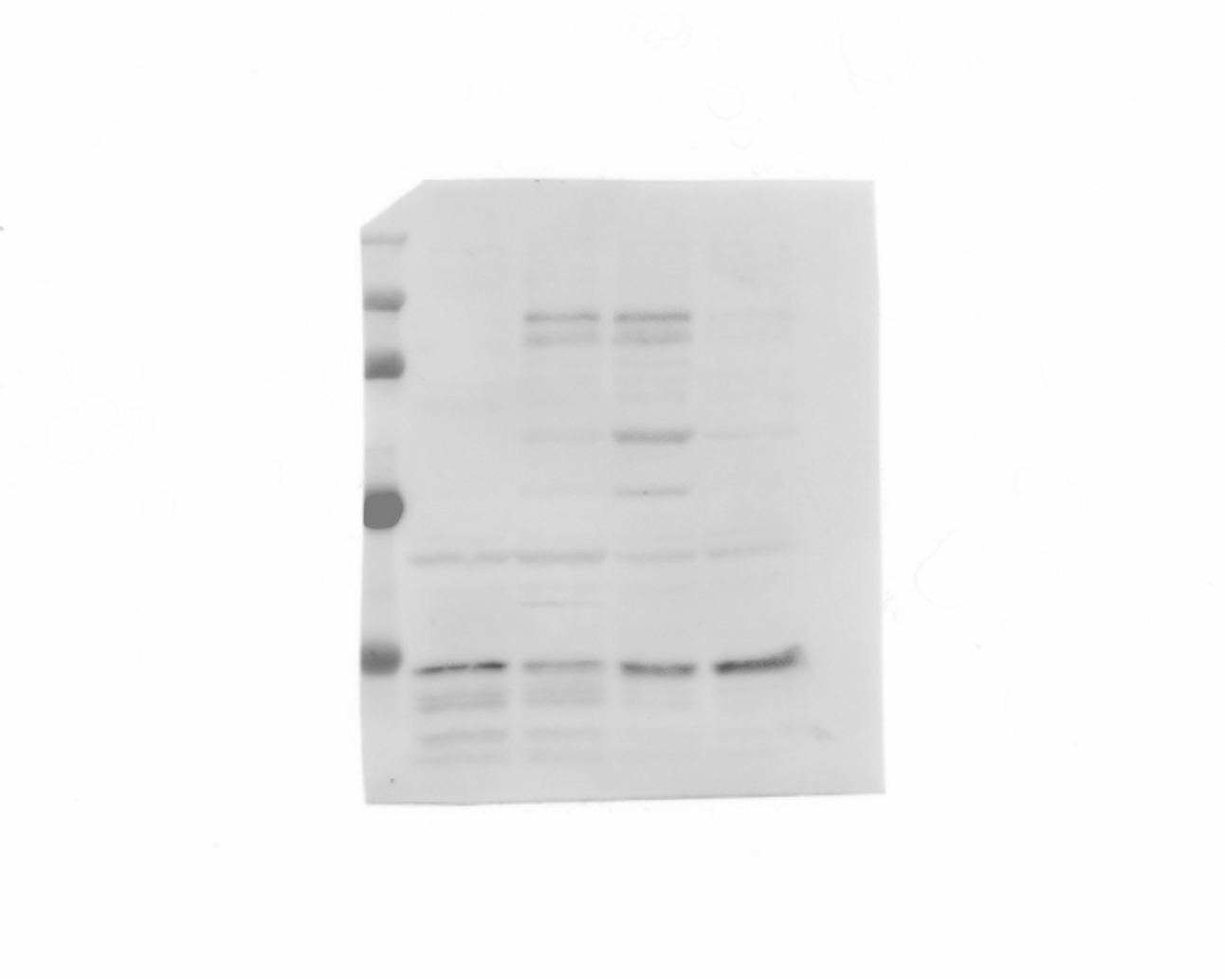

Supplement: Figure 4—source data 1. [file elife-92635-fig4-data1.zip › Figure 4 - source data 1/Figure 4 - source data 4F - RAW/p-cdc2 20 sec.tif]

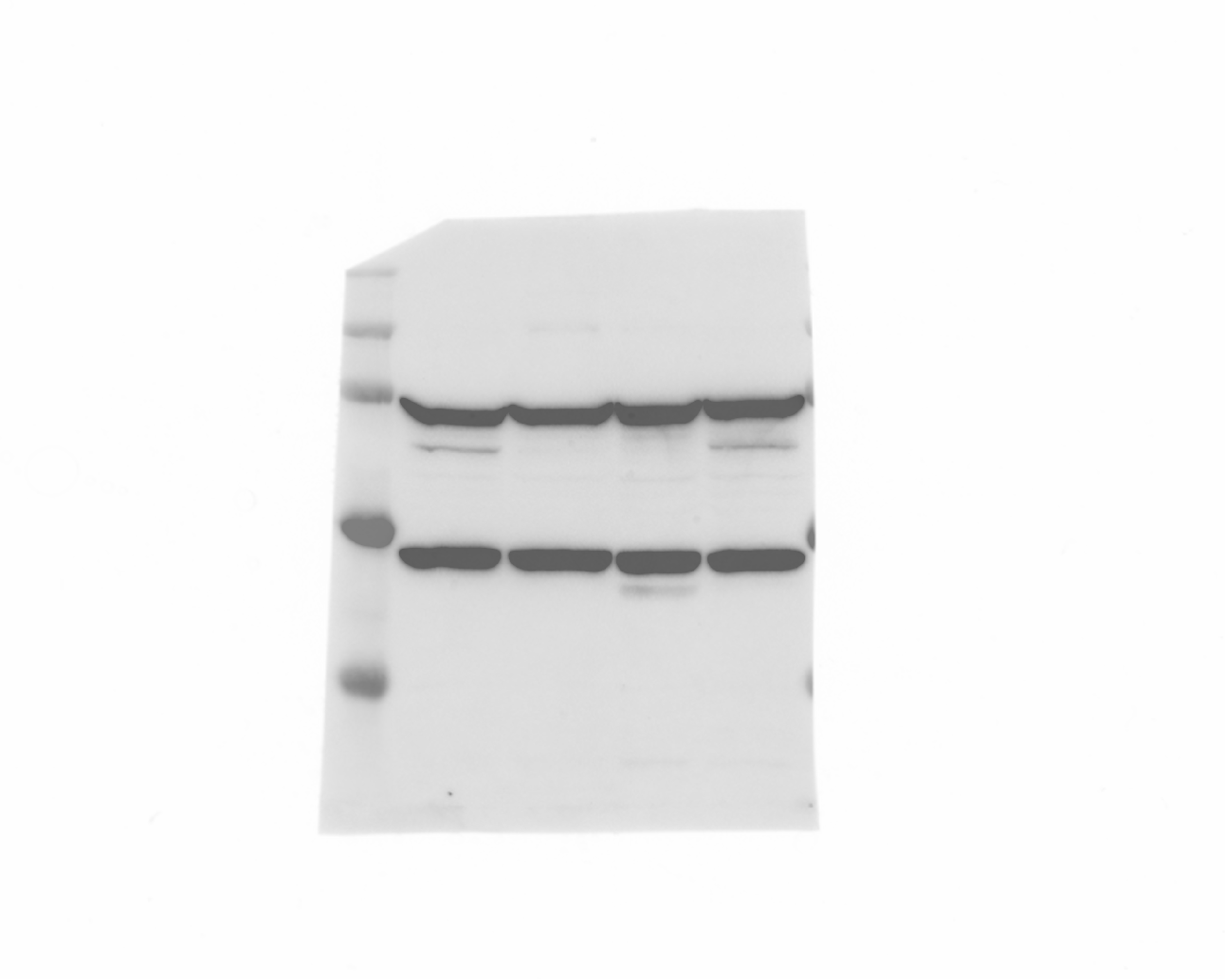

Supplement: Figure 4—source data 1. [file elife-92635-fig4-data1.zip › Figure 4 - source data 1/Figure 4 - source data 4F - RAW/p-cdc25 S216.tif]

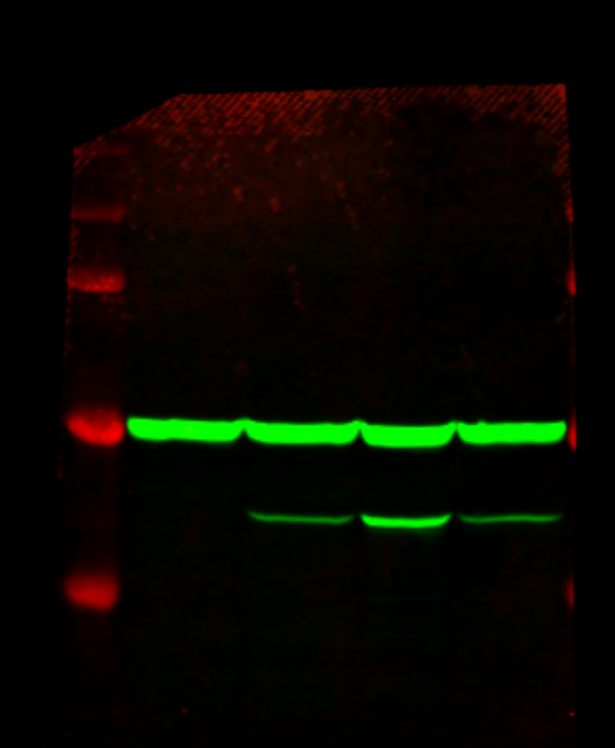

Supplement: Figure 4—source data 1. [file elife-92635-fig4-data1.zip › Figure 4 - source data 1/Figure 4 - source data 4F - RAW/p-MAPK and Tubulin M800.tif]

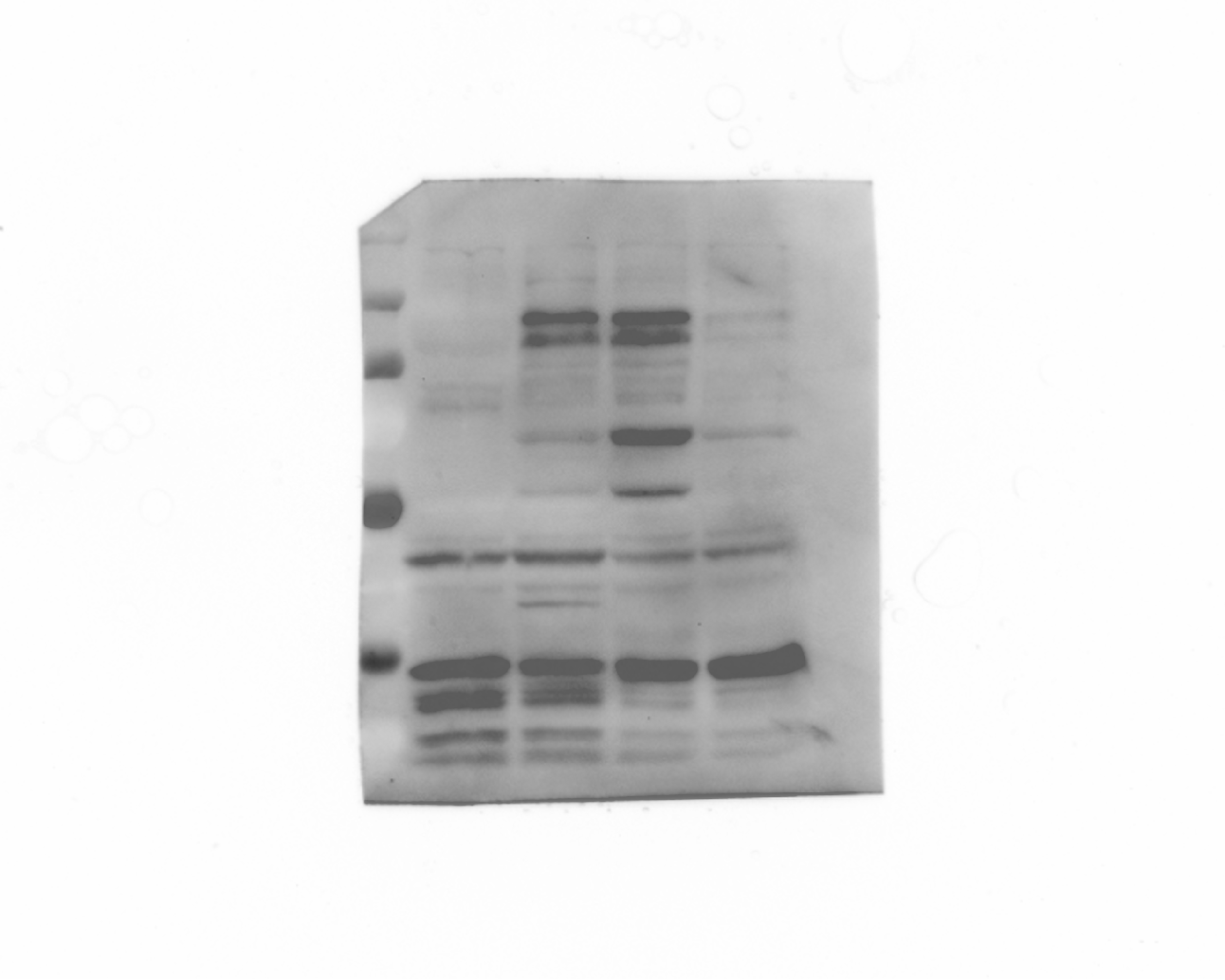

Supplement: Figure 4—source data 1. [file elife-92635-fig4-data1.zip › Figure 4 - source data 1/Figure 4 - source data 4F - RAW/p-plk1 1 min.tif]

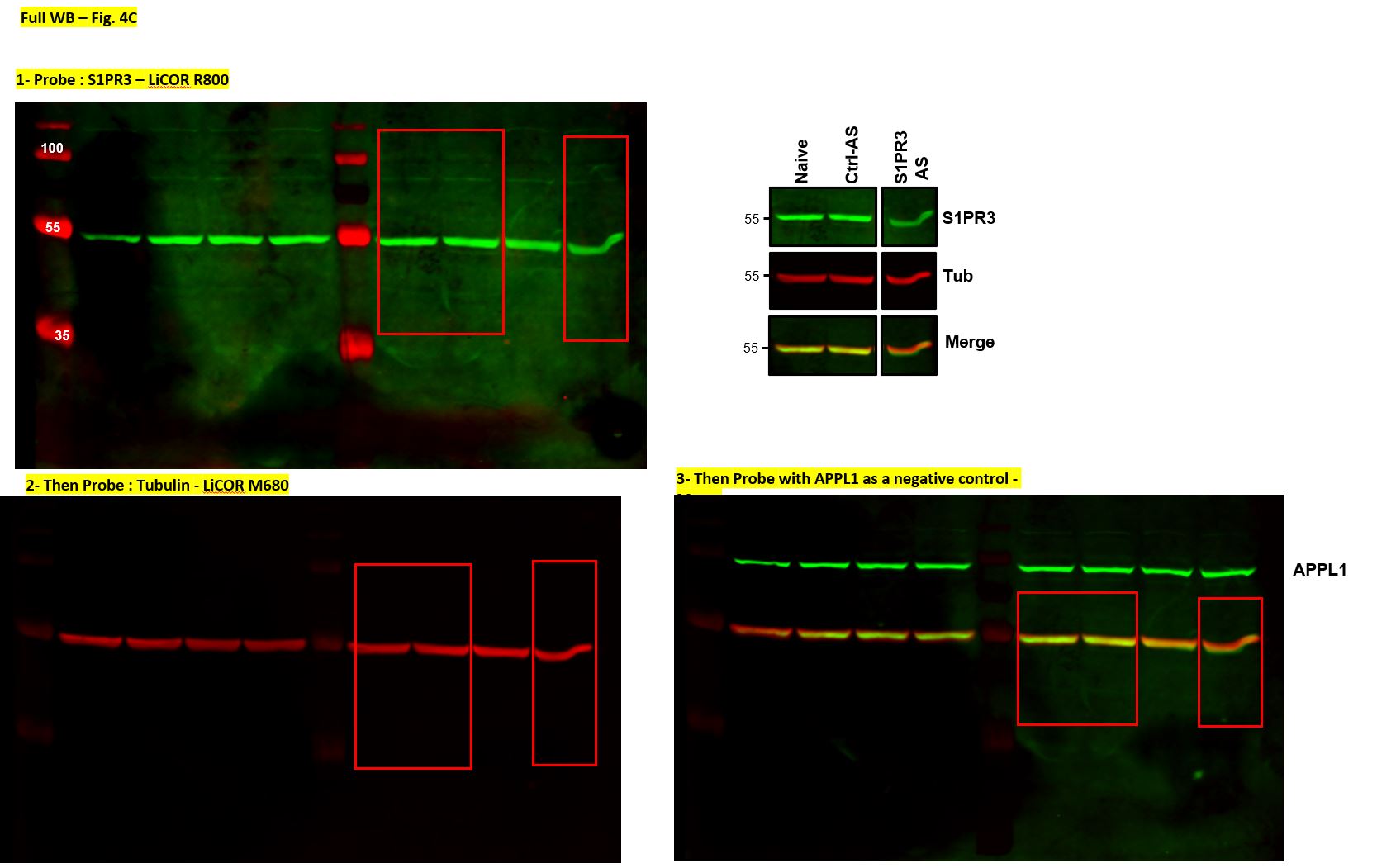

Supplement: Figure 4—source data 2. [file elife-92635-fig4-data2.zip › Figure 4 - source data 2/Figure 4 - source data 4C - Labeled/4C Labeled.JPG]

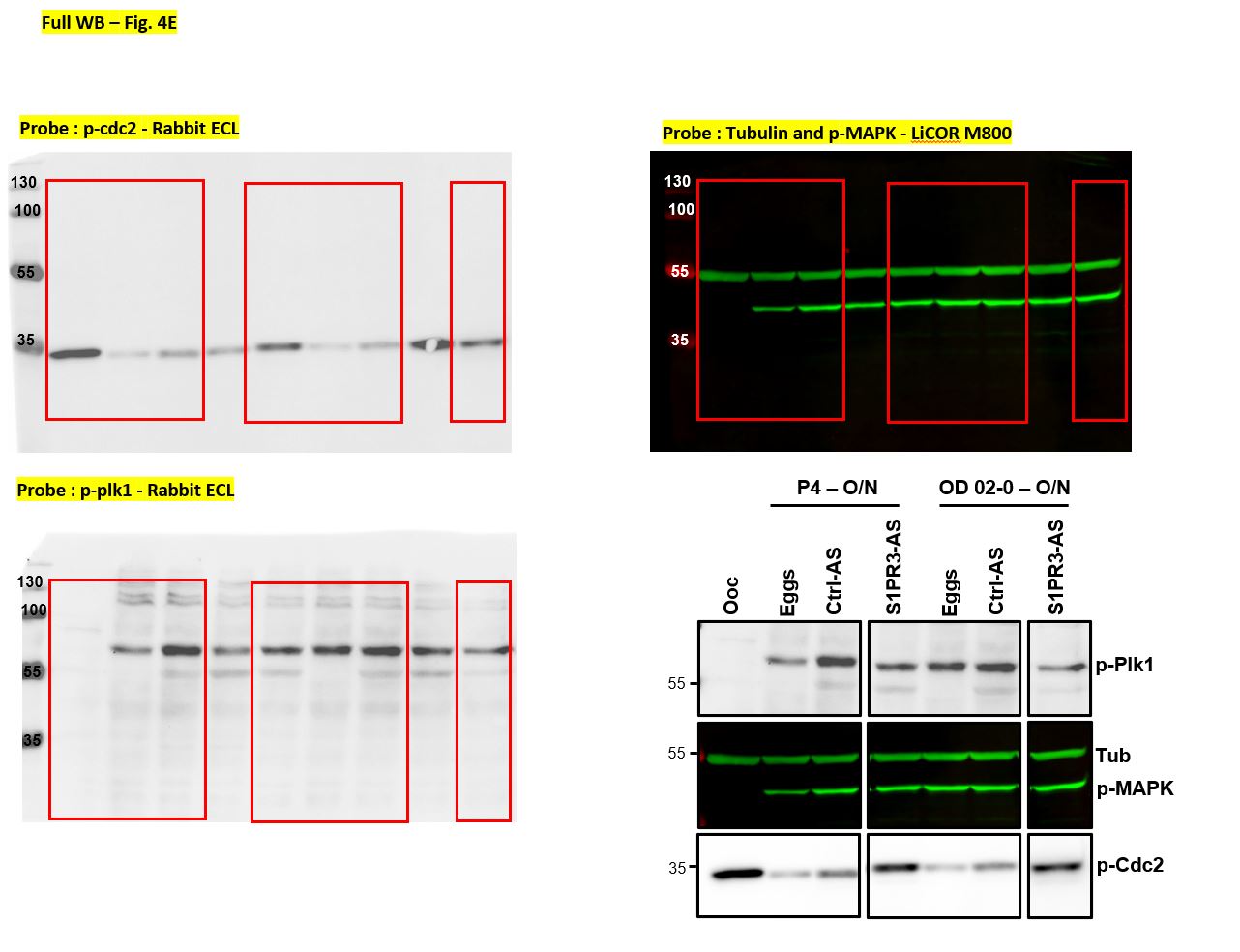

Supplement: Figure 4—source data 2. [file elife-92635-fig4-data2.zip › Figure 4 - source data 2/Figure 4 - source data 4E - Labeled/4E Labeled.JPG]

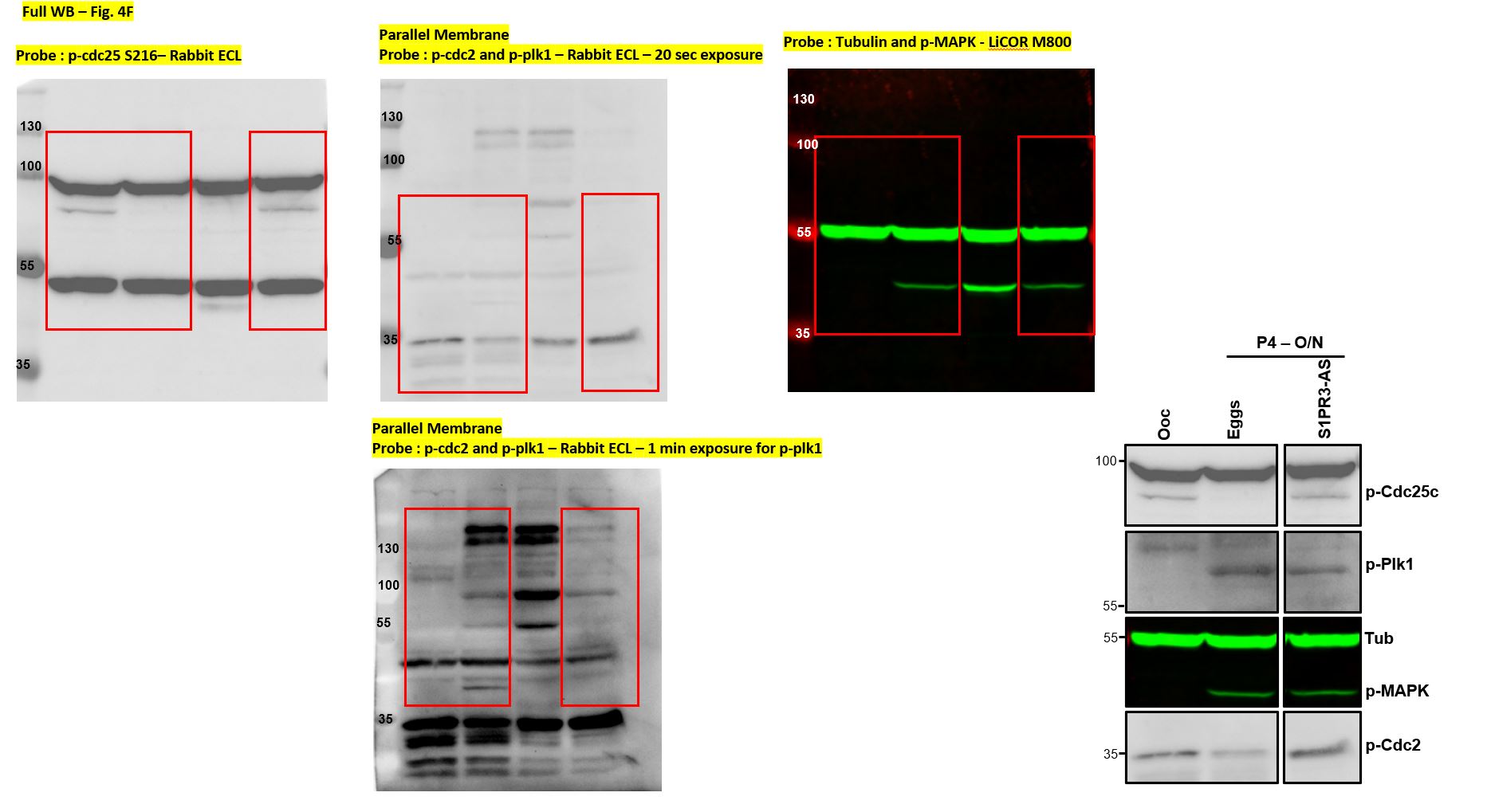

Supplement: Figure 4—source data 2. [file elife-92635-fig4-data2.zip › Figure 4 - source data 2/Figure 4 - source data 4F - Labeled/4F Labeled.JPG]

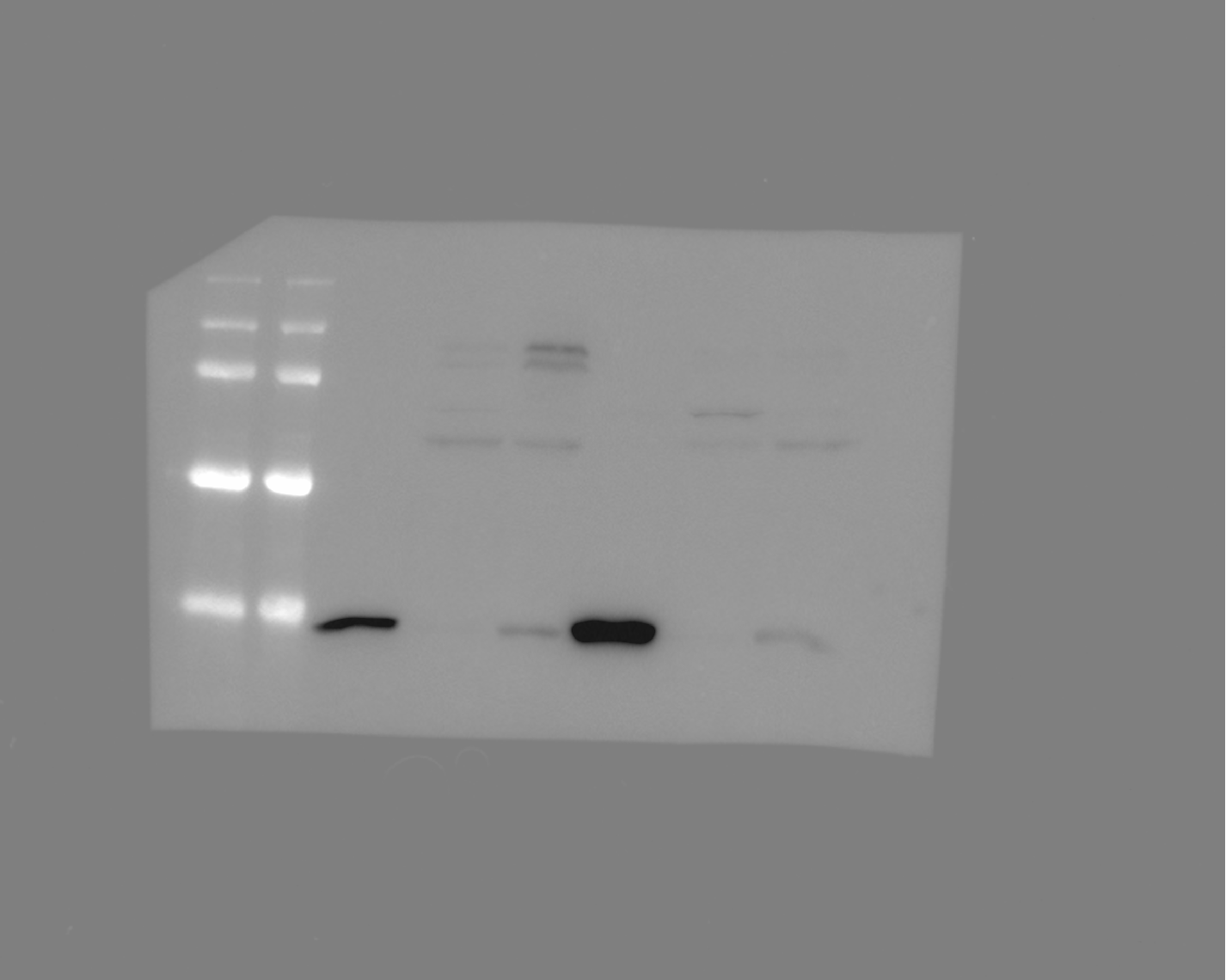

Supplement: Figure 4—figure supplement 1—source data 1. [file elife-92635-fig4-figsupp1-data1.zip › Figure 4 - Figure supplement 4 - source data 1/Figure 4 - Figure supplement 4D - RAW/p-cdc2 - 20 sec.tif]

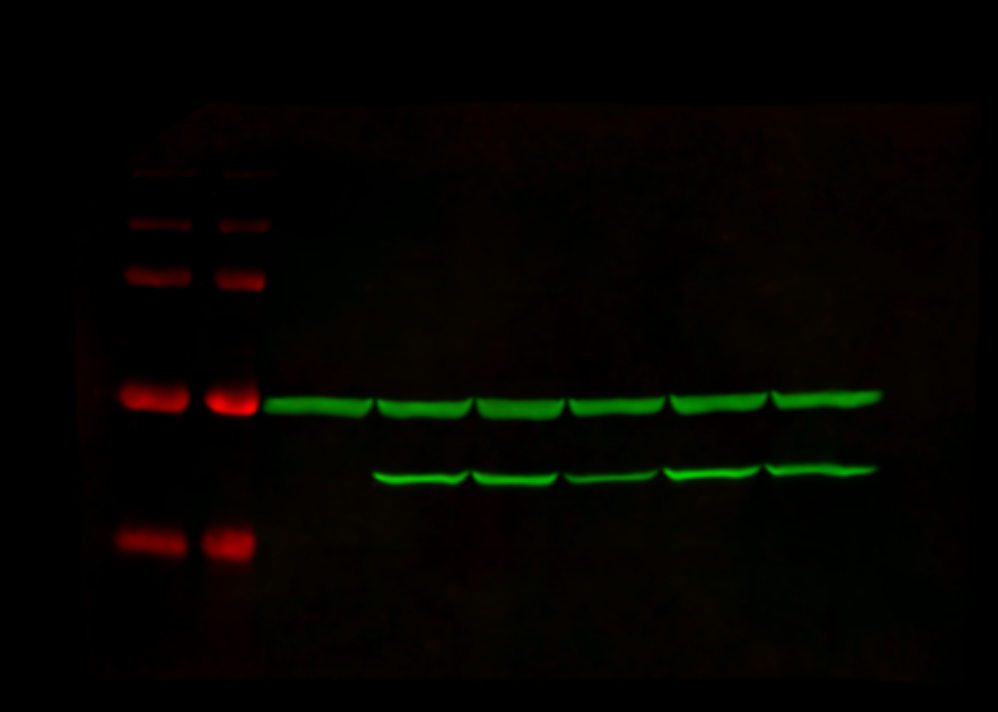

Supplement: Figure 4—figure supplement 1—source data 1. [file elife-92635-fig4-figsupp1-data1.zip › Figure 4 - Figure supplement 4 - source data 1/Figure 4 - Figure supplement 4D - RAW/p-MAPK and Tubulin M800.tif]

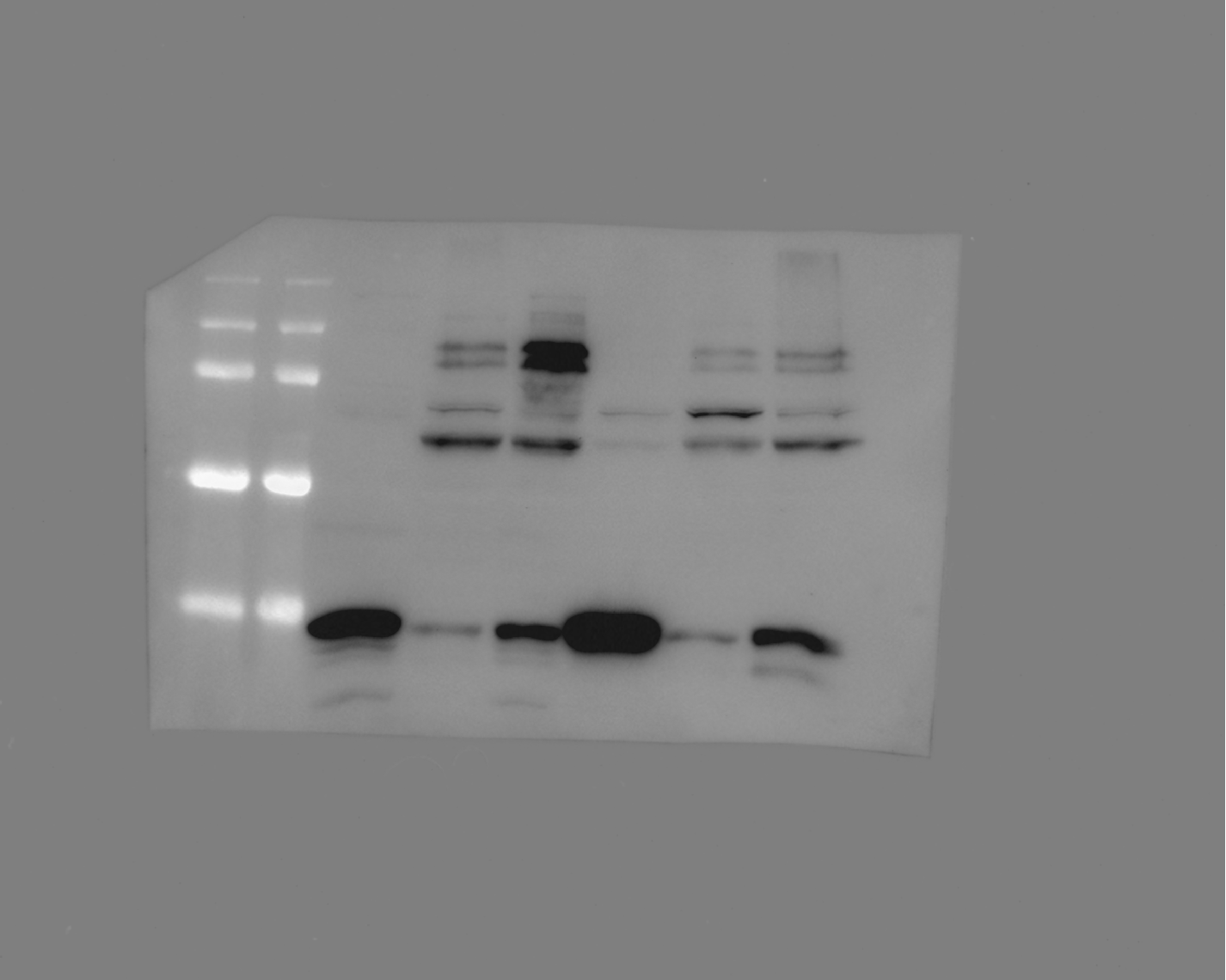

Supplement: Figure 4—figure supplement 1—source data 1. [file elife-92635-fig4-figsupp1-data1.zip › Figure 4 - Figure supplement 4 - source data 1/Figure 4 - Figure supplement 4D - RAW/p-plk1 - 1 min.tif]

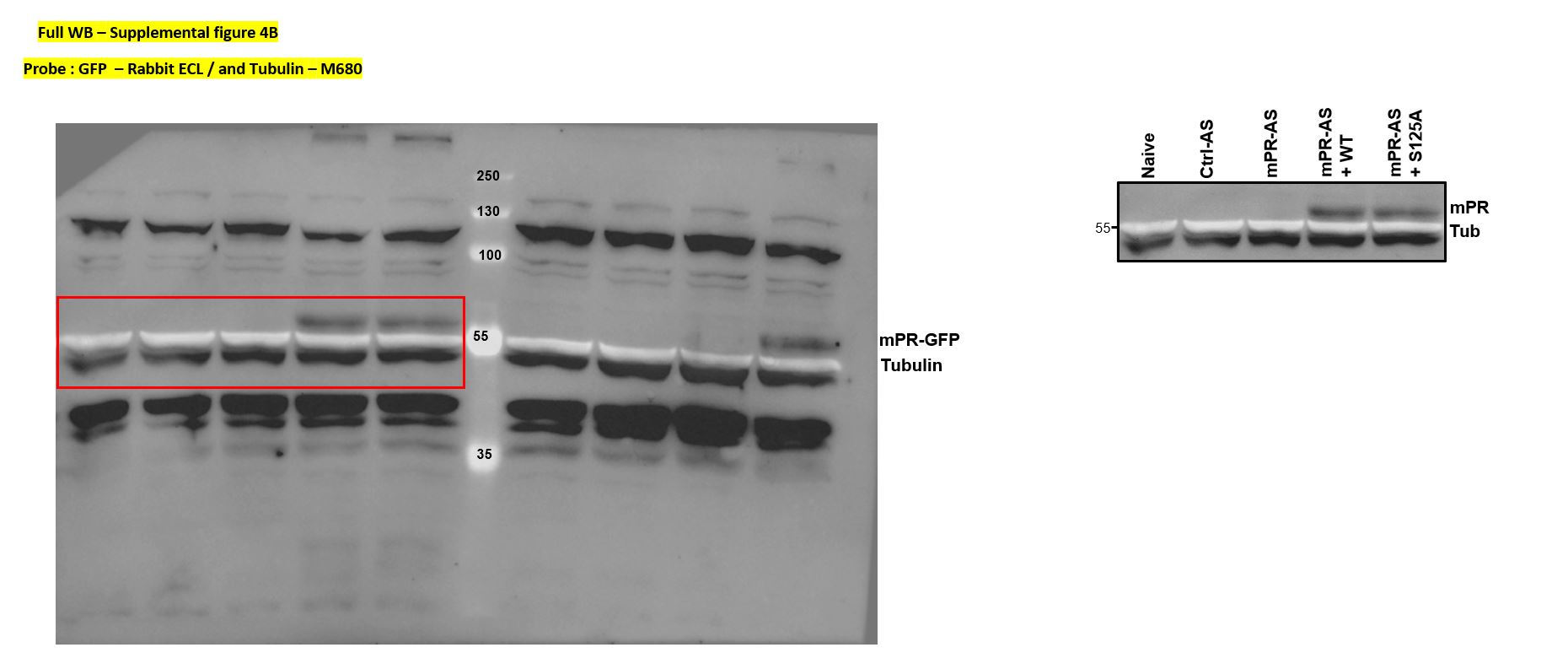

Supplement: Figure 4—figure supplement 1—source data 2. [file elife-92635-fig4-figsupp1-data2.zip › Figure 4 - Figure supplement 4 - source data 2/Figure 4 - Figure supplement 4B - Labeled/Figure 4 - source data Figure supplement 4B Labeled.JPG]

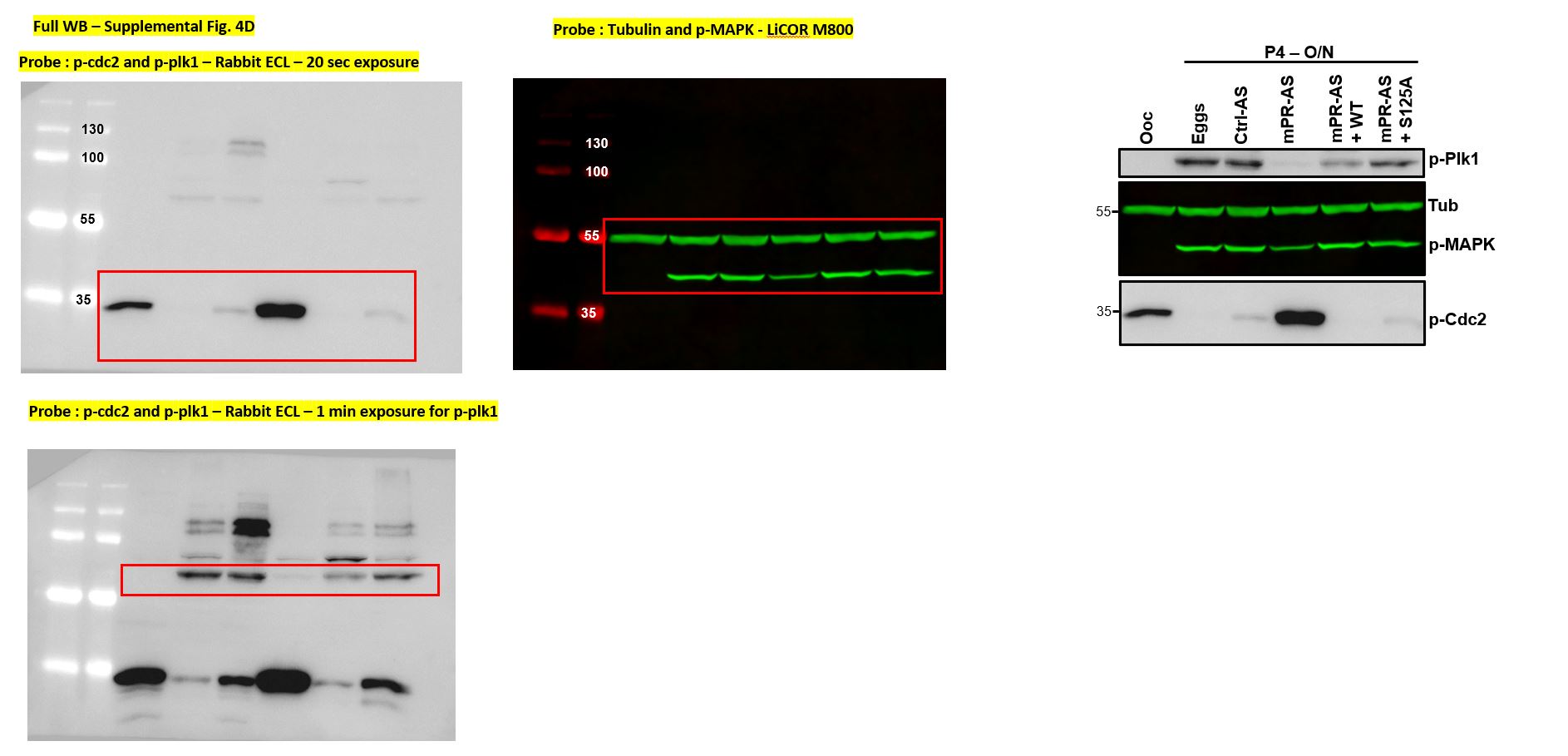

Supplement: Figure 4—figure supplement 1—source data 2. [file elife-92635-fig4-figsupp1-data2.zip › Figure 4 - Figure supplement 4 - source data 2/Figure 4 - Figure supplement 4D - Labeled/Figure 4 - source data Figure supplement 4D Labeled.JPG]

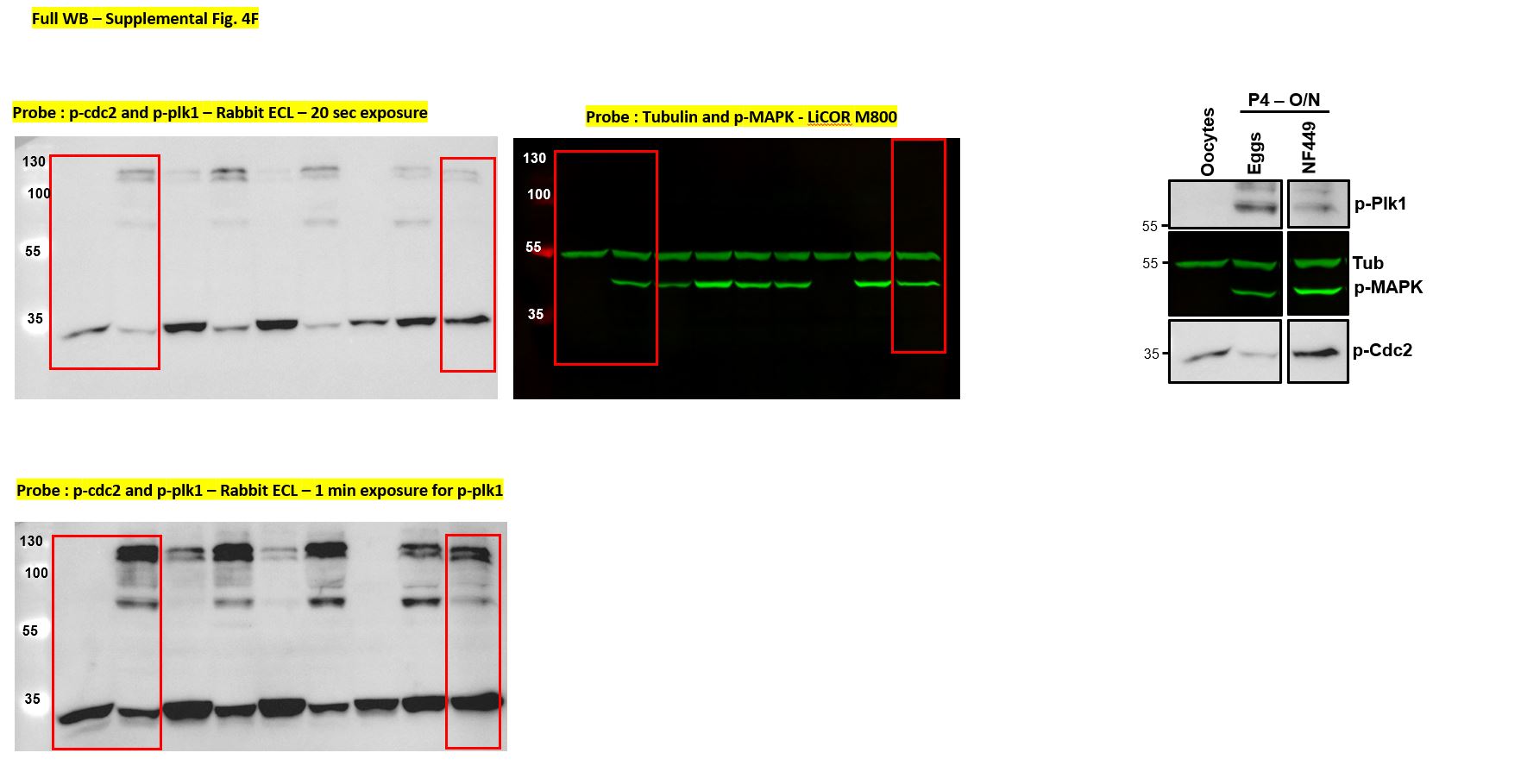

Supplement: Figure 4—figure supplement 1—source data 2. [file elife-92635-fig4-figsupp1-data2.zip › Figure 4 - Figure supplement 4 - source data 2/Figure 4 - Figure supplement 4F - Labeled/Figure 4 - source data Figure supplement 4F Labeled.JPG]

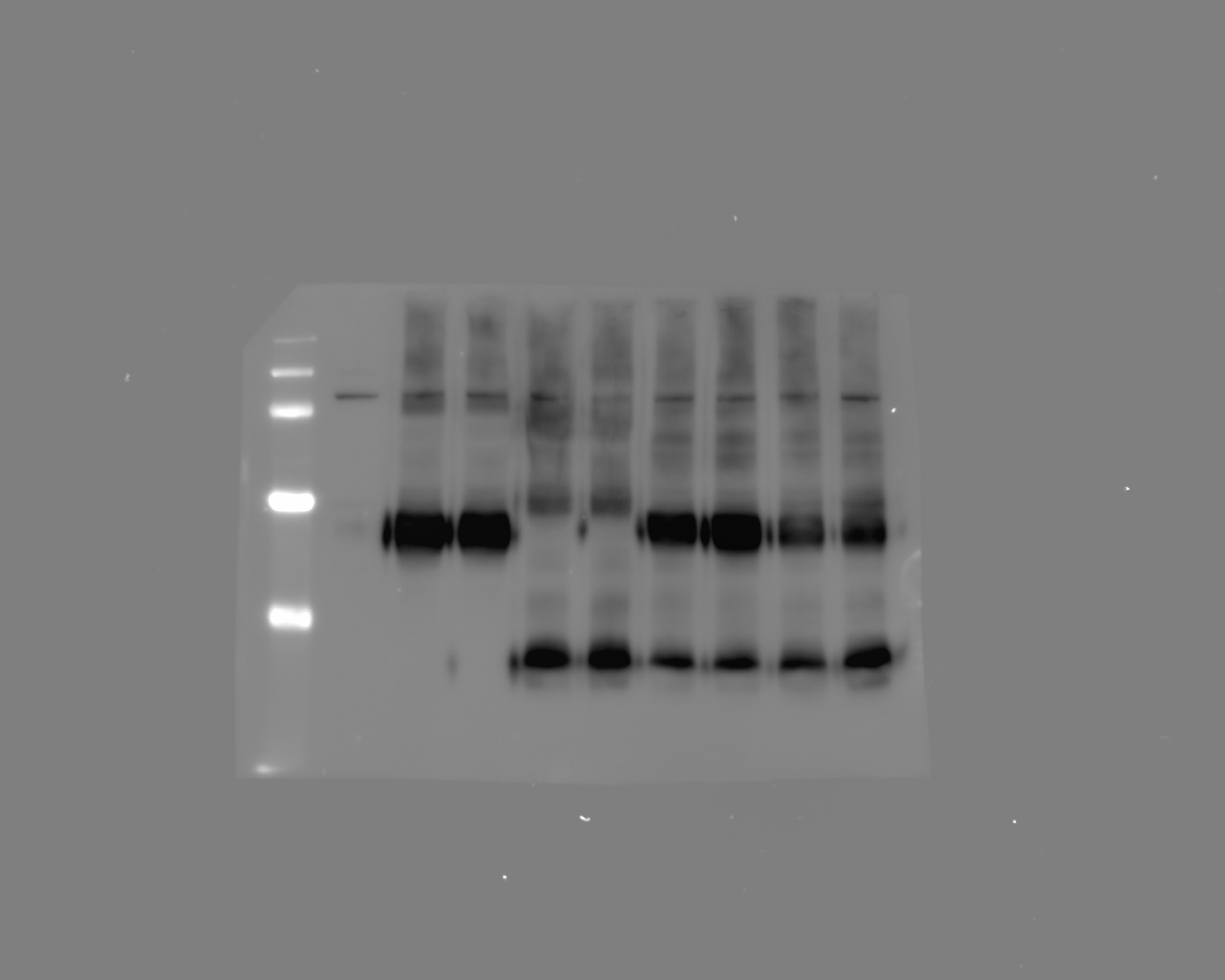

Supplement: Figure 5—source data 1. [file elife-92635-fig5-data1.zip › Figure 5 - source data 1/Figure 5 - source data 5C - RAW/His.tif]

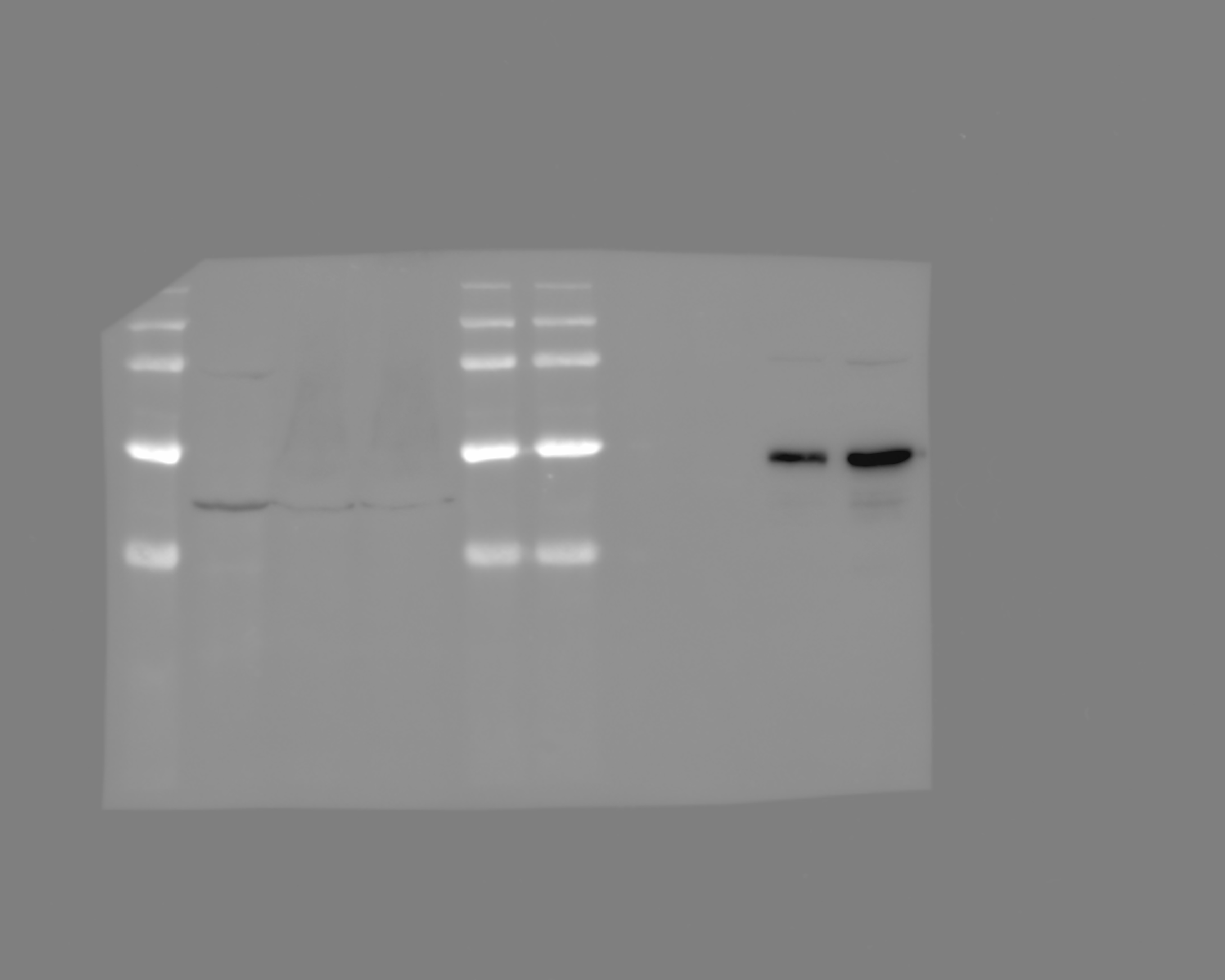

Supplement: Figure 5—source data 1. [file elife-92635-fig5-data1.zip › Figure 5 - source data 1/Figure 5 - source data 5E - RAW/ABHD2.tif]

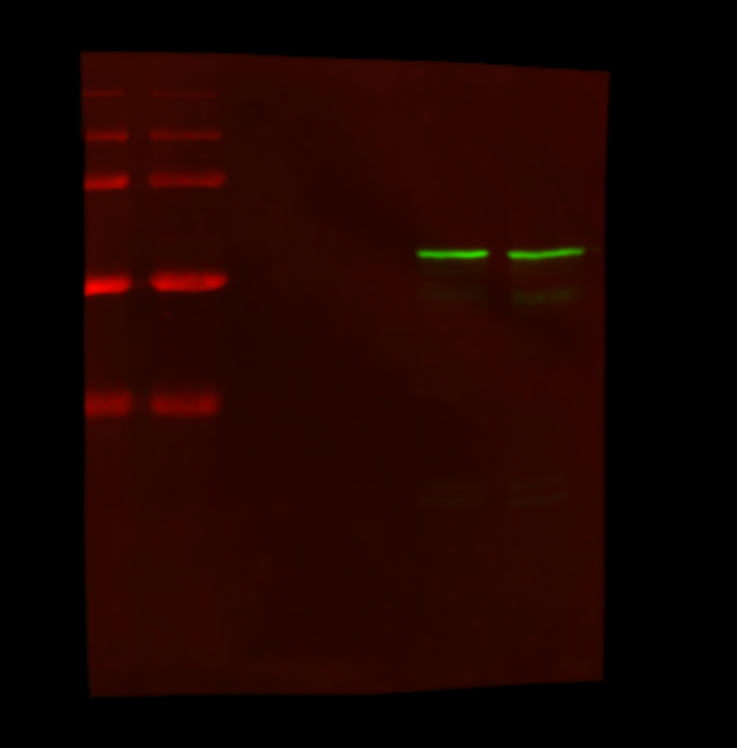

Supplement: Figure 5—source data 1. [file elife-92635-fig5-data1.zip › Figure 5 - source data 1/Figure 5 - source data 5E - RAW/GFP R800.tif]

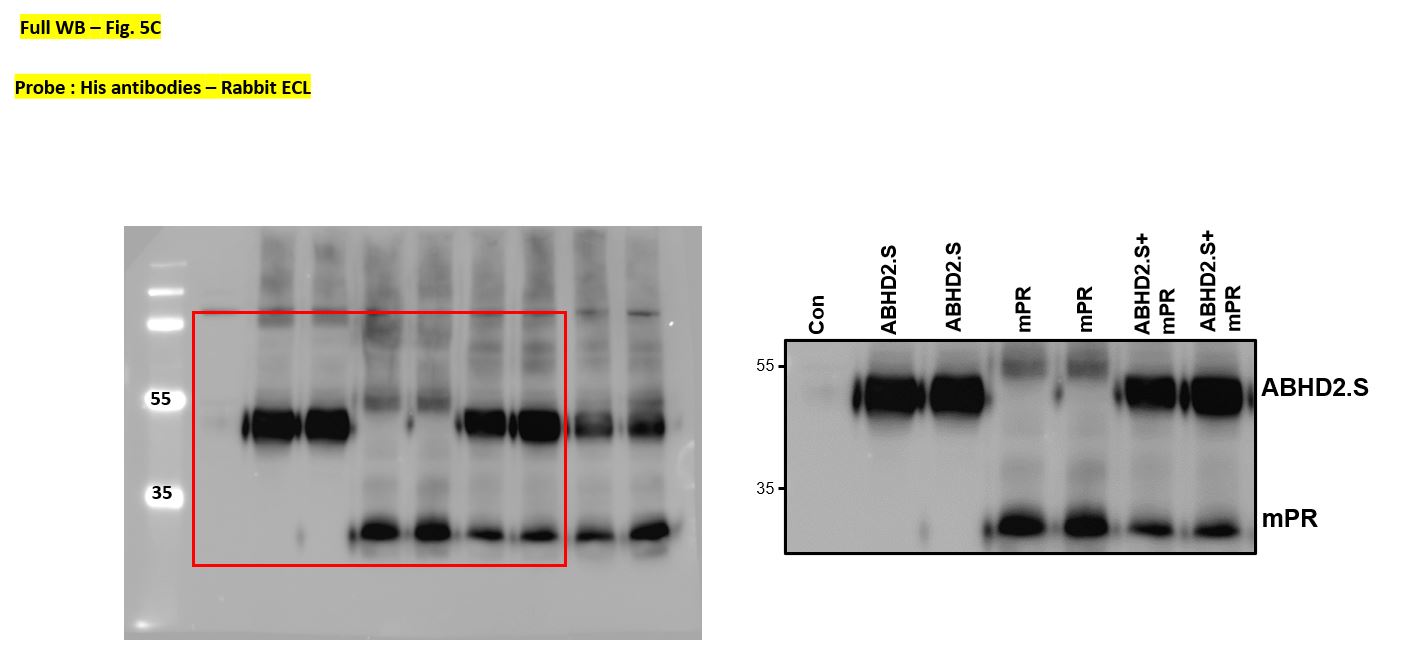

Supplement: Figure 5—source data 2. [file elife-92635-fig5-data2.zip › Figure 5 - source data 2/Figure 5 - source data 5C - Labeled/5C Labeled.JPG]

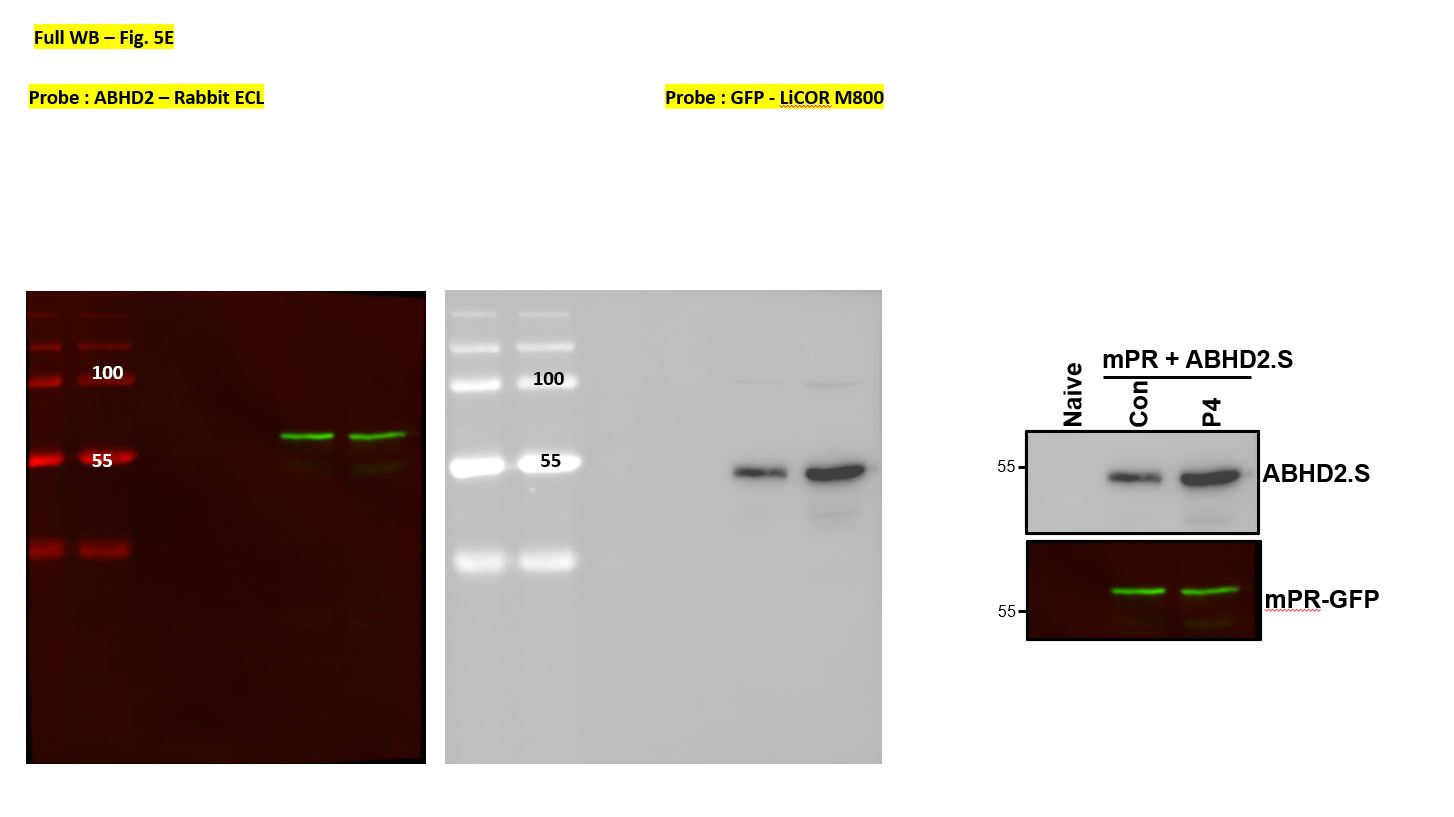

Supplement: Figure 5—source data 2. [file elife-92635-fig5-data2.zip › Figure 5 - source data 2/Figure 5 - source data 5E - Labeled/5E Labeled.JPG]

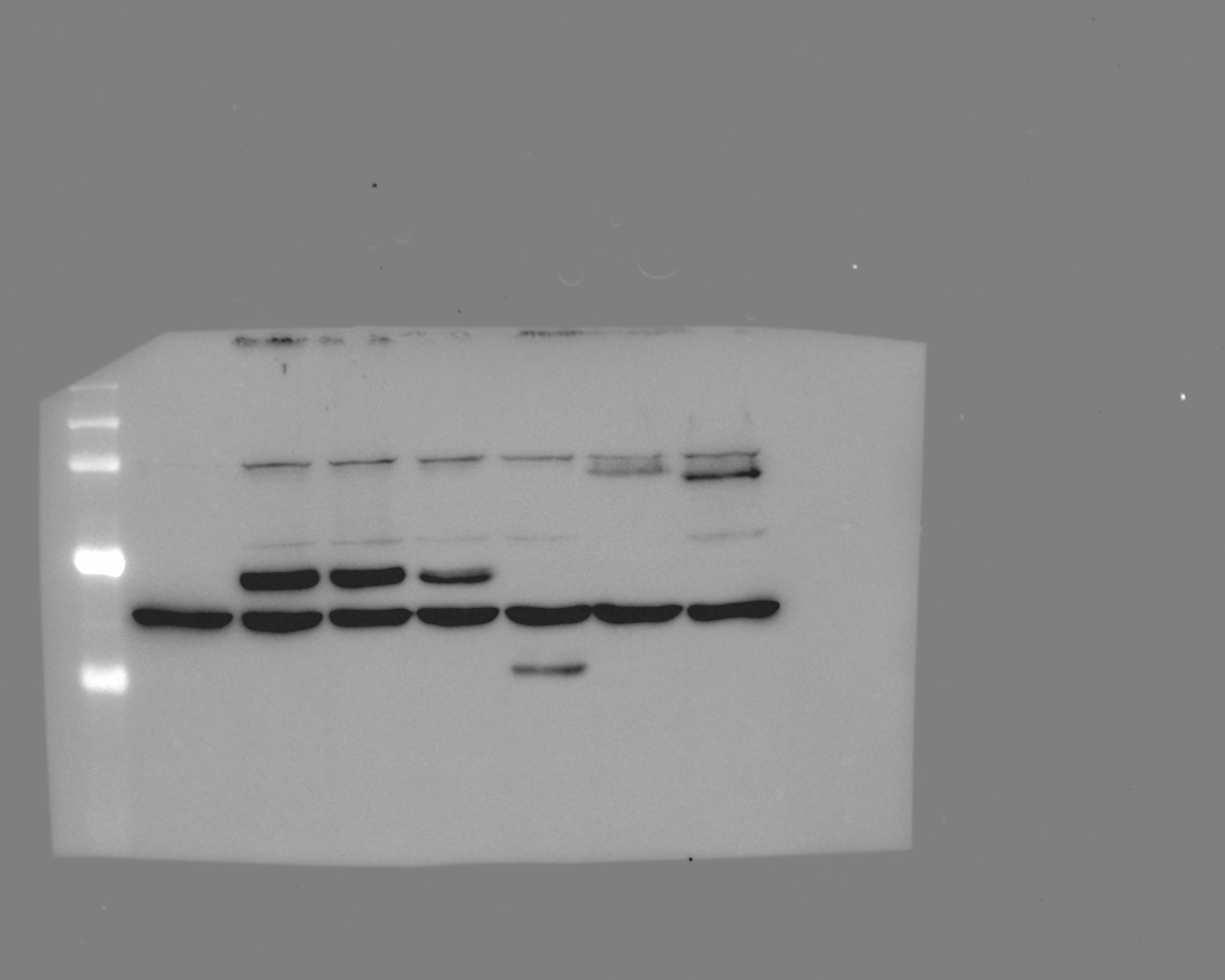

Supplement: Figure 5—figure supplement 1—source data 1. [file elife-92635-fig5-figsupp1-data1.zip › Figure 5 - Figure supplement 5 - source data 1/Figure 5 - Figure supplement 5A - RAW/ABHD2 - Input.tif]

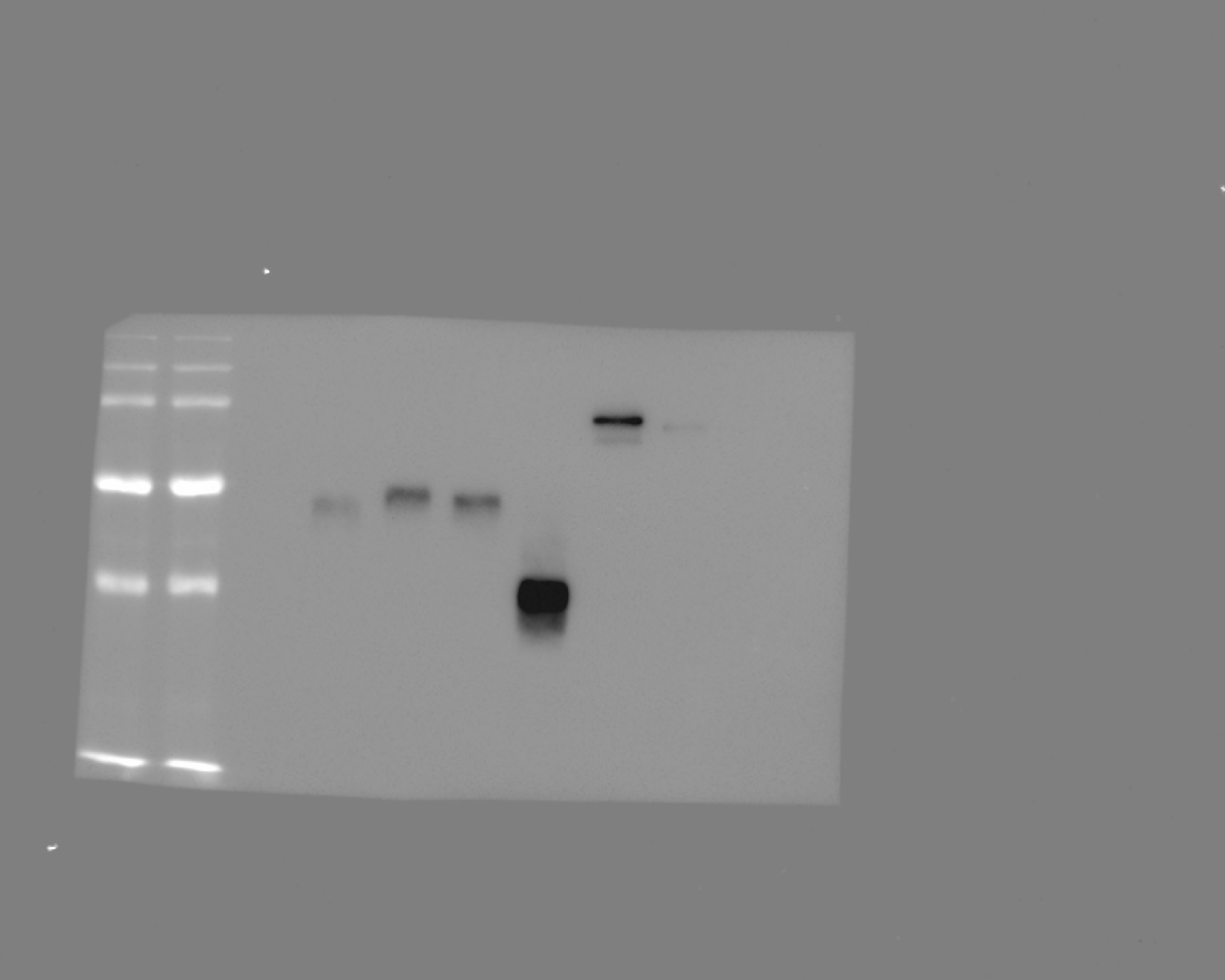

Supplement: Figure 5—figure supplement 1—source data 1. [file elife-92635-fig5-figsupp1-data1.zip › Figure 5 - Figure supplement 5 - source data 1/Figure 5 - Figure supplement 5A - RAW/ABHD2 - IP.tif]

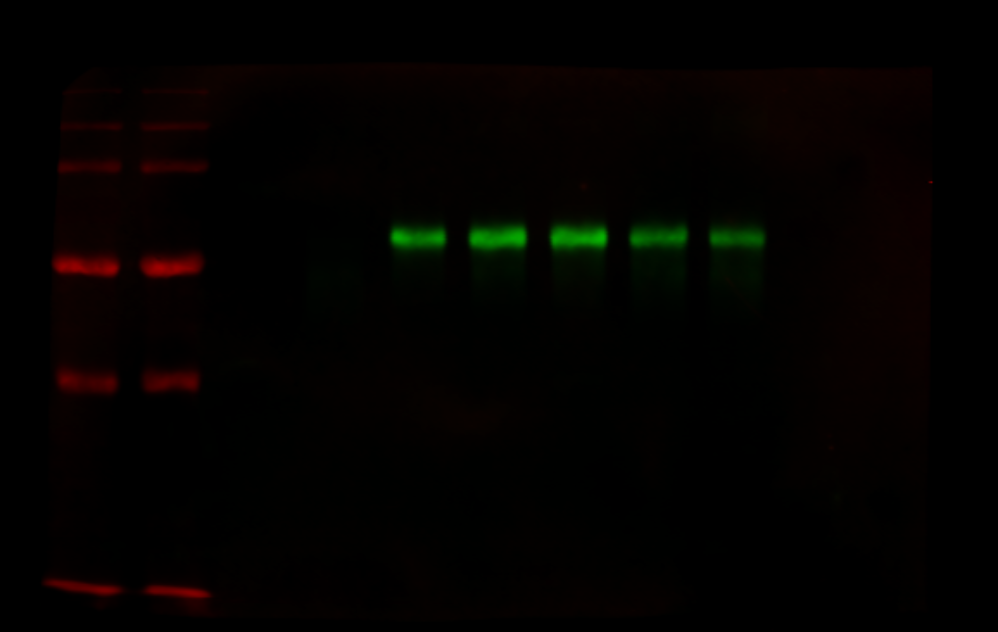

Supplement: Figure 5—figure supplement 1—source data 1. [file elife-92635-fig5-figsupp1-data1.zip › Figure 5 - Figure supplement 5 - source data 1/Figure 5 - Figure supplement 5A - RAW/GFP R800 - IP.tif]

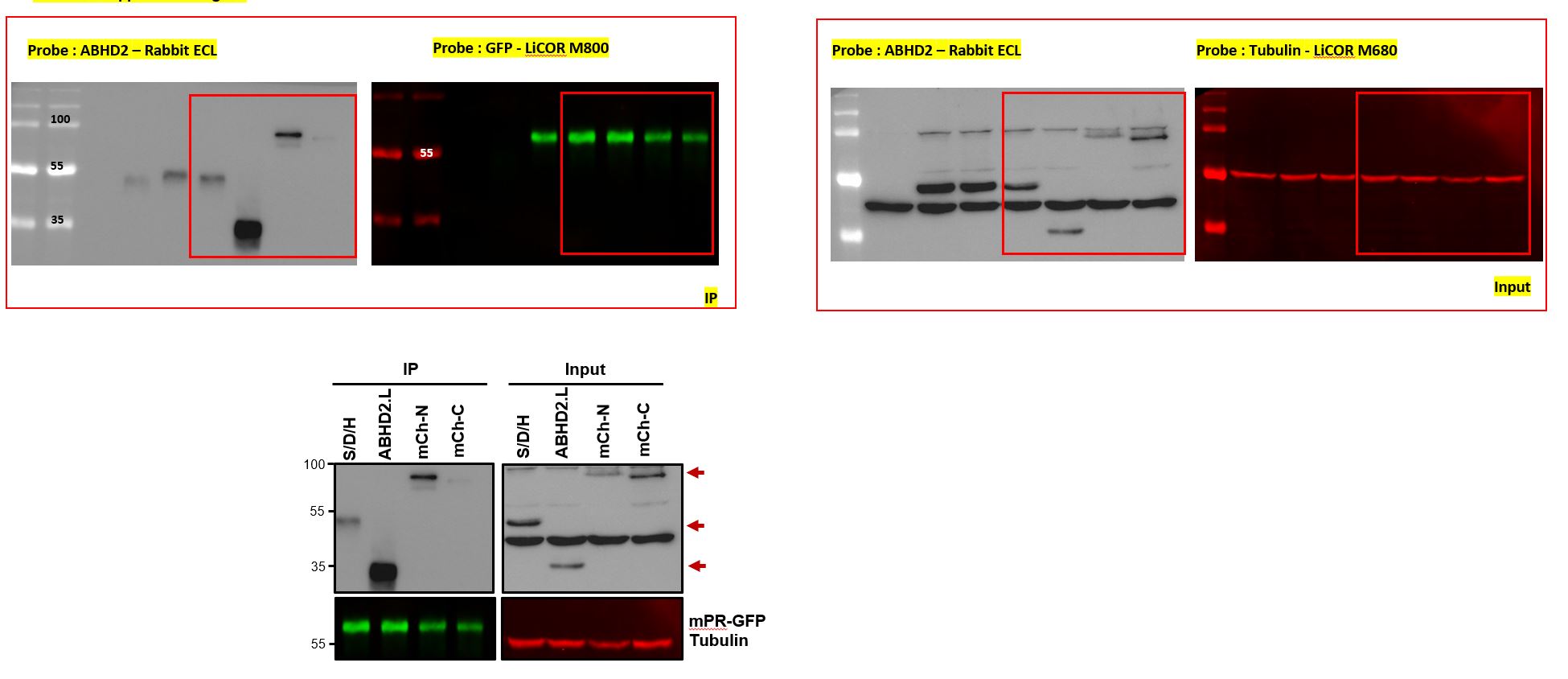

Supplement: Figure 5—figure supplement 1—source data 2. [file elife-92635-fig5-figsupp1-data2.zip › Figure 5 - Figure supplement 5 - source data 2/Figure 5 - Figure supplement 5A - Labeled/Figure 5 - source data Figure supplement 5A - Labeled.JPG]

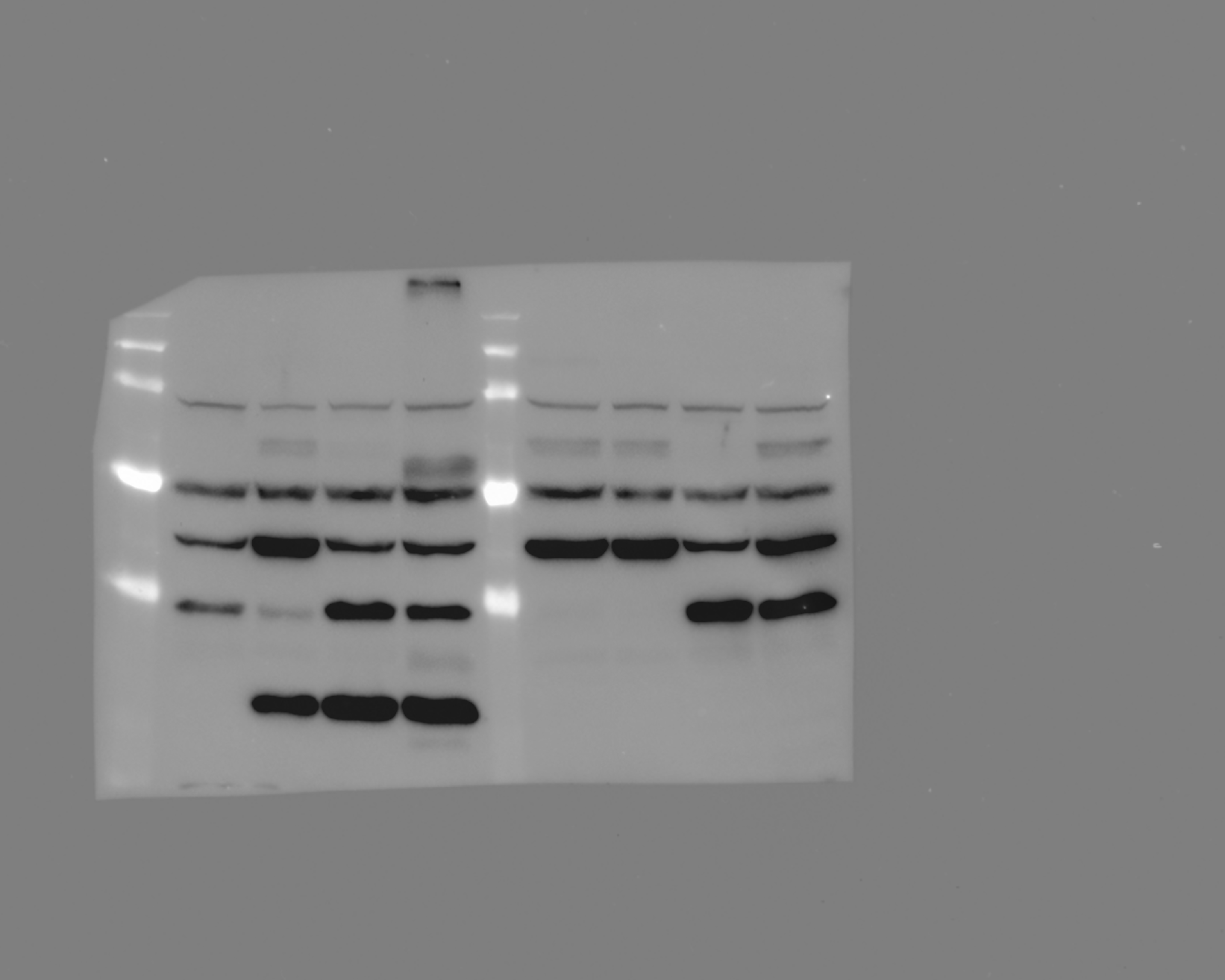

Supplement: Figure 6—source data 1. [file elife-92635-fig6-data1.zip › Figure 6 - source data 1/Figure 6 - source data 6F - RAW/GFP - Tubulin and Snap25.tif]

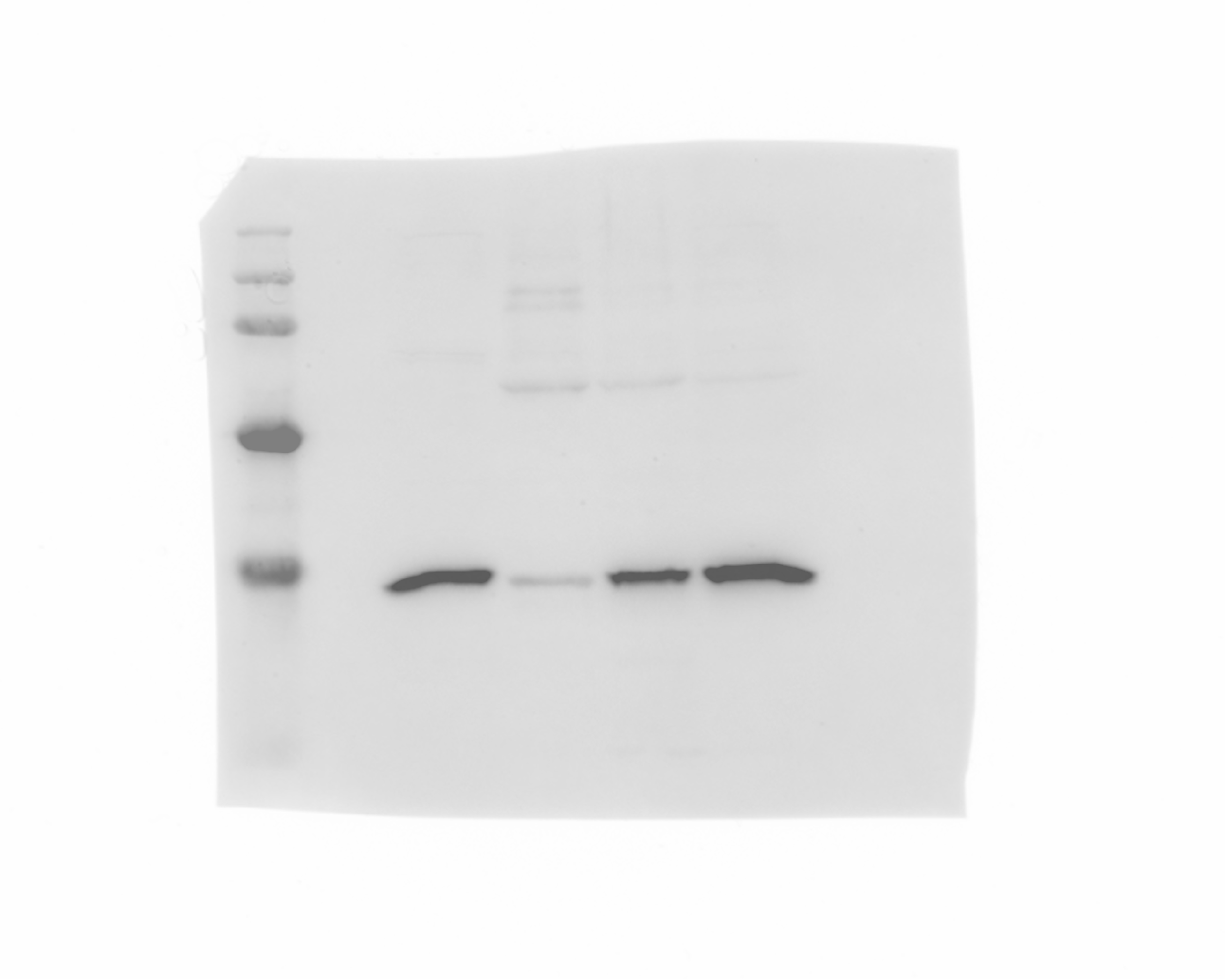

Supplement: Figure 6—source data 1. [file elife-92635-fig6-data1.zip › Figure 6 - source data 1/Figure 6 - source data 6G - RAW/p-cdc2 - 20 sec - section 2.tif]

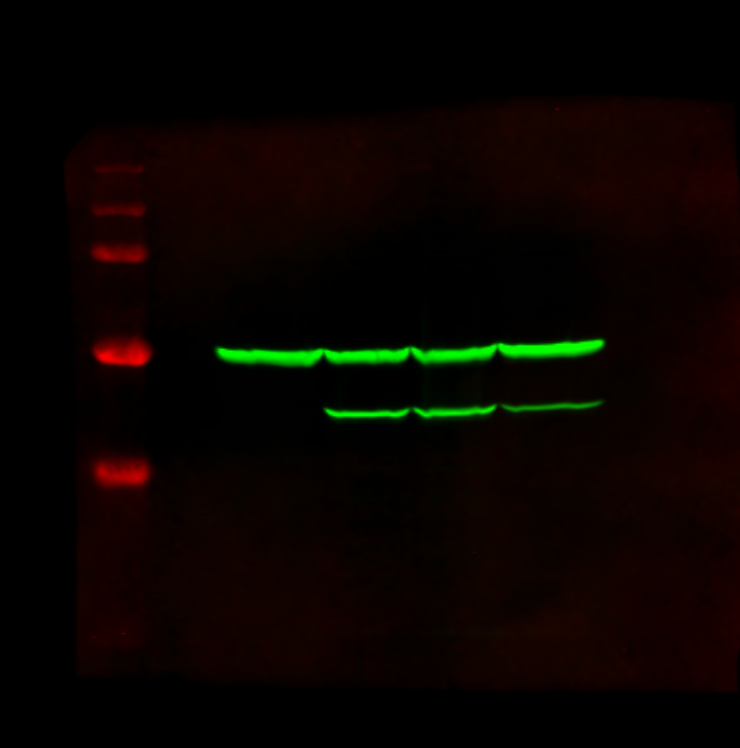

Supplement: Figure 6—source data 1. [file elife-92635-fig6-data1.zip › Figure 6 - source data 1/Figure 6 - source data 6G - RAW/p-MAPK and Tubulin M800 - section 2.tif]

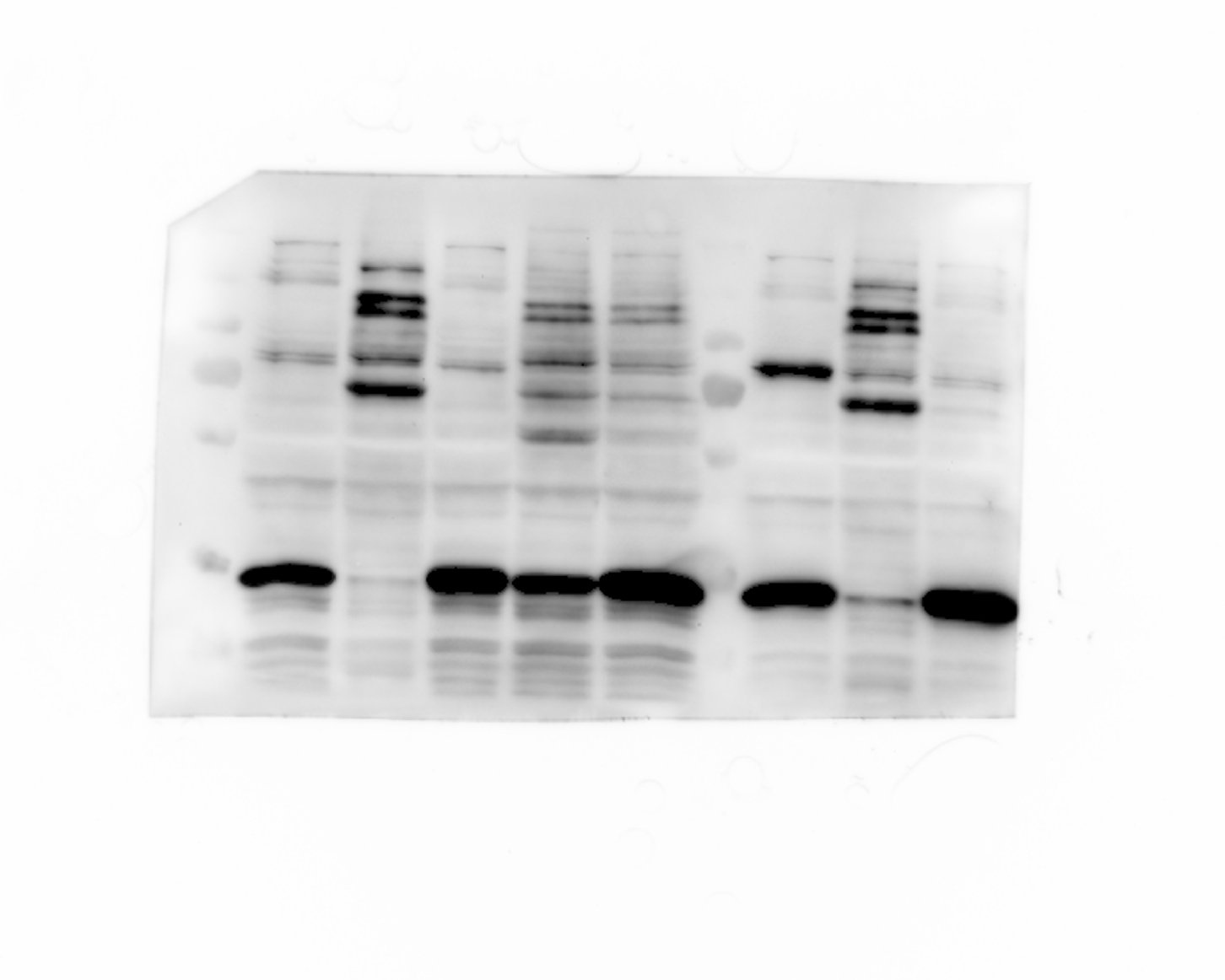

Supplement: Figure 6—source data 1. [file elife-92635-fig6-data1.zip › Figure 6 - source data 1/Figure 6 - source data 6G - RAW/p-plk1 - 1 min - section 1.tif]

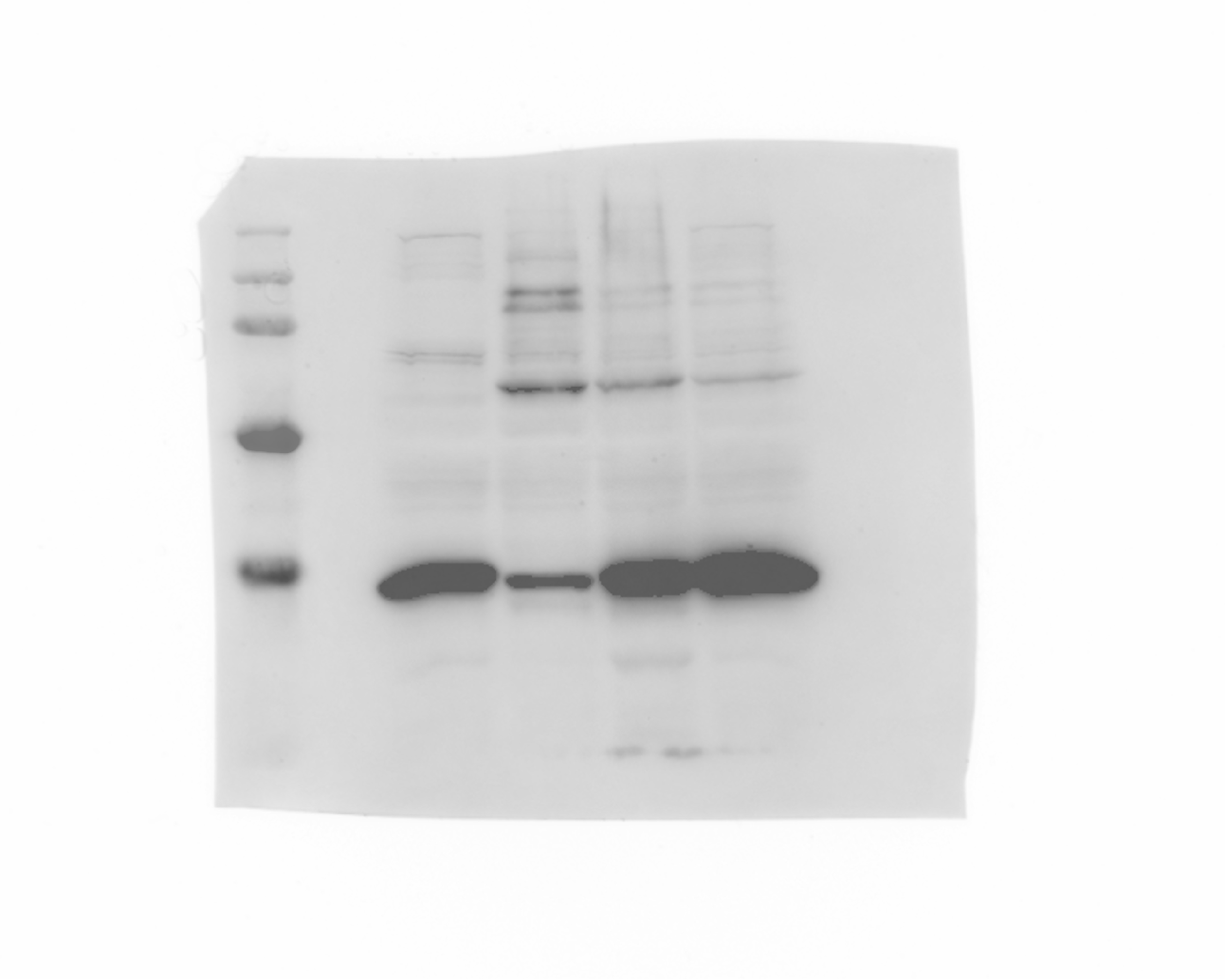

Supplement: Figure 6—source data 1. [file elife-92635-fig6-data1.zip › Figure 6 - source data 1/Figure 6 - source data 6G - RAW/p-plk1 - 1 min - section 2.tif]

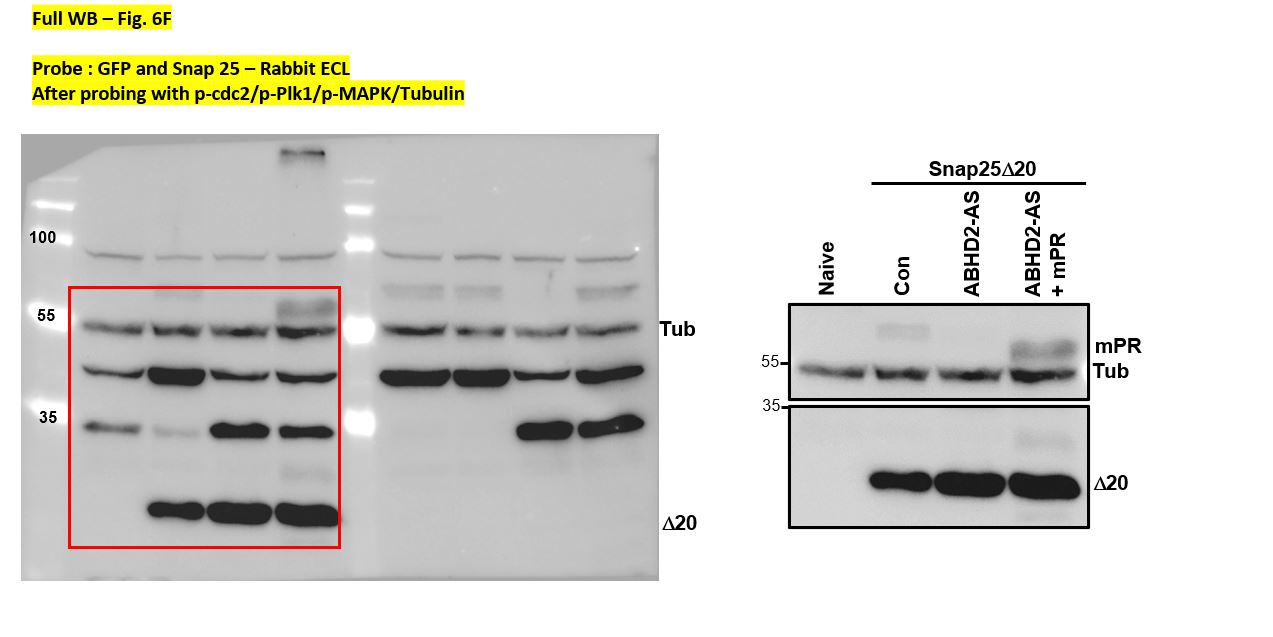

Supplement: Figure 6—source data 2. [file elife-92635-fig6-data2.zip › Figure 6 - source data 2/Figure 6 - source data 6F - Labeled/6F Labeled.JPG]

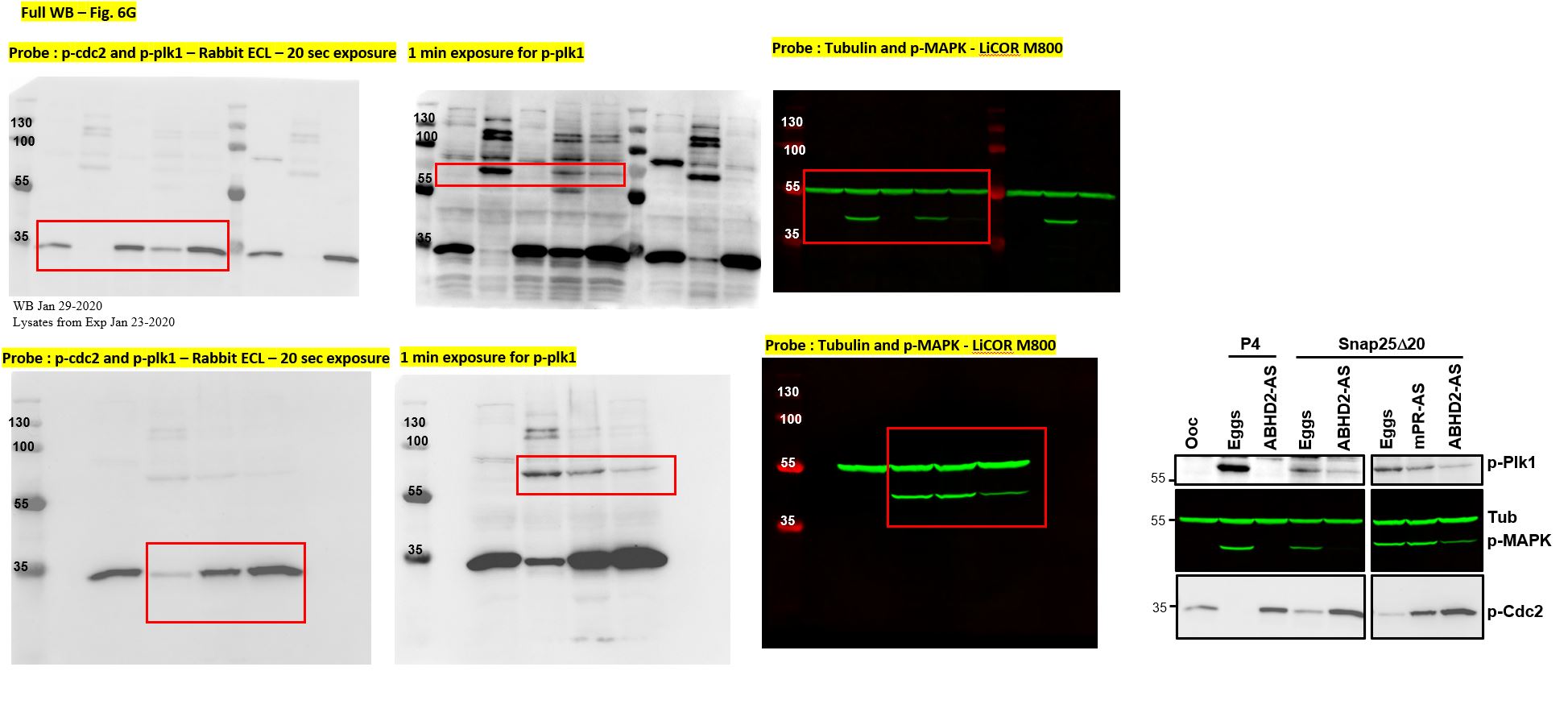

Supplement: Figure 6—source data 2. [file elife-92635-fig6-data2.zip › Figure 6 - source data 2/Figure 6 - source data 6G - Labeled/6G Labeled.JPG]
